# Supplementary material for: Can process mapping and a multisite Delphi of perioperative professionals inform our understanding of system-wide factors that may impact operative risk?
Source: BMJ Open. 2022 Nov 11;12(11):e064105. doi: 10.1136/bmjopen-2022-064105 (PMC9660566; doi:10.1136/bmjopen-2022-064105)
Supplement: Supplementary data [file bmjopen-2022-064105supp001.pdf]

Can process mapping and a multi-site Delphi of perioperative professionals inform our understanding of system-wide factors that may impact operative risk?

Stubbs DJ, Bashford T, GIlder FJ, Nourallah B, Ercole A, Levy N, Clarkson PJ

Supplementary Material:

This document contains supplementary figures, **further material is available as:**

1. A **.xlsx format** spreadsheet showing all suggestion voted on across the three rounds of the Delphi

Contents of this document

1. **Annotated process maps demonstrating the care of elective (a) and emergency (b) vascular surgical patients**
2. **List of risk scores used to provide a 'minimum' dataset for voting on in Delphi round two**
3. **Component variables from these risk scores**
4. **Hierarchy of question groupings used in Delphi rounds two and three**
5. **'Novel' variables from the Delphi**

Supplementary Figure 1: Process map of elective vascular care used to identify perioperative stakeholders.

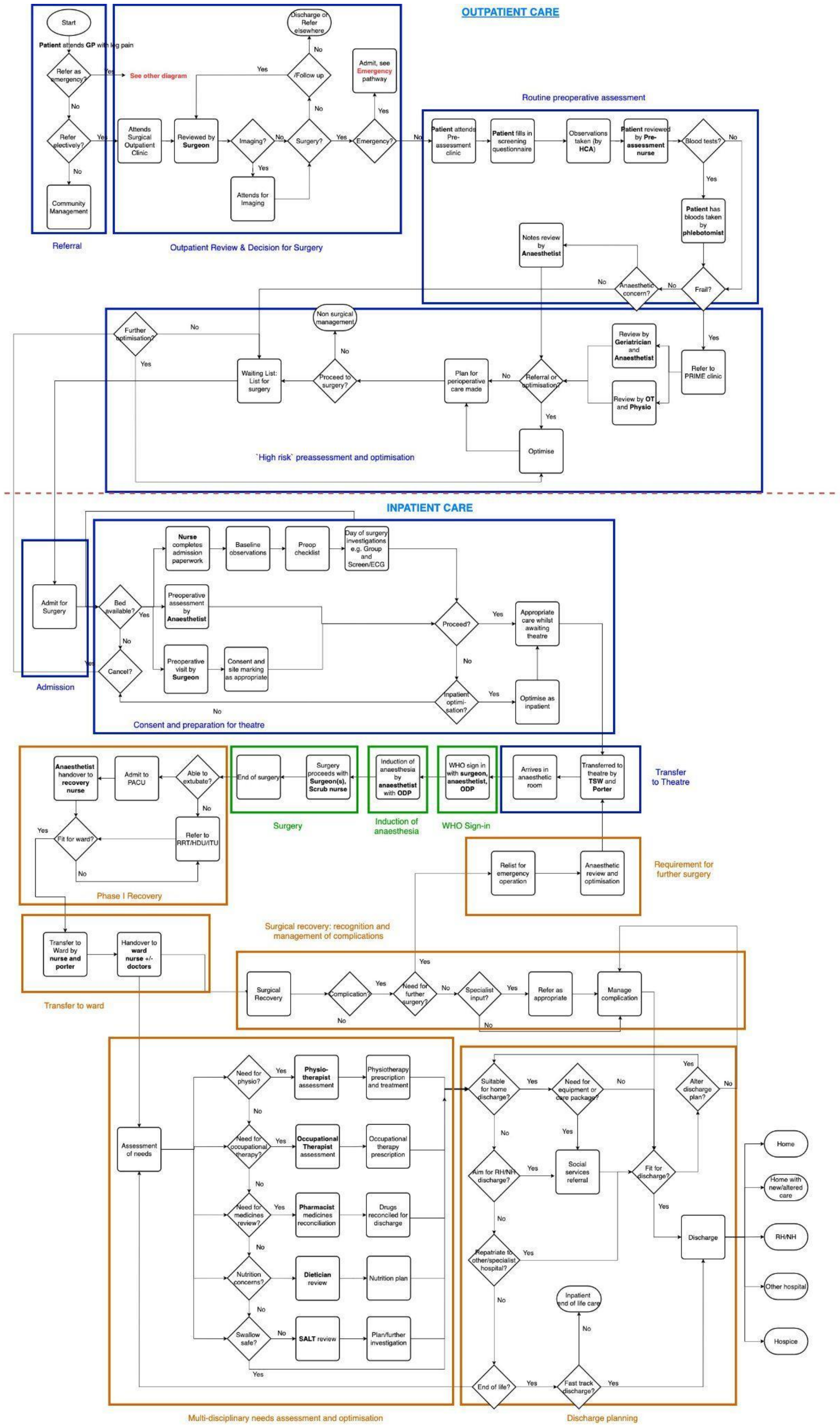

**Supplementary Figure 2: Process map of emergency vascular care used to identify perioperative stakeholders. **

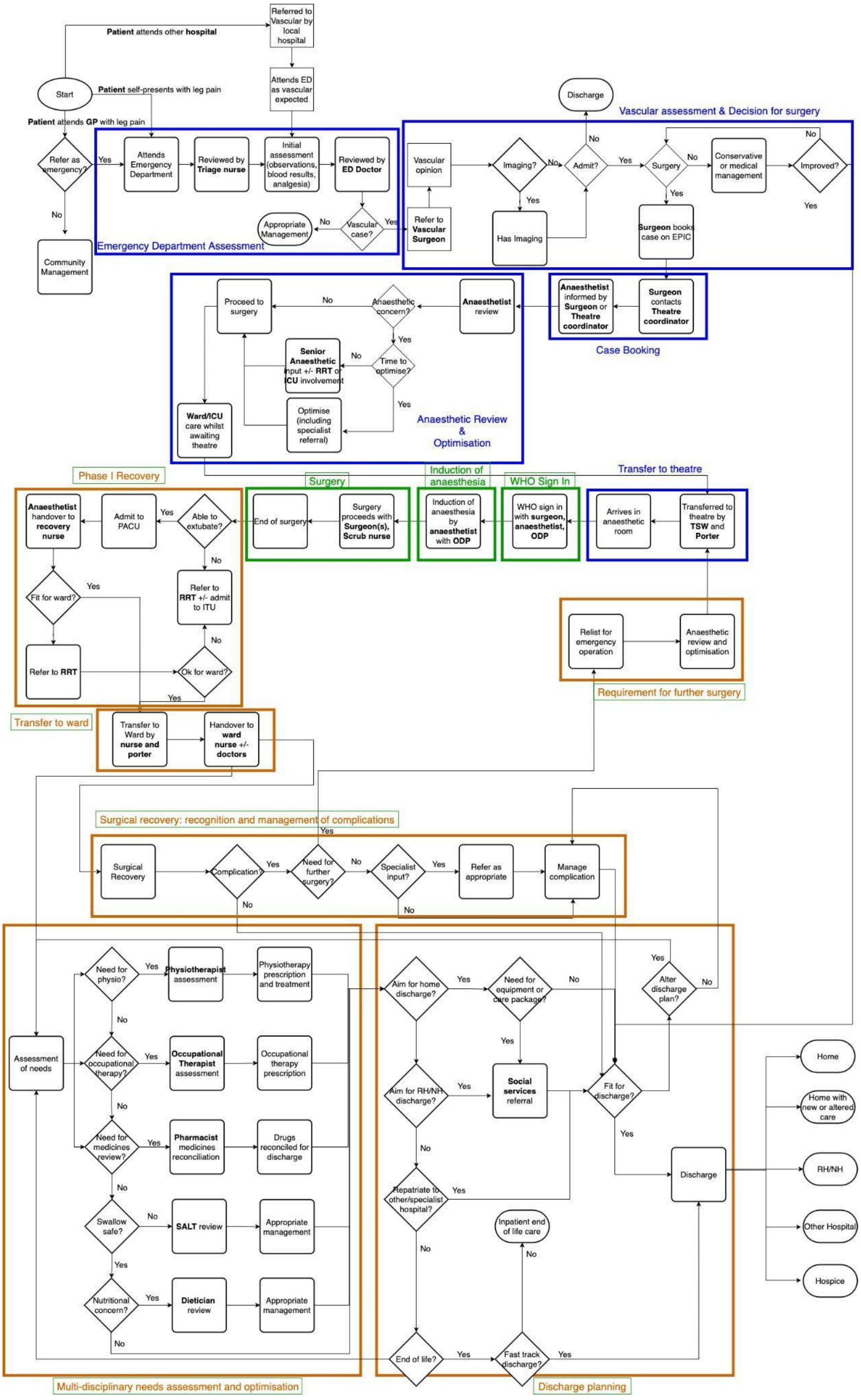

(In both Figure S1 and S2, coloured boxes correspond to abridged care processes shown in Figure 1 in main text)

Risk scores used in formation of a 'minimum' dataset for voting in rounds two and three

**ASA score**
**APACHE**
**BHOM**
**Charlson Comorbidity Index**
**DELAWARE**
**Detsky index**
**E-PASS**
**Edmonton Frail scale**
**ICISS**
**IRIS**
**Maastricht Nutritional Assessment**
**Mini nutritional assessment**
**MPM III**
**Nutritional risk index**
**Otago Surgical outcome score**
**Perioperative Mortality Risk Score**
**POSSUM**
**P-POSSUM**
**RCRI**
**SAPS II**
**Subjective Global assessment**
**Surgical Apgar**
**Surgical mortality score**
**Surgical Risk Scale**
**Surgical Risk Score (Donati)**

Supplementary Table 1: List of risk scores identified as having been validated in heterogeneous surgical cohorts in a 2013 systematic review[1]. These scores were deconstructed (see **Supplementary Figure 3**) to provide the minimum components of any dataset resulting from the Delphi. *ASA = American Society of Anesthesiologists, BHOM – Biochemistry & Haematology Outcome Model, APACHE = Acute physiology & chronic health evaluation, DELAWARE = Dense Laboratory Whole blood applied Risk Estimation, E-PASS = Estimation of physiological ability and surgical stress, ICISS = ICD injury severity score, IRIS = Identification of risk in surgical patients score, MPM III = Mortality prediction model (III), POSSUM = Physiological and Operative Severity Score for the enUmeration of Mortality and morbidity, P-POSSUM = Portsmouth modification of POSSUM, RCRI = Revised cardiac risk index, SAPS II = Simplified acute physiology score II*

1. Moonesinghe SR, Mythen MG, Das P, Rowan KM, Grocott MP. Risk stratification tools for predicting morbidity and mortality in adult patients undergoing major surgery: qualitative systematic review. *Anesthesiology* 2013; **119**: 959–81.

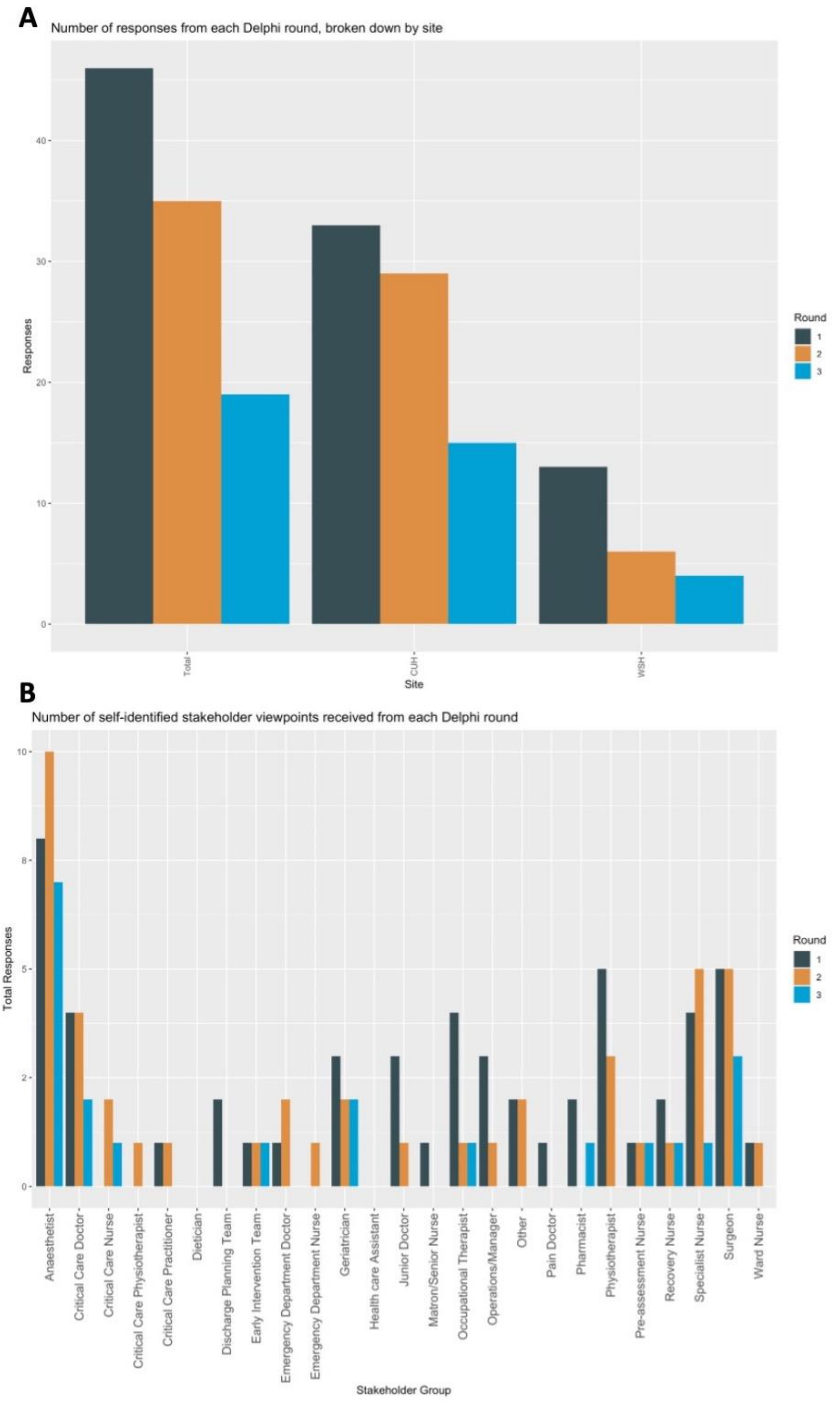

Supplementary Figure 3: Response rate and respondent profile by round for a Delphi exercise to gain a consensus view on factors impacting on outcome in older surgical patients. *CUH = Cambridge University Hospitals (a tertiary care centre), WSH = West Suffolk Hospital (a secondary care centre)*

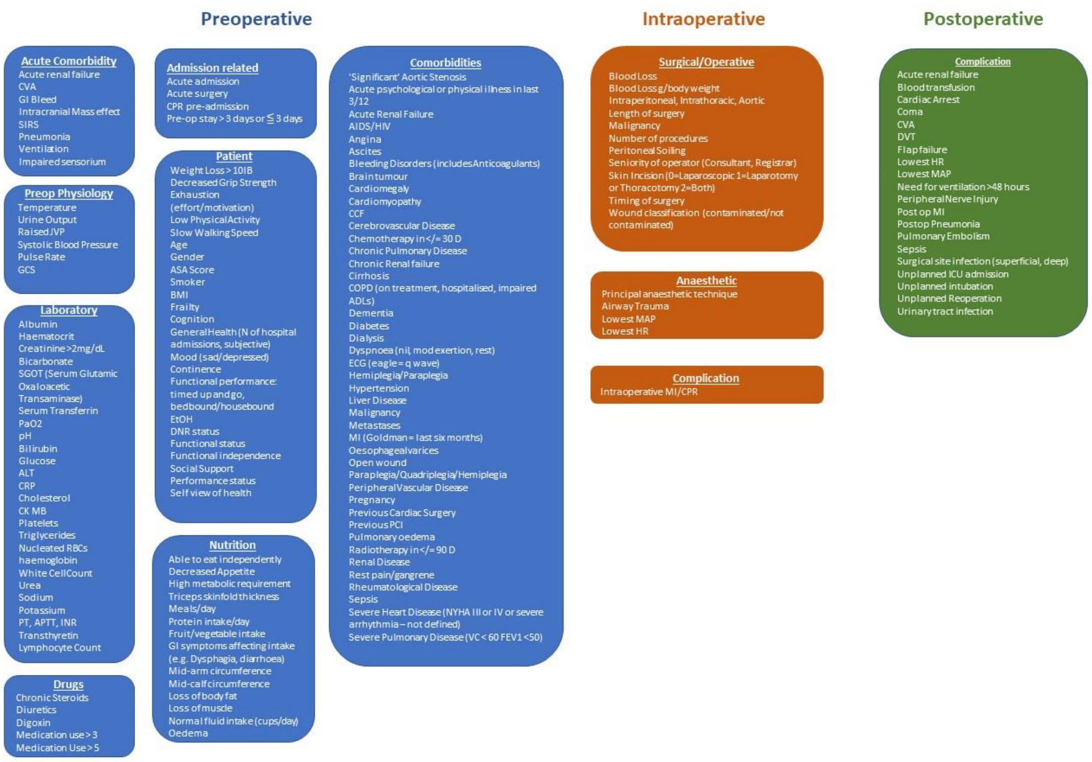

Supplementary Figure 4: Components of validated perioperative scores introduced into Delphi at round two. These variables were identified by deconstructing the risk scores shown in Supplementary Table 1. Groupings are artefactual for clarity and to delineate key temporal aspects of the use of the identified risk scores. Each of these variables were included in Delphi round two, regardless of whether they were present in free-text suggestions from the panel in round one.

Variables suggested by the Delphi panel that did not feature in the development of perioperative risk scores or in a systematic review of structural and process quality indicators.

VARIABLE	DOMAIN	GROUP
**Opioid deprescribing pre-discharge**	Admission	Analgesic Plan
**Opioid dose**	Admission	Analgesic Plan
**Chest injuries**	Admission	Circumstances
**Delayed presentation**	Admission	Circumstances
**Disruption to a patient's routine**	Admission	Circumstances
**Bed Occupancy Rates**	Organisation	Hospital Performance
**Subdural haematoma**	Admission	Circumstances
**Trauma**	Admission	Circumstances
**Indication for surgery (pathology)**	Admission	Details of Surgery
**Orthopaedic v Non-Orthopaedic**	Admission	Details of Surgery
**Delayed Transfer of Care (DToC) Rates**	Organisation	Hospital Performance
**Delayed Transfer of Care**	Admission	Discharge Planning
**ED Admission wait times**	Organisation	Hospital Performance
**Empathy**	Organisation	Staffing
**Enthusiasm and engagement**	Organisation	Staffing
**Health Service Organisation**	Organisation	Health Services Organisation
**Hospital Performance & Strain**	Organisation	Hospital Performance
**Intensive care occupancy**	Organisation	Hospital Performance
**Details of Surgery**	Admission	Intraoperative
**Intraoperative CO2 Control**	Admission	Intraoperative
**Intraoperative Complications**	Admission	Intraoperative
**Medical cover**	Organisation	Staffing
**Intraoperative Physiological Control**	Admission	Intraoperative
**ODP Seniority**	Admission	Intraoperative
**Operator Fatigue**	Admission	Intraoperative

**Scrub nurse Seniority**	Admission	Intraoperative
**Number of outlying patients**	Organisation	Hospital Performance
**Number of patients cared for**	Organisation	Health Services Organisation
**Number of vacant posts**	Organisation	Hospital Performance
**Nursing cover**	Organisation	Staffing
**Occupational therapy cover**	Organisation	Staffing
**Escalation plan made**	Admission	Postoperative
**Organisational culture**	Organisation	Staffing
**Overnight stay in ED**	Organisation	Hospital Performance
**Number of attachments**	Admission	Postoperative
**Pharmacy cover**	Organisation	Staffing
**Physiotherapy cover**	Organisation	Staffing
**Referrals & Reviews**	Admission	Postoperative
**Time to occupational therapist review**	Admission	Postoperative
**Time to pharmacist review (e.g. for medicines reconcilliation)**	Admission	Postoperative
**Anastomotic Breakdown**	Admission	Postoperative Complications
**Practice type**	Organisation	Health Services Organisation
**New diagnosis of chronic conditions (e.g. Dementia)**	Admission	Postoperative Complications
**Poor Wound Healing**	Admission	Postoperative Complications
**Postoperative Dysrhythmia**	Admission	Postoperative Complications
**Postoperative Pain**	Admission	Postoperative Complications
**Postoperative Pneumonia**	Admission	Postoperative Complications
**Postoperative sepsis**	Admission	Postoperative Complications
**DNR status & Escalation plan**	Admission	Preoperative
**How long patients expect to stay in hospital**	Admission	Preoperative
**Primary Care Structure**	Organisation	Health Services Organisation
**Procurement**	Organisation	Policies and Procedures

**Prehabilitation program**	Admission	Preoperative
**Respiratory Virus Rates**	Organisation	Hospital Performance
**Season**	Organisation	Hospital Performance
**Therapy plans**	Admission	Preoperative
**Staffing**	Organisation	Staffing
**Staffing levels**	Organisation	Staffing
**Able to follow commands preoperatively**	Admission	Preoperative Physiology
**Tertiary admission/transfer**	Organisation	Health Services Organisation
**Preoperative CVS impairment**	Admission	Preoperative Physiology
**Preoperative Delirium**	Admission	Preoperative Physiology
**Preoperative Physiology**	Admission	Preoperative Physiology
**Preoperative Renal impairment**	Admission	Preoperative Physiology
**Preoperative Respiratory Failure**	Admission	Preoperative Physiology
**Ward moves/handovers**	Admission	Postoperative

Supplementary Table 2: List of variables that emerged during the Delphi that did not feature in the development of perioperative risk scores or in a systematic review of quality and structural process indicators. For references see text. Blue highlighting demonstrates factors related to the healthcare organisation, Green highlights factors pertaining to the individual admission in question.
