# Supplementary material for: Can process mapping and a multisite Delphi of perioperative professionals inform our understanding of system-wide factors that may impact operative risk?
Source: BMJ Open. 2022 Nov 11;12(11):e064105. doi: 10.1136/bmjopen-2022-064105 (PMC9660566; doi:10.1136/bmjopen-2022-064105)
Supplement: Author's manuscript [file bmjopen-2022-064105.draft_revisions.pdf]

BMJ Open is committed to open peer review. As part of this commitment we make the peer review history of every article we publish publicly available.

When an article is published we post the peer reviewers' comments and the authors' responses online. We also post the versions of the paper that were used during peer review. These are the versions that the peer review comments apply to.

The versions of the paper that follow are the versions that were submitted during the peer review process. They are not the versions of record or the final published versions. They should not be cited or distributed as the published version of this manuscript.

BMJ Open is an open access journal and the full, final, typeset and author-corrected version of record of the manuscript is available on our site with no access controls, subscription charges or pay-per-view fees (http://bmjopen.bmj.com).

If you have any questions on BMJ Open's open peer review process please email
info.bmjopen@bmj.com

BMJ Open

Can process mapping and a multi-site Delphi of perioperative professionals inform our understanding of system-wide factors that may impact operative risk?

Journal:	*BMJ Open*
Manuscript ID	bmjopen-2022-064105
Article Type:	Original research
Date Submitted by the Author:	21-Apr-2022
Complete List of Authors:	Stubbs, Daniel; University of Cambridge, Department of Engineering<br>Gilder, Fay; Princess Alexandra Hospital NHS Trust<br>Nourallah, Basil; West Suffolk Hospital, Department of Anaesthesia<br>Levy, Nicholas; West Suffolk Hospital, Department of Anaesthesia<br>Bashford, Tom; University of Cambridge School of Clinical Medicine, Medicine; University of Cambridge School of Technology, Engineering<br>Ercole, Ari; Cambridge University Hospitals NHS Foundation Trust<br>Clarkson, John; University of Cambridge, Engineering Design Centre
Keywords:	ANAESTHETICS, SURGERY, Risk management < HEALTH SERVICES ADMINISTRATION & MANAGEMENT, QUALITATIVE RESEARCH, STATISTICS & RESEARCH METHODS

SCHOLARONE™
Manuscripts

I, the Submitting Author has the right to grant and does grant on behalf of all authors of the Work (as defined in the below author licence), an exclusive licence and/or a non-exclusive licence for contributions from authors who are: i) UK Crown employees; ii) where BMJ has agreed a CC-BY licence shall apply, and/or iii) in accordance with the terms applicable for US Federal Government officers or employees acting as part of their official duties; on a worldwide, perpetual, irrevocable, royalty-free basis to BMJ Publishing Group Ltd ("BMJ") its licensees and where the relevant Journal is co-owned by BMJ to the co-owners of the Journal, to publish the Work in this journal and any other BMJ products and to exploit all rights, as set out in our licence.

The Submitting Author accepts and understands that any supply made under these terms is made by BMJ to the Submitting Author unless you are acting as an employee on behalf of your employer or a postgraduate student of an affiliated institution which is paying any applicable article publishing charge ("APC") for Open Access articles. Where the Submitting Author wishes to make the Work available on an Open Access basis (and intends to pay the relevant APC), the terms of reuse of such Open Access shall be governed by a Creative Commons licence – details of these licences and which Creative Commons licence will apply to this Work are set out in our licence referred to above.

Other than as permitted in any relevant BMJ Author's Self Archiving Policies, I confirm this Work has not been accepted for publication elsewhere, is not being considered for publication elsewhere and does not duplicate material already published. I confirm all authors consent to publication of this Work and authorise the granting of this licence.

For peer review only

Can process mapping and a multi-site Delphi of perioperative professionals inform our understanding of system-wide factors that may impact operative risk?

Stubbs DJ[1,2a], Bashford T[2ab], Gilder FJ[3] Nourallah B[4], Ercole A[5], Levy N[6], Clarkson PJ[7]

1: Wellcome Trust Research Fellow, Healthcare Design Group, Department of Engineering, University of Cambridge, Trumpington Street, Cambridge, CB2 1PZ.
2: a) Honorary Specialist Registrar in Anaesthesia, b) Clinical Lecturer in Anaesthesia, University Division of Anaesthesia, Department of Medicine, University of Cambridge, Addenbrooke's Hospital, Hills Road, Cambridge, CB2 0QQ,
3: Medical Director, Princess Alexandra Hospitals Harlow, NHS Trust, Hamstel Road, Harlow, Essex, CM20 1QX
4: Core Trainee, Department of Anaesthesia, West Suffolk Hospital, Bury St Edmunds, Hardwick Lane, IP33 2QZ
5: Consultant Anaesthetist & Intensivist, University Division of Anaesthesia, Department of Medicine, University of Cambridge, Addenbrooke's Hospital, Hills Road, Cambridge, CB2 0QQ
6: Consultant Anaesthetist, Department of Anaesthesia, West Suffolk Hospital, Bury St Edmunds, Hardwick Lane, IP33 2QZ
7: Professor of Engineering Design, Healthcare Design Group, University of Cambridge Department of Engineering, Trumpington Street, Cambridge, CB2 1PZ

Correspondence to:

Dr DJ Stubbs
djs225@cam.ac.uk

Short Title: A Delphi study to understand perspectives on perioperative risk

KEYWORDS

- Delphi Technique
- Risk prediction
- Perioperative Care
- Electronic Health Records
- Older people
- Unwarranted variation

Twitter Handles

@djramsaystubbs, @ari_ercole, @nicholaslevy, @fayjojo

Word count (excluding abstract, references, tables and figure legends): 3741

For peer review only

ABSTRACT

Objectives: To examine whether the use of process mapping and a multidisciplinary Delphi can identify potential contributors to perioperative risk. We hypothesised that this approach may identify factors not represented in common perioperative risk tools and give insights of use to future research in this area.

Design: Multidisciplinary modified Delphi study

Setting: Two centres (one tertiary, one secondary) in the United Kingdom during 2020 amidst coronavirus pressures.

Participants: 91 stakeholders from 23 professional groups involved in the perioperative care of older patients. Key stakeholder groups were identified through the use of process mapping of local perioperative care pathways.

Results: Response rate ranged from 51% in round one to 19% in round three. After round one, free text suggestions from the panel were combined with variables identified from perioperative risk scores. This yielded a total of 410 variables that were voted on in subsequent rounds. Including new suggestions from round two, 468/519 (90%) of the statements presented to the panel reached a consensus decision by the end of round three. Identified risk factors included patient level factors (such as ethnicity and socio-economic status); and organisational or process factors related to the individual hospital (such as policies, staffing, and organisational culture). 66/160 (41%) of the new suggestions did not feature in systematic reviews of perioperative risk scores or key process indicators. No factor categorised as 'organisational' is currently present in any perioperative risk score.

Conclusions: Through process mapping and a modified Delphi we gained insights into additional factors that may contribute to perioperative risk. Many were absent from currently used risk stratification scores. These results enable an appreciation of the contextual limitations of currently used risk tools and could support future research into the generation of more holistic datasets for the development of perioperative risk assessment tools.

Strengths and Weaknesses

- Novel use of process mapping to identify key perioperative stakeholders
- Multidisciplinary Delphi panel to gain breadth of perspective
- Performed across two sites
- Comprehensive results may be of use to other researchers designing perioperative research databases
- Results may be limited by low response rate in final round (although majority of consensus decisions made in round two)

For peer review only

Introduction

Understanding, predicting, and communicating risk is fundamental to perioperative practice [1]. The use of surgical risk stratification tools is a cornerstone of programs such as the National Emergency Laparotomy Audit (NELA) [2]. When using the output of such tools in day-to-day practice it is important to remember that they are developed from external electronic datasets and their application to decisions at the level of an individual centre or patient is complex and fraught with difficulty [3]. Risk scores commonly consist of patient and surgical factors that are statistically associated with relevant outcomes [4]. However, performance of such scores is far from perfect [5]. This raises the question as to whether currently unmeasured factors may improve our understanding and prognostication of risk. There is strong evidence that this is the case. Unwarranted variation is widespread within the National Health Service (NHS), with between-centre differences leading to discrepancies in cost, efficiency, and most importantly, patient outcome [6]. Significantly, organisational or system factors have been shown in a recent analysis of NELA data to be associated with worse outcomes [7]. However, such factors do not feature in commonly used risk assessment tools

Healthcare is increasingly digitised with electronic health records (EHRs) increasingly capturing detailed events from across the hospital system. In the perioperative setting, EHRs may hold data pertaining to an individual's baseline state, operation details, physiological responses under anaesthesia, and perioperative complications. EHRs therefore appear to offer an appealing substrate to identify and test factors associated with perioperative outcome. However, in reality, due to the complexity of modern healthcare, the data they hold doesn't accurately capture the 'true' patient state but the record is in fact biased by the care processes involved in the recording of such data [8]. To use EHRs to identify nascent factors that may broaden our understanding of perioperative risk, including at the level of the healthcare system, it is vital that we understand how that care is delivered, and the affect this might have on the electronic record. However, electronic records are themselves complex, with a multitude of both implicit and explicit data types.

A system may be defined as a collection of elements serving a common purpose, with emergent behaviour arising through their interaction. Such systems may be considered as simple, complicated, or complex, with complex systems exhibiting certain key behaviours such non-linearity, non-scalability, path dependence and emergence[9]. Healthcare has been posited as just such a complex system, with a perioperative system possibly including pre-assessment clinics, operating theatres, staff from multiple disciplines, patients, equipment, consumables, care processes, and culture. All of these may generate data which could provide novel insights into our understanding of risk. Researchers in other healthcare settings, where similar causal pathways may exist, have already recognised that standard statistical techniques may not be comprehensive enough to interrogate and understand such systems [10]. Therefore there is a need to consider additional strategies such as those used to understand and design other complex systems [11,12].

For peer review only

Given the complexity of healthcare, our aim in this paper was to employ a systems approach [11] to develop a holistic dataset that a breadth of perioperative professionals felt captured all dimensions of perioperative risk. The output of such an endeavour would enable a rational approach to extracting information from EHRs for future research and enable a clearer understanding of how this data is captured as part of the wider care process. Such strategies as well as offering a clearer scientific rationale for future hypothesis testing, could also encourage better data governance – by only extracting data fields from the EHR that were felt to be of clear importance.

To develop our consensus dataset, we employed a stepwise approach. Firstly, we sought to visualise how perioperative care is delivered through process mapping. This is a technique which enables complex systems to be visualised as a series of steps representing decision-making points and pathways that has been used in various healthcare settings [13,14]. These process maps were then used to identify the range of perioperative professionals involved in patient care, and whose views we needed to capture. Using this list of 'stakeholders' we next conducted a modified Delphi [15] across two hospital sites seeking to gain consensus on the breadth of factors at both patient, operation, and system level that were felt to impact on patient outcome.

Methods

Setting and approvals:

Participants were recruited from two UK hospital trusts utilising EHRs. Cambridge University Hospitals Trust (CUH) is a tertiary referral centre offering secondary and tertiary level surgical services to patients across the East of England. The West Suffolk Hospital (WSH) is a district general hospital based in Bury St Edmunds. WSH offers a range of secondary care services whilst referring patients for tertiary care to specialist centres including CUH. This study forms part of the 'Designing Improved Surgical Care for Older people' (DISCO) study, approved by the London and Surrey Borders Research Ethics Committee (reference 19/LO/1648). DISCO is jointly sponsored by the University of Cambridge and CUH.

Methodological approach and techniques:

Systems engineers use a variety of techniques to interrogate and design complex systems. A framework for applying these tools to the healthcare setting has been recently jointly published by the UK's Royal Academy of Engineers, Royal College of Physicians, and Academy of Medical Sciences [11]. In this study we utilised brainstorming interviews and graphical elicitation[16], process mapping, and a Delphi process to ensure we captured the views of professionals (stakeholders) from across the perioperative system in two distinct sites. A project flow diagram demonstrating the sequential use of these techniques is shown in **Figure 1.**

Stakeholder identification and process mapping:

We formed a local steering group of experienced perioperative professionals. The group consisted of a consultant anaesthetist, geriatrician, and surgeon alongside a senior physiotherapist, occupational therapist, matron, and operations manager. Brainstorming interviews were conducted to identify stakeholder groups who should be represented on the Delphi panel. Interviews were structured around

For peer review only

the iteration of process maps representing a stereotypical ‘high risk’ surgical patient undergoing vascular surgery. Vascular surgery was chosen due to the ability to draw comparisons between elective and emergency cases as well as the need for clinical input from a range of perioperative professionals. Stakeholders were identified from these maps and then chosen for representation on the Delphi panel if at least one member of the steering group felt this was appropriate.

Delphi panel formation:

Representatives were approached from each stakeholder group across both sites aiming for a complementary spread of sub-specialities between sites. Individuals were approached by lead researchers in each site, provided with written information, and gave informed consent prior to each Delphi round.

Delphi structure – round one:

The Delphi consisted of three rounds and was distributed using an online survey tool (Qualtrics - www.qualtrics.com). In round one individuals were asked to provide free-text suggestions on what they felt *‘Could contribute to a poor-outcome in an older patient undergoing surgery’.* We defined a poor-outcome to be where an individual lost their independence after surgery or suffered a complication (such as a myocardial infarction). Suggestions from the panel were combined with known important risk factors identified from a systematic review of perioperative risk scores [4]. All risk factors (literature and panel suggestions) were voted on in the second and third rounds using a five-point Likert scale. Participants could also provide free-text comments and clarifications. A minority of questions in round two asked for specific free-text responses to expand on suggestions or provide relevant cut-off points (e.g. frailty score thresholds). New suggestions from round two were included for voting in round three. Before each major group of suggestions (e.g. ‘comorbidities’) respondents were asked to indicate on the same five point Likert scale how ‘measurable’ suggestions within this category were felt to be. This was to gauge the feelings of the panel on the practicality of this information being captured within an electronic record..

Delphi structure – rounds two and three:

All potential risk factors were presented for voting in a hierarchy consisting of groups and subgroups reflecting free-text suggestions. There were three broad domains - patient level factors (*that may have existed prior to an individual’s current admission: e.g. comorbidities)*, admission level factors (*the circumstances and events occurring in any given admission*), and system level factors (*suggestions that were related to the structure or running of a service within a healthcare organisation*). Tracking of free text data for generating round two and three questionnaires was conducted using ATLAS.ti (www.atlasti.com) with quantitative analysis conducted in R v 3.6.3 [17].

Definition of consensus:

Consensus was defined using criteria modified from a Delphi of quality indicators for patients with traumatic brain injury [50]. Given the relative heterogeneity of our panel, consensus for a given question required at least 50% of respondents to address it, a median score of >3.5, and an interquartile range (IQR) of ≤ 1. Consensus exclusion at the end of round two was stricter, with this requiring a score of <2.5, an IQR of ≤ 1, and no scores of 5 (‘very important’).

For peer review only

Comparing to common literature sources

At the conclusion of the Delphi all novel, consensus, suggestions were compared to a systematic review of perioperative structure and process indicators[18] as well as whether they had been examined in the statistical development of each risk score[19–32]. This approach was chosen to ensure that our final list of factors contained a solid core of important patient level factors whilst allowing us to critique novel findings against a comprehensive literature source.

Groupings, definitions, and results reporting.

Certain questions in the Delphi allowed participants to vote on cut-points (e.g. "*a 'high-risk' body mass index (BMI) is above 30*") or on an overarching concept that could encompass multiple factors (for instance 'complications' could encompass 'perioperative myocardial infarction' as well as surgical complications). For transparency all questions are presented in the **supplementary spreadsheet file** and included in relevant numerators or denominators within the results section. However, these cut-offs were not considered when reporting on factors present in literature risk scores unless they were explicitly mentioned (e.g. a risk score defined a specific cut-off).

Patient and public involvement in project:

The protocol for our project was reviewed by the CUH patient and public involvement panel in November 2017 and received favourable responses. The aims of our project are clearly aligned with the James Lind associations priority setting partnership conducted with the National Institute of Academic Anaesthesia [33].

Results

System mapping.

Process maps representing the care of an elective and emergency vascular surgical patient were generated based on an understanding of local care pathways (abridged version in **Figure 2**, full examples as **supplementary figures 1 & 2**). These diagrams were revised based on brainstorming interviews with our steering group. Maps were used to identify relevant professional roles (stakeholders) across the perioperative pathway. In total, 52 unique staff stakeholder groups were identified. Of these, 33 were nominated by at least one member of the steering group for representation on the Delphi panel (**Figure 3**).

Stakeholders and Delphi participants

Invitations to participate were sent to 91 professionals, identified by leads in both trusts (63 from CUH 28 from WSH). These covered 23 broad professional groups as well as subspecialty expertise. Numbers of representatives are shown in **Supplementary Figure 3**. Participants were able to contribute views from more than one perspective (e.g. anaesthetist AND intensive care doctor). Response rates ranged from 51% (*n=46*) for the first round to 19% (*n = 17*) in the third round. Conduct of the Delphi was significantly impacted by the coronavirus pandemic - distribution of the second and third rounds was delayed by six

months due to the first wave and the final round was completed as case pressures built prior to the second national UK lockdown in November 2020.

Minimum dataset: variables from the literature

25 risk scores were identified from a 2013 systematic review [4]. A full list of scores and their component variables are demonstrated in **supplementary table 1** and **supplementary figure 3**. Any of the variables not suggested in round one were included by default for voting in rounds two and three.

Delphi round one:

From the literature, 168 variables (representing specific measurements or characteristics) were identified (**Supplementary figure 3**). From free-text responses from participants, 411 suggestions for variables were identified including 243 unique or refined definitions from those present in the literature. 80 of the 168 (48%) literature variables were not suggested by the panel in round one. Suggestions included

To provide context to questions in later rounds, all suggestions were grouped into a framework separating out suggestions pertaining to the *patient*, their *admission,* and the *organisation* caring for them. For clarity of questioning, suggestions were further organised into related groups (such as comorbidities), sub-groups (e.g. cardiovascular comorbidities), and then a granular level that represented specific concepts or definitions (e.g. ‘electrocardiogram changes’ and ‘left bundle branch block’). This structure is demonstrated in **Figure 4** and is available to view interactively in the **supplementary material.** Distribution of new suggestions across these domains of *patient, admission, and organisation* is shown in **Figure 5A.** None of the suggestions that appeared to pertain to health system organisation or performance (**Table 1**) featured in currently used risk scores (**Figure 5A).**

**Suggested factor**	**Question group**
Availability of services	Health Services Organisation
Number of patients cared for *(by each GP)*	Health Services Organisation
GP practice type	Health Services Organisation
Presence of a telephone advice service	Health Services Organisation
Tertiary admission/transfer	Health Services Organisation

For peer review only

Bed Occupancy Rates	Hospital Performance
Delayed Transfer of Care (DToC) Rates	Hospital Performance
ED Admission wait times	Hospital Performance
Intensive care occupancy	Hospital Performance
Number of outlying patients	Hospital Performance
Number of vacant posts	Hospital Performance
Overnight stay in ED	Hospital Performance
Respiratory Virus Rates	Hospital Performance
Season	Hospital Performance
Surgery cancellation rates	Hospital Performance
Care Pathway Documents	Policies and Procedures
Drug availability (e.g. anticoagulant reversal agents)	Policies and Procedures
Enhanced recovery programs	Policies and Procedures
Procedures for minimising conflicts between teams	Policies and Procedures
Procurement	Policies and Procedures
Empathy	Staffing
Enthusiasm and engagement	Staffing
Expertise in the care of older people	Staffing

For peer review only

Medical cover	Staffing
Nursing cover	Staffing
Occupational therapy cover	Staffing
Organisational culture	Staffing
Pharmacy cover	Staffing
Physiotherapy cover	Staffing
Staff traits, skills, behaviours	Staffing
Staffing levels	Staffing
Training levels	Staffing

Table 1: Examples of 'organisational' factors suggested by Delphi participants as being important in the determination of outcome for older surgical patients. All of these suggestions were made by participants in the first Delphi round and reached consensus across round two or three.

Delphi rounds two and three:

In round two suggestions were presented for voting with participants able to vote on all levels of the questionnaire hierarchy (**Figure 4**) including groups and subgroups. In total 409 suggestions including 17 groups, 34 subgroups, and 358 variables (of which 117 were operationalised definitions of a variable - such as 'left bundle branch block' representing an 'important ECG change') were voted on. Two suggestions from the risk scores (cut-off values for defining polypharmacy) were held until round three as free-text suggestions for this threshold were sought from panellists in round two. Full details of suggestions that were presented for voting across the final two rounds, including number of responses and consensus thresholds, are available as a **supplementary spreadsheet file**.

At the conclusion of round two, 357 (87%) of statements reached consensus criteria for inclusion. Analysis of free text suggestions identified a further 108 suggested variables for voting in the third Delphi round.

For peer review only

The median score for measurability in round two was four [IQR:3-4]. Laboratory results were felt to be the most measurable (attracting a median score of five) with patient *beliefs and behaviours*, *health service organisation, hospital performance & strain,* and *policies & procedures* attracting median scores of three.

In the third Delphi round, 110 of 158 (70%) variables met the consensus criteria for inclusion, one variable (*"shortness of breath on strenuous exercise"*) met the criteria for exclusion. In total, across both rounds, 468 of 519 (90%) suggestions that were presented to the panel reached consensus decisions (**Figure 5B**). In round three median measurability was put at four [IQR:4-5] with only *beliefs & behaviours* and *health service organisation* attracting median scores of less than four. Across both latter rounds all of the suggestions encompassing health system organisation reached the definition for consensus.

Comparison to literature variables

To assess whether suggested variables might have been previously examined but excluded from published risk scores due to a lack of statistical significance we identified the original papers generating each of the risk scores[19–32]. Twenty-five (7%) of the 351 unique suggestions from rounds one and two had been previously examined at an earlier phase in the development of at least one risk tool (details in **Supplementary spreadsheet**).

All novel suggestions were also compared against a list of process and structure indicators from a 2018 systematic review[18]. 106 of 351 (30%) novel suggestions could be mapped against one or more metrics identified in this paper. When new suggestions defining patient level characteristics (e.g., comorbidities) were excluded - 87 of 160 (54%) new suggestions relating to admission circumstances or organisational function were represented. Variable that did not appear included markers of system performance (e.g., *number of vacant posts, delayed transfer of care rates)* and staffing (e.g., *occupational therapy cover*) as well as examples of postoperative complications (e.g., *anastomotic breakdown*). For ease of reference these variables are shown in **supplementary table S2** but full details can be seen in the **supplementary spreadsheet file.**

Discussion

This study demonstrates the views of multidisciplinary clinicians on factors felt to influence perioperative risk. We feel that both our findings and methods will be of interest to researchers within the perioperative field as well as in other disciplines seeking to rationally use EHRs for research and improvement. Our results highlighted factors that were not surgical or patient characteristics but instead related to in-hospital events, organisational structure, and hospital performance. Such suggestions were conspicuously absent from commonly used perioperative risk scores **(Figure 5A)** [4] but are compatible with work demonstrating inter-centre variation in outcome [7]. Our final list of consensus variables (available in supplementary) is likely to be of use to researchers in the field seeking to intelligently curate data from their own EHRs, the fact that the panel also voted on specific cut-points (e.g., 'Clinical Frailty Scale of > 5') may also be of use in those seeking to develop relevant triage points within guidelines.

For peer review only

Methodologically we hypothesised that process mapping may enable us to identify a panel of stakeholders whose expertise captured all facets of perioperative care and, that in doing so, we may gain novel insights. This approach is at least partially vindicated in that most factors voted on (351/519) were suggested by the panel. Future work is needed to validate the importance of these findings in understanding whether these in-hospital processes affect or predict patient outcome. An additional strand of research should explore how an understanding of system structure (captured using a process map) may aid researchers in appreciating how care processes may impact on broader data capture within the HER.

The importance of 'non-patient' level factors in prognostication has not been thoroughly explored, perhaps reflecting the challenges in recording such concepts electronically. When considering quantitative data from EHRs, improved risk assessment can be seen by incorporating implicit information such as the timing of blood samples, as well as their resulting value [34]. Here, this timing information is presumably recognising a potentially otherwise undocumented recognition of a clinically unwell patient by a diligent clinician taking, and interpreting, blood samples out of hours. The results of this study suggest that variables not captured in a hospital EHR, such as detailed aspects of social circumstances, may be important. A machine learning model containing only 'sociomarkers' (derived from place of residence) has yielded comparable performance in the prediction of childhood asthma exacerbation compared to the use of patient-level data [35]. However, conflicting findings have been shown in other settings. Although qualitative data derived from patient interviews has suggested that social support and psychological state are influences on individual readmissions with heart failure[36], small studies looking to incorporate questionnaire derived markers have failed to demonstrate improved prognostication [37,38] despite the apparent use of 'unstructured' free-text data in another study [39]. Perioperatively it is well known that clinician judgement improves the performance of commonly utilised risk tools such as SORT (Surgical Outcome Risk Tool) [40]. What is unclear is what additional factors this clinician judgement encapsulates and whether it may reflect an appreciation or implicit judgement of some of the factors identified in this study.

A prerequisite for validating these suggestions in future work is identifying whether valid electronic surrogates exist. One approach to try and address this may be to broaden the definition as to what we view as constituting 'healthcare data'. The Institute of Medicine suggests that relevant ancillary data sources may include human resources records and patient complaints [41]. This could conceivably aid with the incorporation of factors such as staffing levels with complaints data, if adequately coded using tools such as the Healthcare Complaints Analysis Tool (HCAT), identifying poor staff-patient relationships and communication [42].

This study took the unique step of drawing on techniques from systems engineering to structure a Delphi survey of professionals. These methods will be of direct relevance to those involved in informatics, research, or quality improvement both within and beyond perioperative practice. The methods and results of this work also reflects the relevance of systems thinking in healthcare. A systems approach is a way of addressing problems holistically, aware of the interaction between elements and subsequent

unexpected behaviour[11,43]. Suggestions from the panel related to the circumstances of admission, hospital performance, and external pressures. A conceptual framework is thus that the ultimate outcome of any patient stay depends on the relative interaction between factors at different levels. Conceptually this hierarchical agreement is appealing with the importance of external factors, such as coronavirus pressures, a key part of current daily practice.

Beyond our unique methodological approach we feel that a further point of originality within our work is that the Delphi panel were specifically asked to suggest factors that could result in a loss of independence on discharge, a key concern of patients undergoing surgery [44]. Despite this it is not widely considered in perioperative risk stratification[4]. An additional strength of our results is the multidisciplinary nature of the Delphi panel with a plurality of perspectives offering detailed insight into potentially important factors across the perioperative pathway, from drug management to home circumstances and medical management.

We do however acknowledge the fall off in response rate across rounds (51% in round one to 19% in round three). This arose due to the significant pressures of the coronavirus pandemic and that many stakeholder groups on our panel were undertaking additional clinical duties and redeployment to intensive care. However, we would counter this by highlighting the breadth of specialties surveyed, and that the response rate in round two was still 38% and that a majority of consensus factors (357/490) were identified in this round. It is possible that the low response rate in round three skewed the remaining factors towards reaching consensus, but this should make our resultant list of variables especially sensitive (albeit potentially less specific). The high rates of consensus could also reflect true strength of feeling but may have arisen due to issues with questionnaire length, panel composition, and response rates.

Conclusion

This study demonstrates the feasibility of using systems engineering tools to identify and engage clinicians in identifying factors felt to impact on patient relevant outcomes after surgery. The results themselves highlight that these professionals identify non-patient level factors as modulators of perioperative risk. Further work is needed to develop electronic surrogates for these suggestions and validate their significance in real datasets so that their relevance as drivers or markers of unwarranted variation can be assessed.

Acknowledgements:

This research was funded, in whole or in part, by the Wellcome Trust, Grant number: 220542/Z/20/Z (to DJS). A CC BY or equivalent licence is applied to the Author Accepted Manuscript (AAM) arising from this submission, in accordance with the grant's open access conditions.

PJC and NL both contributed equally to this work and should be considered joint senior author

This research was supported by the NIHR Cambridge Biomedical Centre (BRC 1215 20014). The views and opinions expressed by authors in this publication are those of the authors and do not necessarily reflect those of the NHS, the NIHR, or the department of health.

Data Sharing Statement. Summary results for each round of our study are available in the supplementary material.

Competing Interests Statement: No competing interests declared

References:

1 General Medical Council. Decision making and consent. 2020. https://www.gmc-uk.org/-/media/documents/gmc-guidance-for-doctors---decision-making-and-consent-english_pdf-84191055.pdf (accessed 30 Nov 2021).

2 Nela. NELA Risk Calculator. https://data.nela.org.uk/riskcalculator/

3 Lewis G. Next Steps for Risk Stratification in the NHS. NHS England 2015. https://www.england.nhs.uk/wp-content/uploads/2015/01/nxt-steps-risk-strat-glewis.pdf (accessed 14 Dec 2021).

4 Moonesinghe SR, Mythen MG, Das P, *et al.* Risk stratification tools for predicting morbidity and mortality in adult patients undergoing major surgery: qualitative systematic review. *Anesthesiology* 2013;**119**:959–81.

5 Kumar A, Karmarkar A, Downer B, *et al.* Current Risk Adjustment and Comorbidity Index Underperformance in Predicting Post-Acute Utilization and Hospital Readmissions After Joint Replacements: Implications for Comprehensive Care for Joint Replacement Model. *Arthritis Care Res* 2017;**69**:1668–75.

6 Lord Carter of Coles. Operational productivity and performance in English NHS acute hospitals: Unwarranted variations. Department of Health 2016. https://assets.publishing.service.gov.uk/government/uploads/system/uploads/attachment_data/file/499229/Operational_productivity_A.pdf (accessed 7 Dec 2021).

7 Oliver CM, Bassett MG, Poulton TE, *et al.* Organisational factors and mortality after an emergency laparotomy: multilevel analysis of 39 903 National Emergency Laparotomy Audit patients. *Br J Anaesth* 2018;**121**:1346–56.

8 Hripcsak G, Albers DJ. Next-generation phenotyping of electronic health records. *J Am Med Inform Assoc* 2013;**20**:117–21.

9 Ladyman J, Lambert J, Wiesner K. What is a complex system? *Eur J Philos Sci* 2013;**3**:33–67.

10 Stronks K, Nicolaou M. Embracing complexity in social epidemiology. *The Lancet Public Health* 2018;**3**:e352–3.

11 Clarkson PJ, Bogle D, Dean J, *et al. Engineering better care: a systems approach to health and care design and continuous improvement*. London: : Royal Academy of Engineering 2017.

For peer review only

12 Rutter H, Savona N, Glonti K, *et al.* The need for a complex systems model of evidence for public health. *Lancet* 2017;**390**:2602–4.

13 Forrester JA, Koritsanszky LA, Amenu D, *et al.* Developing Process Maps as a Tool for a Surgical Infection Prevention Quality Improvement Initiative in Resource-Constrained Settings. *J Am Coll Surg* 2018;**226**:1103-1116.e3.

14 Antonacci G, Reed JE, Lennox L, *et al.* The use of process mapping in healthcare quality improvement projects. *Health Serv Manage Res* 2018;**31**:74–84.

15 Abbott TEF, Fowler AJ, Pelosi P, *et al.* A systematic review and consensus definitions for standardised end-points in perioperative medicine: pulmonary complications. *Br J Anaesth* 2018;**120**:1066–79.

16 Crilly N, Blackwell AF, Clarkson PJ. Graphic elicitation: Using research diagrams as interview stimuli. *Qual Res* 2006;**6**:341–66.

17 R Core Team. *R: A Language and Environment for Statistical Computing*. Vienna, Austria: : R Foundation for Statistical Computing 2013.

18 Chazapis M, Gilhooly D, Smith AF, *et al.* Perioperative structure and process quality and safety indicators: a systematic review. *Br J Anaesth* 2018;**120**:51–66.

19 Detsky AS, Abrams HB, Forbath N, *et al.* Cardiac Assessment for Patients Undergoing Noncardiac Surgery: A Multifactorial Clinical Risk Index. *Arch Intern Med* 1986;**146**:2131–4.

20 Donati A, Ruzzi M, Adrario E, *et al.* A new and feasible model for predicting operative risk. *Br J Anaesth* 2004;**93**:393–9.

21 Hadjianastassiou VG, Tekkis PP, Poloniecki JD, *et al.* Surgical Mortality Score: Risk Management Tool for Auditing Surgical Performance. *World J Surg* 2004;**28**:193–200.

22 Haga Y, Ikei S, Ogawa M. Estimation of Physiologic Ability and Surgical Stress (E-PASS) as a New Prediction Scoring System for Postoperative Morbidity and. 1999;:219–25.

23 Guigoz Y, Vellas B. The Mini Nutritional Assessment (MNA) for grading the nutritional state of elderly patients: presentation of the MNA, history and validation. *Nestle Nutr Workshop Ser Clin Perform Programme* 1999;**1**:116–22.

24 Nathanson BH, Higgins TL, Kramer AA, *et al.* Subgroup mortality probability models: Are they necessary for specialized intensive care units? *Crit Care Med* 2009;**37**:2375–86.

25 Pillai SB, Van Rij AM, Williams S, *et al.* Complexity- and risk-adjusted model for measuring surgical outcome.

26 Story DA, Fink M, Leslie K, *et al.* Original Papers Perioperative mortality risk score using pre- and postoperative risk factors in older patients. 2009;**37**:392–8.

27 Scott S, Lund JN, Gold S, *et al.* An evaluation of POSSUM and P-POSSUM scoring in predicting post-operative mortality in a level 1 critical care setting. 2014;:1–7.

For peer review only

28 Mercaldo ND, Jacques PJS, D M. Expansion of the Surgical Apgar Score across All Surgical Subspecialties as a Means to Predict Postoperative Mortality. 2011.

29 Lee TH, Marcantonio ER, Mangione CM, *et al.* Derivation and prospective validation of a simple index for prediction of cardiac risk of major noncardiac surgery. *Circulation* 1999;**100**:1043–9.

30 Gil Cebrian J, Bello Cámara MP, Diaz-Alersi R. Apache Ii. *Intensive Care Med* 1987;**13**:143.

31 Gawande AA, Kwaan MR, Regenbogen SE, *et al.* An Apgar Score for Surgery. *J Am Coll Surg* 2007;**204**:201–8.

32 Prytherch DR, Sirl JS, Weaver PC, *et al.* Towards a national clinical minimum data set for general surgery. *Br J Surg* 2003;**90**:1300–5.

33 Niaa, The James Lind Alliance. Anaesthesia and Perioperative Care Priority Setting Partnership: most popular questions. https://www.niaa.org.uk/PSP

34 Agniel D, Kohane IS, Weber GM. Biases in electronic health record data due to processes within the healthcare system: retrospective observational study. *BMJ* 2018;**361**:k1479.

35 Shin EK, Mahajan R, Akbilgic O, *et al.* Sociomarkers and biomarkers: predictive modeling in identifying pediatric asthma patients at risk of hospital revisits. *npj Digital Medicine* 2018;**1**:1–5.

36 Retrum JH, Boggs J, Hersh A, *et al.* Patient-identified factors related to heart failure readmissions. *Circ Cardiovasc Qual Outcomes* 2013;**6**:171–7.

37 Krumholz HM, Chaudhry SI, Spertus JA, *et al.* Do Non-Clinical Factors Improve Prediction of Readmission Risk?: Results From the Tele-HF Study. *JACC Heart Fail* 2016;**4**:12–20.

38 Ahmad FS, French B, Bowles KH, *et al.* Incorporating patient-centered factors into heart failure readmission risk prediction: A mixed-methods study. *Am Heart J* 2018;**200**:75–82.

39 Watson AJ, O'Rourke J, Jethwani K, *et al.* Linking electronic health record-extracted psychosocial data in real-time to risk of readmission for heart failure. *Psychosomatics* 2011;**52**:319–27.

40 Wong DJN, Harris S, Sahni A, *et al.* Developing and validating subjective and objective risk-assessment measures for predicting mortality after major surgery: An international prospective cohort study. *PLoS Med* 2020;**17**:e1003253.

41 Aspden P, Corrigan JM, Wolcott J, *et al. Patient Safety:: Achieving a New Standard for Care Editors, Committee on Data Standards for Patient Safety*. 2004.

42 Gillespie A, Reader TW. The Healthcare Complaints Analysis Tool: Development and reliability testing of a method for service monitoring and organisational learning. *BMJ Qual Saf* 2016;**25**:937–46.

43 Komashie A, Ward J, Bashford T, *et al.* Systems approach to health service design, delivery and improvement: a systematic review and meta-analysis. *BMJ Open* 2021;**11**:e037667.

44 Ladha KS, Wijeysundera DN. Role of patient-centred outcomes after hospital discharge: a state-of-the-art review. *Anaesthesia* 2020;**75 Suppl 1**:e151–7.

For peer review only

For peer review only

Figure Legends

Figure 1: Project stages. Process mapping was performed with a steering group of experienced professionals, with maps iterated between brainstorming interviews. At the conclusion of this stage two outputs were generated – the maps themselves and a stakeholder diagram showing the viewpoints we sought to capture in our Delphi. The Delphi was conducted across three rounds with the first seeking free-text suggestions from the panel. These were combined with risk factors featuring in a systematic review of common perioperative risk tools [4]. All suggestions were then voted on by the panel across two further rounds to gain consensus. *N*= number of risk factors gained from each source. Our final consensus list of risk factors is available in **supplementary material.**

Figure 2: Simplified process map of a patient referred for elective vascular surgery at our institution. *Blue* boxes highlight preoperative care, *green* intraoperative care, *orange* postoperative care. Boxes indicate processes, diamonds indicate decision points. Dashed lines indicate boundary between community and hospital. Stakeholders (e.g. Anaesthetist) are shown in bold text. Lower panel highlights more granular view of numbered processes including revisions to initial diagram following discussions with a steering group of perioperative professionals. Fully granular maps for elective and emergency pathways are available in the supplementary material. *OIR = overnight intensive recovery (level 2 post anaesthesia care unit for high risk patients)*.

Figure 3: Stakeholder diagram: Stakeholder groups identified for representation on a Delphi panel to understand what defines a high-risk surgical patient and impacts on their outcome. All groups were identified by a collaborative process mapping exercise with a steering group of key perioperative professionals. Diagram template adapted from that available at www.iitoolkit.com, used with permission. *ED = Emergency Department, HCA = Health-care assistant, OT = Occupational therapist, PAU = Pre-assessment unit, PACU = Post-anaesthesia care unit*

Figure 4: Delphi structure showing main risk factor groups identified through participant suggestions and the literature. The questionnaire enabled people to vote on three broad domains (Patient, Admission, Organisation) and relevant groups (black text in lozenges). Many of these groups had relevant subgroups (white text). Participants could also vote on individual date elements (variables or their definitions) within each of these. Full list of these are available in the supplementary material.

Figure 5: Representation of the Delphi questionnaires as a dendrogram, demonstrating hierarchical organisation of questions. Colours represent broad domains of statements being voted on by the panel; pertaining to a patient, circumstances of admission, or those pertaining to the organisation caring for them. Final questions that were voted on are represented by terminal leaves. Branch points

For peer review only

represent question structure which can be viewed in the interactive html document within supplementary material. In panel **A**, expanded nodes represent those variables derived from a systematic review of commonly used perioperative risk scores. In panel **B,** expanded nodes represent those that reached criteria for consensus inclusion in a dataset seeking to understand drivers of perioperative risk in older people.

For peer review only

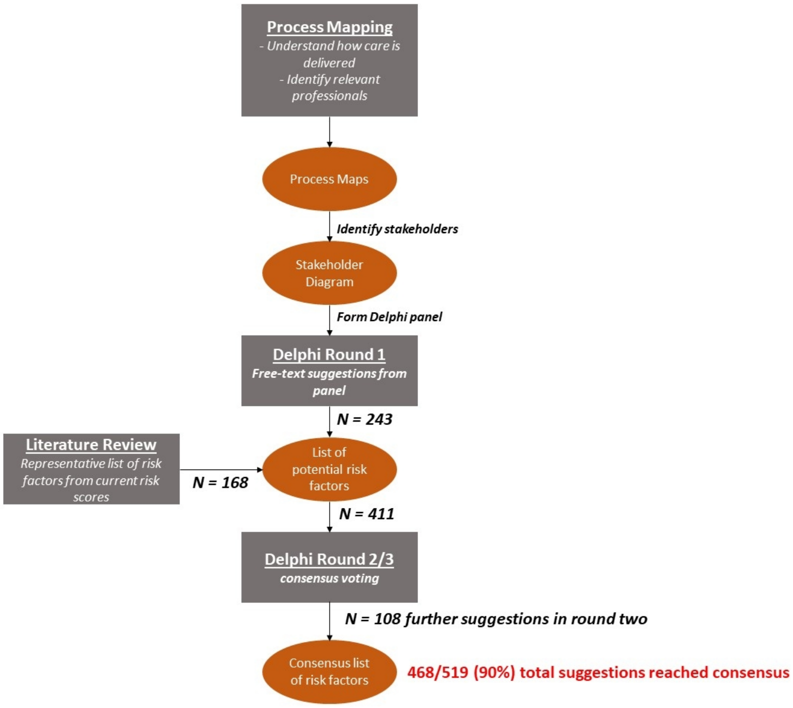

Project stages. Process mapping was performed with a steering group of experienced professionals, with maps iterated between brainstorming interviews. At the conclusion of this stage two outputs were generated – the maps themselves and a stakeholder diagram showing the viewpoints we sought to capture in our Delphi. The Delphi was conducted across three rounds with the first seeking free-text suggestions from the panel. These were combined with risk factors featuring in a systematic review of common perioperative risk tools [4]. All suggestions were then voted on by the panel across two further rounds to gain consensus. N= number of risk factors gained from each source. Our final consensus list of risk factors is available in supplementary material.

355x266mm (96 x 96 DPI)

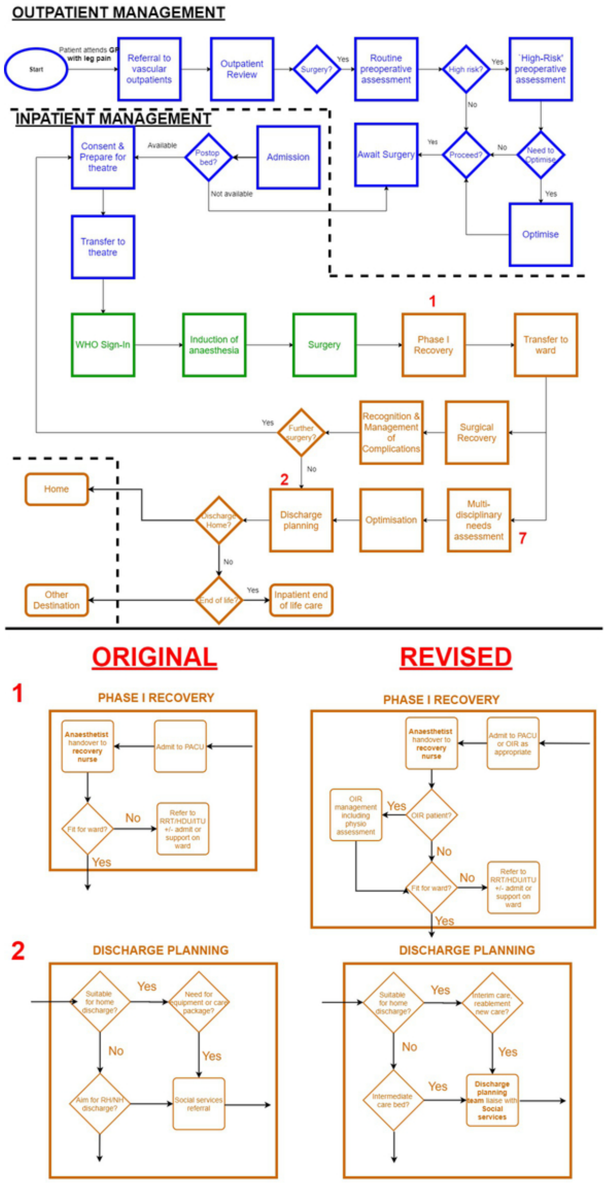

Simplified process map of a patient referred for elective vascular surgery at our institution. Blue boxes highlight preoperative care, green intraoperative care, orange postoperative care. Boxes indicate processes, diamonds indicate decision points. Dashed lines indicate boundary between community and hospital. Stakeholders (e.g. Anaesthetist) are shown in bold text. Lower panel highlights more granular view of numbered processes including revisions to initial diagram following discussions with a steering group of perioperative professionals. Fully granular maps for elective and emergency pathways are available in the supplementary material. OIR = overnight intensive recovery (level 2 post anaesthesia care unit for high risk patients).

49x98mm (300 x 300 DPI)

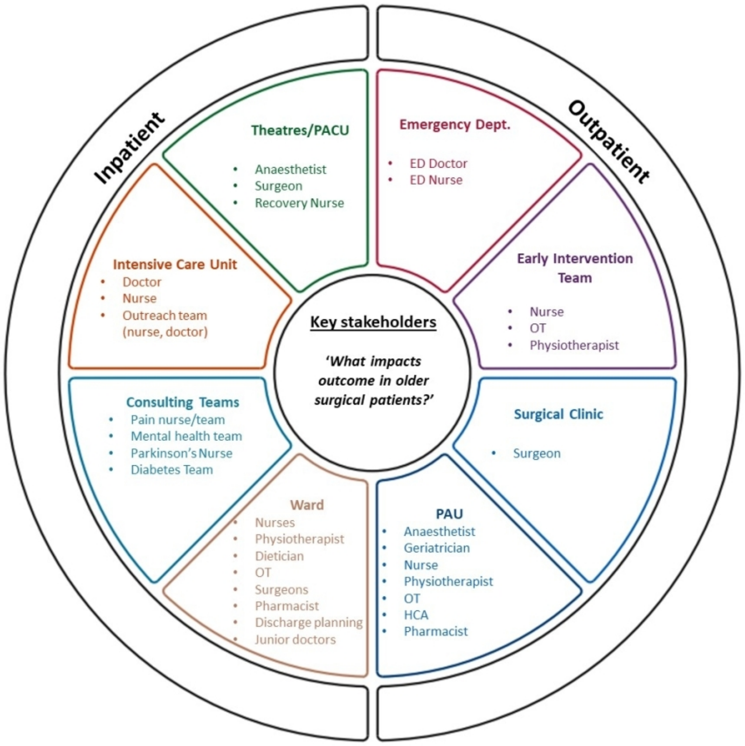

Figure 3: Stakeholder diagram: Stakeholder groups identified for representation on a Delphi panel to understand what defines a high-risk surgical patient and impacts on their outcome. All groups were identified by a collaborative process mapping exercise with a steering group of key perioperative professionals. Diagram template adapted from that available at www.iitoolkit.com, used with permission. ED = Emergency Department, HCA = Health-care assistant, OT = Occupational therapist, PAU = Pre-assessment unit, PACU = Post-anaesthesia care unit

355x266mm (96 x 96 DPI)

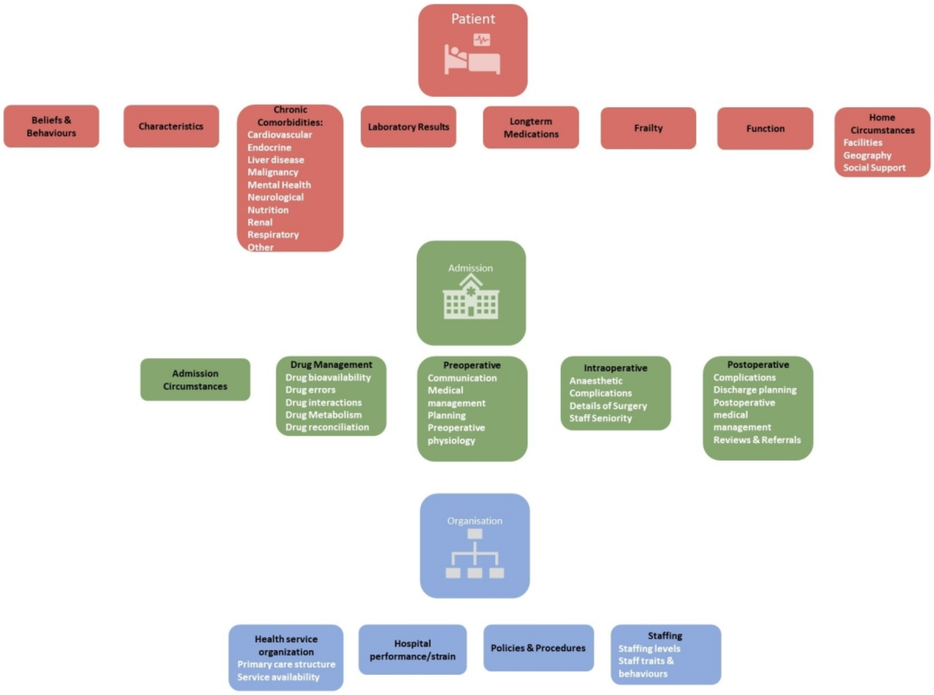

Figure 4: Delphi structure showing main risk factor groups identified through participant suggestions and the literature. The questionnaire enabled people to vote on three broad domains (Patient, Admission, Organisation) and relevant groups (black text in lozenges). Many of these groups had relevant subgroups (white text). Participants could also vote on individual date elements (variables or their definitions) within each of these. Full list of these are available in the supplementary material.

474x339mm (96 x 96 DPI)

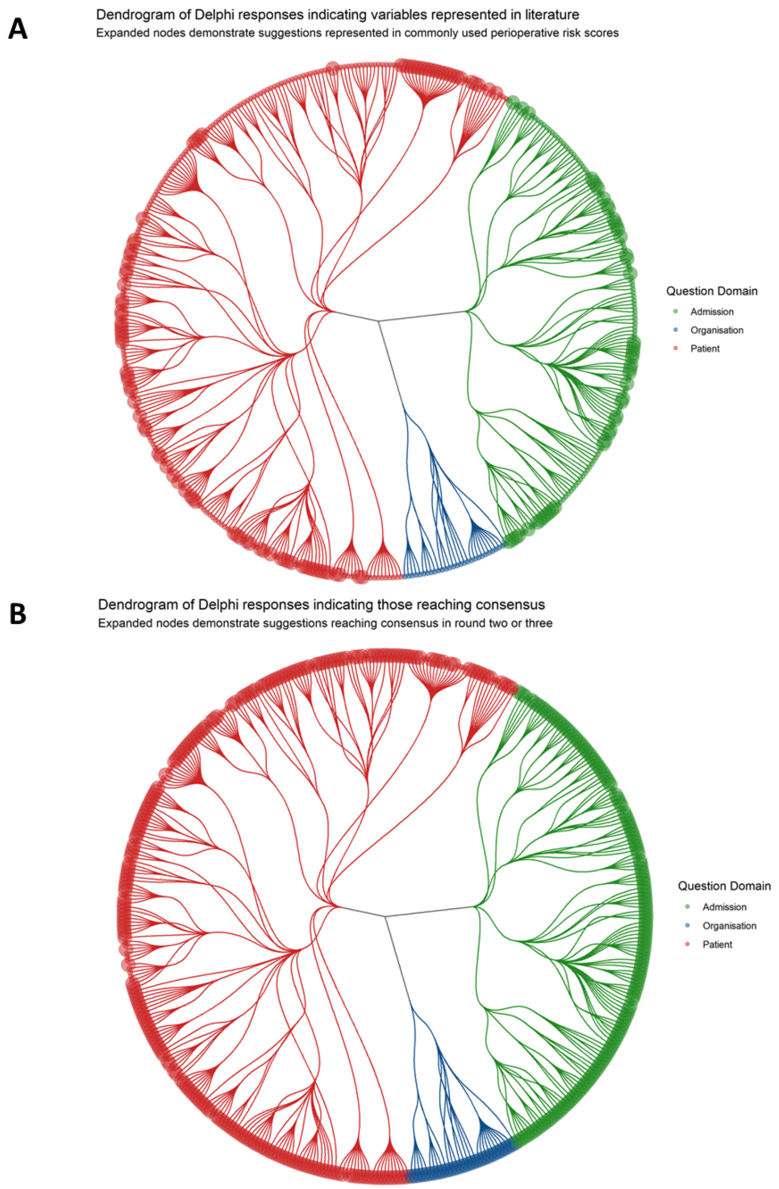

Figure 5: Representation of the Delphi questionnaires as a dendrogram, demonstrating hierarchical organisation of questions. Colours represent broad domains of statements being voted on by the panel; pertaining to a patient, circumstances of admission, or those pertaining to the organisation caring for them. Final questions that were voted on are represented by terminal leaves. Branch points represent question structure which can be viewed in the interactive html document within supplementary material. In panel A, expanded nodes represent those variables derived from a systematic review of commonly used perioperative risk scores. In panel B, expanded nodes represent those that reached criteria for consensus inclusion in a dataset seeking to understand drivers of perioperative risk in older people.

266x355mm (96 x 96 DPI)

Can process mapping and a multi-site Delphi of perioperative professionals inform our understanding of system-wide factors

Stubbs DJ, Bashford T, Nourallah B, Gilder FJ, Ercole A, Levy N, Clarkson PJ

Supplementary Spreadsheet file

This file contains a complete record of all questions asked of the Delphi participants. Details of what is contair understanding of questions you should also refer to the Supplementary html file which contains an interactive

Column/Variable descriptions
Domain/Question Group
Consensus…
Features in risk scores
Screened for risk scores
In SR of processes
New suggestion
N, median, IQR, min, max, comments

Notes:

For peer review only

For peer review only - http://bmjopen.bmj.com/site/about/guidelines.xhtml

r complete

This provides details of where the question featured inside the Delphi questionnaire (for full details
Indicates whether the variable reached the threshold for consensus inclusion or exclusion and, if so,
Indicates whether variable featured in commonly used risk scores as identified from ***Moonesinghe e***
Indicates whether the variable was examined in papers describing the development of the above ris
Indicates whether the variable appears in a systematic review of structure and care process quality i
Whether the variable was a new suggestion by the panel and, if so, in which round
Number of responses, median and IQR of responses (on a 5 point likert scale) and free text commen

Certain variables that the panel voted on were definitions or cut-points of concepts that may or may

For peer review only

of structure see supplementary html file and main supplementary word document)
in which round
t al 2013: https://pubs.asahq.org/anesthesiology/article/119/4/959/11583/Risk-Stratification-To
k scores. All new suggestions were compared against these references
indicators from **Chazapis et al 2018 https://doi.org/10.1016/j.bja.2017.10.001**. All newly identified

ts across both rounds 2 and 3

/ not have featured in earlier rounds or been present in the risk scores. For instance 'BMI' was repres

For peer review only

ols-for-Predicting-Morbidity

variables were mapped against the suggestions in this paper

sented in risk scores but dichotomised cutpoints of 25/30/35 etc... did not

For peer review only

Name of variable
(Fast) AF
Able to drive
Able to use stairs
Abnormal measurements
Analgesic plan
Access difficulties (e.g. steps)
Accomodation type
Activity
Addison's Disease
Multi modal analgesia
Opioid deprescribing pre-discharge
Opioid dose
Adoption of Sick role
Age
Regional/epidural
Albumin
ALT
Admission circumstances
Admission with Raised ICP
Chest injuries
CPR prior to admission
Delayed presentation
Angina
Ankylosing Spondylitis
Anticoagulants
Disruption to a patient's routine
Antiplatelets
Anxiety
Any diagnosis of CCF
Any diagnosis of PVD
Any form of cancer
Any recent acute illness
Emergency (vs elective) admission
APTT
ASA score
Ascites
AST
Asthma
Availability of services
AVFS (Addenbrookes Vascular Frailty Score)
Bathroom access
Bed Occupancy Rates
Bedbound
Bedroom access
Beliefs & Behaviours
Bilirubin
Bipolar disorder
Blood Product Refusal

For peer review only

Multiple injuries
BMI
BMI25
BMI30
BMI35
BMI40
Body Shape
Bone marrow failure
Bone marrow suppression
Brain tumour
Bronchiectasis
Neck of femur fracture
Sepsis or organ failure on admission
Can the house support single storey living?
Subdural haematoma
Cardiomegaly
Cardiomyopathy
Cardiovascular
Trauma
Care Pathway Documents
CFS > 3
CFS > 4
CFS > 5
BUPA Classification
Characteristics & Demographics
Chemotherapy
By Organ System
Cholesterol
Chronic Dysrhythmia
Chronic open wound
Cirrhosis
CK
Claudication
Coagulopathy
Cognitive Impairment
Community Occupational therapy
Community Physiotherapy
Community/isolation
Comorbidities
Congestive Cardiac Failure
Dutch Surgical Society Score
Continent
COPD
COPD prevents ADLs
Could pay for own care
Indication for surgery (pathology)
CVA
Intraperitoneal, aortic, or intrathoracic operation
Deconditioning
Decreased grip strength

For peer review only

Length of Surgery
Defined by ADLs
Need for stoma formation
Numbers of procedures performed
Orthopaedic v Non-Orthopaedic
Delayed Transfer of Care (DToC) Rates
Peritoneal Soiling
Dental services
Dependency on staff
Depends on cancer site
Depression
Dermatomyositis
Severity of Surgery
Diabetes
Diabetes Control
Diabetes Types
Diagnosis of Dementia
Dialysis
Diet Controlled
Digoxin
Skin incision type (e.g. laparoscopic v laparotomy)
Surgery for Cancer
Distance to hospital
District nursing
Diuretics
Surgical Speciality
DOACs (Direct Oral Anticoagulants)
Drug availability (e.g. anticoagulant reversal agents)
Wound classification (e.g. clean/contaminated)
Delayed Transfer of Care
Discharge Planning
Needing care on discharge
Absence of a specific route of administration (e.g. oral)
Antiemetics & Parkinson's medications
Change in nutritional intake
ECG changes
ED Admission wait times
Edmonton > 10
Electronic Frailty Index
Cytochrome P450 inhibitors/inducers
Empathy
Encephalopathy
Endocrine
Engagement with advice/treatment
Enhanced recovery programs
Enthusiasm and engagement
Episodes of Hypoglycaemia
Drug Bioavailability
Ethnicity
Exercise Tolerance

For peer review only

Exhaustion
Expertise in the care of older people
Facilities
Drug Errors
Family anxieties
Family encouragement
Family/Social Support
FEV1 < 50%
Fitness
Drug Interactions
Fluid intake (cups/day)
Fragile skin
Frailty
Frailty score
Frailty Syndrome: Delirium
Frailty Syndrome: Falls
Frailty Syndrome: Incontinence
Frailty Syndromes
Fruit/veg intake
Function
Gangrene
Drug Management
Genetics
Geography
Drug Metabolism
Glucose
Gout
GP availability
Had [Chemotherapy] within 30 days
Had [Radiotherapy] within 90 days
Haematocrit
Haemoglobin
Has Care Package
Has utilities
Hazards at home
HbA1c
Health Service Organisation
Hearing or visual loss
Hemi/paraplegia
High fat ratio (>25%)
Hip to waist ratio
History of Myocardial Infarction
HIV
Hoarding (of items)
Home Circumstances
Home glucose variability
Home NIPPV
Hospital Performance & Strain
Housebound
Drug Reconcilliation

For peer review only

HTN (Hypertension)
Hypoalbuminemia
Inadvertent administration
Inadvertent omission
Immunosuppressants
Immunosuppressed
Impaired LV function
Lack of an alternative medication
Lack of renal dose adjustment
Methotrexate interactions
Independence
Independent decision maker
Independent with dressing
Independent with eating
Independent with Personal Hygiene
Independent with transfers and walking
Independent, Partially independent, Dependent
Poor absorption
Stress factors
Insulin Dependent
Intensive care occupancy
Timing Errors
Timing of drug reconcilliation (e.g. by pharmacy)
When someone restarts their normal medications
Whether it is performed
Airway complication
Anaesthetic Care
Anaesthetic Seniority
Details of Surgery
Intraoperative
Intraoperative Blood Loss
Ischaemic Changes
Lab Results
Intraoperative CO2 Control
Lack of entertainment
Intraoperative Complications
LBBB
Intraoperative drug errors
Liver disease
Lives alone
Local Authority
Intraoperative Heart rate
Long term effects
Longterm Medications
Loss of body fat
Loss of muscle
Low mood
Lymphocytes
Malignancy
Meals/day

For peer review only

Medical cover
Medication compliance
Mental Health
Mental health services
Metastatic cancer
Intraoperative Hypotension
Midarm circumference
Midcalf circumference
Mild Cognitive Impairment
Mild SOB
Mild/Mod SOB
Moderate SOB
More than 10lb
Intraoperative Myocardial Infarction or CPR
Intraoperative Physiological Control
Night time operating
ODP Seniority
Operator Fatigue
Multiple Sclerosis
Myasthenia Gravis
Out of Hours Operating
n=1 (*N of prescription medications)*
n=2 (N of prescription medications)
n=3
n=4 (N of prescription medications)
n=5
n=7 (N of prescription medications)
Neck diameter
Position on operating list
Preoperative LOS
Principal anaesthetic technique (e.g. GA v RA)
Recovery nurse Seniority
Neurological
Scrub nurse Seniority
Staff Seniority
Non-independent (has decision maker)
Non-independent (no decision maker)
Non-insulin Dependent
NSAIDs
Nucleated RBC
Surgical Seniority
Number of comorbidities
Number of medications
Number of outlying patients
Number of patients cared for
Number of vacant posts
Time-to-surgery
Nursing cover
Nutrition
NYHA III or IV

For peer review only

Obese
Occupational therapy cover
Urgency of surgery
Oedema
On chemotherapy
On Home O2
On Methotrexate
On treatment (for diabetes)
Weekend operating
Delay in necessary review
Escalation plan made
Organisational culture
Inappropriate management
Orthopnea
Osteoarthritis
Osteoporosis
Other
Location and level of care
Overnight stay in ED
Parkinson's Disease
Patient Advocacy
Multidisciplinary working
Patient is a carer
Patient is a vulnerable adult
Patient motivation
Patient's confidence
Patient's view of their own health
Patient's wishes & worries
Performance Status
Peripheral nerve injury
Peripheral oedema
Peripheral Vascular Disease
Number of attachments
Pharmacy cover
Physiotherapy cover
Pituitary Failure
Postoperative care (including analgesia & mobilisation)
Postoperative fluid management
Referrals & Reviews
Platelets
Policies and Procedures
Polymyalgia Rheumatica
Polymyositis
Time to medical review (e.g. for complication)
Poor appetite
Poor Cough
Poor diet
Time to mobilisation
Time to occupational therapist review
Time to pharmacist review (e.g. for medicines reconcilliation)

For peer review only

Anastomotic Breakdown
Blood Transfusion
Cardiac Arrest
Cardiovascular Complications
Constipation
Deep Vein Thrombosis
Delirium
Fall
Flap Failure
Gastrointestinal Complications
GI Bleed
Practice type
Pre-existing delirium
Hypotension
Ileus
Infective Complications
Myocardial Infarction
Need for Postoperative Sedation
New diagnosis of chronic conditions (e.g. Dementia)
Poor Wound Healing
Postoperative Acute Renal Failure
Postoperative Complications
Postoperative Deconditioning
Postoperative Dysrhythmia
Postoperative Pain
Postoperative Pneumonia
Postoperative Pulmonary Complications
Postoperative sepsis
Postoperative ventilation
Pressure Sore
Pulmonary Embolism
Reoperation
Surgical Site Infection
Systemic Inflammatory Response Syndrome (SIRS)
Unplanned ITU Admission
Urinary Tract Infection
An appropriate consent process
Appropriate preoperative ward
DNR status & Escalation plan
How long patients expect to stay in hospital
Multidisciplinary review
Multi-disciplinary working & communication
Patient Expectations
Presence of a telephone advice service
Plan for Drug management
Previous Cardiac Surgery
Previous hospitalisation
Previous ITU admission
Previous PCI
Previous surgery

For peer review only

Primary Care Structure
Planning for care on discharge
PRISMA 7 >/= 3 (*Program of Integration of Services for the maintenanc*[illegible]
Procedures for minimising conflicts between teams
Procurement
Protein intake
Proteinuria
PT
Planning for the postoperative period
Q waves
Quality of Life
Radiotherapy
Raised creatinine
Raised JVP
Rate of decline
Prehabilitation program
Preoperative Care
Preoperative Communication (patients, teams, families)
Renal
Preoperative Fluid Balance
Requires mobility aids
Respiratory
Respiratory muscle weakness (e.g. Motor Neurone Disease)
Respiratory Virus Rates
Response to chemotherapy
Rest pain
Rest SOB
Rheumatoid Arthritis
Rheumatological
Salt consumption
Schizophrenia
Scoliosis
Preoperative Fluid Management
Season
Self-reported malnourishment
Preoperative IV Fluid Use
Seronegative spondyloarthropathy
Severe Aortic Stenosis
Preoperative Medical Care
Sex
Preoperative Planning
Shortness of breath
Sjogrens syndrome
Shared decision making process
Smoking history
Social care
Socioeconomics
Starvation (NBM) time
Specific service availability
Splenomegaly

For peer review only

Therapy plans
Staff traits, skills, behaviours
Staffing
Staffing levels
Stairs/more than one storey with no lift
Starting weight
Understand their role in recovery
Strenuous SOB
Understanding of family expectations
Use of a Risk Tool
Substance misuse (incl. alcohol)
Supplement requirement
Surgery cancellation rates
Use of the WHO Checklist
Able to follow commands preoperatively
Potassium
Preoperative acid base status (e.g. pH, bicarbonate)
Symptoms that limit eating
Preoperative Acute Renal impairment
Systemic Lupus Erythematosus
Systemic Sclerosis
T wave inversion
Taking GLP1 analogue
Taking metformin
Taking SGLT2
Tertiary admission/transfer
Their General Health
Their GP Practice type (e.g. practice type)
Preoperative Creatinine
Thrombocytopenia
Preoperative CVS impairment
Preoperative Delirium
Preoperative Electrolytes
Preoperative GCS
Timed get up and go
Timeframe (of weight loss)
Preoperative Hypotension
Preoperative inotrope requirement
Preoperative Neurological impairment
Training levels
Transferrin
Transthyretin
Preoperative O2 Saturations
Triceps skinfold thickness
Triglycerides
Type I v Type II
Type of accomodation (e.g. flat)
Ulceration
Preoperative PaCO2
Preoperative PaO2

For peer review only

Understanding of their condition
Understanding of their situation
Unintentional *(weight loss)*
Preoperative Physiology
Preoperative pneumonia
Preoperative Renal impairment
Preoperative Respiratory Failure
Preoperative Temperature
Varices
Vasculitis
VC < 60%
Visited in hospital
Ward moves/handovers
Warfarin
WCC
Preoperative Urea
Weight
Weight loss
Preoperative Urine output
Preoperative ventilation
Sodium

For peer review only

Domain	Question Group	Consensus (any round)?
Patient	Comorbidities	1
Patient	Function	0
Patient	Function	1
Patient	Comorbidities	1
Admission	Analgesic Plan	1
Patient	Home Circumstances	1
Patient	Home Circumstances	1
Patient	Function	1
Patient	Comorbidities	1
Admission	Analgesic Plan	1
Admission	Analgesic Plan	1
Admission	Analgesic Plan	1
Patient	Beliefs & Behaviours	1
Patient	Characteristics	1
Admission	Analgesic Plan	1
Patient	Lab Results	1
Patient	Lab Results	1
Admission	Circumstances	1
Admission	Circumstances	1
Admission	Circumstances	1
Admission	Circumstances	1
Admission	Circumstances	1
Patient	Comorbidities	1
Patient	Comorbidities	0
Patient	Longterm medications	1
Admission	Circumstances	0
Patient	Longterm medications	1
Patient	Comorbidities	1
Patient	Comorbidities	1
Patient	Comorbidities	1
Patient	Comorbidities	0
Patient	Comorbidities	1
Admission	Circumstances	1
Patient	Lab Results	1
Patient	Characteristics	1
Patient	Comorbidities	1
Patient	Lab Results	1
Patient	Comorbidities	1
Organisation	Health Services Organisation	1
Patient	Frailty	0
Patient	Home Circumstances	1
Organisation	Hospital Performance	1
Patient	Home Circumstances	1
Patient	Home Circumstances	1
Patient	Beliefs & Behaviours	0
Patient	Lab Results	1
Patient	Comorbidities	1
Patient	Beliefs & Behaviours	1

For peer review only

Admission	Circumstances	1
Patient	Comorbidities	1
Patient	Comorbidities	0
Patient	Comorbidities	0
Patient	Comorbidities	0
Patient	Comorbidities	1
Patient	Comorbidities	0
Patient	Comorbidities	1
Patient	Comorbidities	1
Patient	Comorbidities	1
Patient	Comorbidities	1
Admission	Circumstances	1
Admission	Circumstances	1
Patient	Home Circumstances	1
Admission	Circumstances	1
Patient	Comorbidities	1
Patient	Comorbidities	1
Patient	Comorbidities	1
Admission	Circumstances	1
Organisation	Policies and Procedures	1
Patient	Frailty	1
Patient	Frailty	1
Patient	Frailty	1
Admission	Details of Surgery	0
Patient	Characteristics	0
Patient	Chemotherapy	1
Admission	Details of Surgery	1
Patient	Lab Results	0
Patient	Comorbidities	1
Patient	Comorbidities	1
Patient	Lab Results	1
Patient	Lab Results	0
Patient	Comorbidities	1
Patient	Comorbidities	1
Patient	Comorbidities	1
Patient	Home Circumstances	1
Patient	Home Circumstances	1
Patient	Home Circumstances	1
Patient	Comorbidities	1
Patient	Comorbidities	1
Admission	Details of Surgery	0
Patient	Function	1
Patient	Comorbidities	1
Patient	Comorbidities	1
Patient	Home Circumstances	0
Admission	Details of Surgery	1
Patient	Comorbidities	1
Admission	Details of Surgery	1
Patient	Frailty	1
Patient	Frailty	1

For peer review only

Admission	Details of Surgery	1
Patient	Function	1
Admission	Details of Surgery	1
Admission	Details of Surgery	1
Admission	Details of Surgery	0
Organisation	Hospital Performance	1
Admission	Details of Surgery	1
Patient	Home Circumstances	0
Patient	Beliefs & Behaviours	1
Patient	Comorbidities	0
Patient	Comorbidities	1
Patient	Comorbidities	0
Admission	Details of Surgery	1
Patient	Comorbidities	1
Patient	Comorbidities	1
Patient	Comorbidities	1
Patient	Comorbidities	1
Patient	Comorbidities	1
Patient	Comorbidities	1
Patient	Comorbidities	1
Admission	Details of Surgery	1
Admission	Details of Surgery	1
Patient	Home Circumstances	1
Patient	Home Circumstances	1
Patient	Longterm medications	1
Admission	Details of Surgery	1
Patient	Longterm medications	1
Organisation	Policies and Procedures	1
Admission	Details of Surgery	1
Admission	Discharge Planning	1
Admission	Discharge Planning	1
Admission	Discharge Planning	1
Admission	Drug Management	1
Admission	Drug Management	1
Admission	Drug Management	1
Patient	Comorbidities	1
Organisation	Hospital Performance	1
Patient	Frailty	0
Patient	Frailty	0
Admission	Drug Management	1
Organisation	Staffing	1
Patient	Comorbidities	1
Patient	Comorbidities	1
Patient	Beliefs & Behaviours	1
Organisation	Policies and Procedures	1
Organisation	Staffing	1
Patient	Comorbidities	1
Admission	Drug Management	1
Patient	Characteristics	1
Patient	Comorbidities	1

For peer review only

Patient	Frailty	1
Organisation	Staffing	1
Patient	Home Circumstances	1
Admission	Drug Management	1
Patient	Home Circumstances	1
Patient	Home Circumstances	1
Patient	Home Circumstances	1
Patient	Comorbidities	1
Patient	Function	1
Admission	Drug Management	1
Patient	Comorbidities	1
Patient	Comorbidities	1
Patient	Frailty	1
Patient	Frailty	1
Patient	Frailty	1
Patient	Frailty	1
Patient	Frailty	0
Patient	Frailty	1
Patient	Comorbidities	1
Patient	Function	1
Patient	Comorbidities	1
Admission	Drug Management	1
Patient	Characteristics	1
Patient	Home Circumstances	1
Admission	Drug Management	1
Patient	Lab Results	1
Patient	Comorbidities	0
Patient	Home Circumstances	1
Patient	Comorbidities	1
Patient	Comorbidities	1
Patient	Lab Results	1
Patient	Lab Results	1
Patient	Function	1
Patient	Home Circumstances	1
Patient	Home Circumstances	1
Patient	Comorbidities	1
Organisation	Health Services Organisation	1
Patient	Characteristics	1
Patient	Comorbidities	1
Patient	Comorbidities	0
Patient	Comorbidities	0
Patient	Comorbidities	1
Patient	Comorbidities	1
Patient	Home Circumstances	1
Patient	Home Circumstances	1
Patient	Comorbidities	1
Patient	Comorbidities	1
Organisation	Hospital Performance	0
Patient	Function	1
Admission	Drug Management	1

For peer review only

Patient	Comorbidities	1
Patient	Comorbidities	1
Admission	Drug Management	1
Admission	Drug Management	1
Patient	Longterm medications	1
Patient	Comorbidities	1
Patient	Comorbidities	1
Admission	Drug Management	1
Admission	Drug Management	1
Admission	Drug Management	1
Patient	Function	1
Patient	Home Circumstances	1
Patient	Function	1
Patient	Function	1
Patient	Function	1
Patient	Function	1
Patient	Function	1
Admission	Drug Management	1
Admission	Drug Management	1
Patient	Comorbidities	1
Organisation	Hospital Performance	1
Admission	Drug Management	1
Admission	Drug Management	1
Admission	Drug Management	1
Admission	Drug Management	1
Admission	Intraoperative	1
Admission	Intraoperative	1
Admission	Intraoperative	1
Admission	Intraoperative	1
Admission	Intraoperative	1
Admission	Intraoperative	1
Patient	Comorbidities	1
Patient	Lab Results	1
Admission	Intraoperative	1
Patient	Home Circumstances	1
Admission	Intraoperative	1
Patient	Comorbidities	1
Admission	Intraoperative	1
Patient	Comorbidities	1
Patient	Home Circumstances	1
Patient	Home Circumstances	0
Admission	Intraoperative	1
Patient	Comorbidities	1
Patient	Longterm medications	1
Patient	Comorbidities	1
Patient	Frailty	1
Patient	Comorbidities	1
Patient	Lab Results	1
Patient	Comorbidities	1
Patient	Comorbidities	1

For peer review only

Organisation	Staffing	1
Patient	Beliefs & Behaviours	1
Patient	Comorbidities	1
Patient	Home Circumstances	1
Patient	Comorbidities	1
Admission	Intraoperative	1
Patient	Comorbidities	0
Patient	Comorbidities	0
Patient	Comorbidities	1
Patient	Comorbidities	1
Patient	Comorbidities	1
Patient	Comorbidities	1
Patient	Comorbidities	1
Admission	Intraoperative	1
Admission	Intraoperative	1
Admission	Intraoperative	1
Admission	Intraoperative	0
Admission	Intraoperative	1
Patient	Comorbidities	1
Patient	Comorbidities	1
Admission	Intraoperative	1
Patient	Longterm medications	0
Patient	Longterm medications	0
Patient	Longterm medications	0
Patient	Longterm medications	1
Patient	Longterm medications	1
Patient	Longterm medications	1
Patient	Comorbidities	0
Admission	Intraoperative	1
Admission	Intraoperative	1
Admission	Intraoperative	1
Admission	Intraoperative	1
Patient	Comorbidities	1
Admission	Intraoperative	1
Admission	Intraoperative	1
Patient	Home Circumstances	1
Patient	Home Circumstances	1
Patient	Comorbidities	1
Patient	Longterm medications	1
Patient	Lab Results	0
Admission	Intraoperative	1
Patient	Comorbidities	1
Patient	Longterm medications	1
Organisation	Hospital Performance	1
Organisation	Health Services Organisation	1
Organisation	Hospital Performance	1
Admission	Intraoperative	1
Organisation	Staffing	1
Patient	Comorbidities	1
Patient	Comorbidities	1

For peer review only

Patient	Comorbidities	1
Organisation	Staffing	1
Admission	Intraoperative	1
Patient	Comorbidities	1
Patient	Comorbidities	1
Patient	Comorbidities	1
Patient	Comorbidities	1
Patient	Comorbidities	1
Admission	Intraoperative	1
Admission	Postoperative	1
Admission	Postoperative	1
Organisation	Staffing	1
Admission	Postoperative	1
Patient	Comorbidities	1
Patient	Comorbidities	0
Patient	Comorbidities	0
Patient	Comorbidities	1
Admission	Postoperative	1
Organisation	Hospital Performance	1
Patient	Comorbidities	1
Patient	Home Circumstances	1
Admission	Postoperative	1
Patient	Home Circumstances	1
Patient	Home Circumstances	1
Patient	Beliefs & Behaviours	1
Patient	Beliefs & Behaviours	1
Patient	Beliefs & Behaviours	1
Patient	Beliefs & Behaviours	1
Patient	Function	1
Patient	Comorbidities	1
Patient	Comorbidities	1
Patient	Comorbidities	1
Admission	Postoperative	1
Organisation	Staffing	1
Organisation	Staffing	1
Patient	Comorbidities	1
Admission	Postoperative	1
Admission	Postoperative	1
Admission	Postoperative	1
Patient	Lab Results	1
Organisation	Policies and Procedures	1
Patient	Comorbidities	0
Patient	Comorbidities	1
Admission	Postoperative	1
Patient	Comorbidities	0
Patient	Comorbidities	1
Patient	Comorbidities	1
Admission	Postoperative	1
Admission	Postoperative	1
Admission	Postoperative	1

For peer review only

Admission	Postoperative Complications	1
Admission	Postoperative Complications	1
Admission	Postoperative Complications	1
Admission	Postoperative Complications	1
Admission	Postoperative Complications	1
Admission	Postoperative Complications	1
Admission	Postoperative Complications	1
Admission	Postoperative Complications	1
Admission	Postoperative Complications	1
Admission	Postoperative Complications	1
Admission	Postoperative Complications	1
Organisation	Health Services Organisation	1
Patient	Comorbidities	1
Admission	Postoperative Complications	1
Admission	Postoperative Complications	1
Admission	Postoperative Complications	1
Admission	Postoperative Complications	1
Admission	Postoperative Complications	0
Admission	Postoperative Complications	1
Admission	Postoperative Complications	1
Admission	Postoperative Complications	1
Admission	Postoperative Complications	1
Admission	Postoperative Complications	1
Admission	Postoperative Complications	1
Admission	Postoperative Complications	1
Admission	Postoperative Complications	1
Admission	Postoperative Complications	1
Admission	Postoperative Complications	1
Admission	Postoperative Complications	1
Admission	Postoperative Complications	1
Admission	Postoperative Complications	1
Admission	Postoperative Complications	1
Admission	Postoperative Complications	1
Admission	Postoperative Complications	1
Admission	Postoperative Complications	1
Admission	Postoperative Complications	1
Admission	Preoperative	1
Admission	Preoperative	1
Admission	Preoperative	1
Admission	Preoperative	1
Admission	Preoperative	1
Admission	Preoperative	1
Admission	Preoperative	1
Organisation	Health Services Organisation	1
Admission	Preoperative	1
Patient	Comorbidities	1
Patient	Comorbidities	1
Patient	Comorbidities	1
Patient	Comorbidities	1
Patient	Comorbidities	0

For peer review only

Organisation	Health Services Organisation	1
Admission	Preoperative	1
Patient	Frailty	0
Organisation	Policies and Procedures	1
Organisation	Policies and Procedures	1
Patient	Comorbidities	1
Patient	Comorbidities	1
Patient	Lab Results	1
Admission	Preoperative	1
Patient	Comorbidities	1
Patient	Characteristics	1
Patient	Comorbidities	1
Patient	Comorbidities	1
Patient	Comorbidities	1
Patient	Frailty	1
Admission	Preoperative	1
Admission	Preoperative	1
Admission	Preoperative	1
Patient	Comorbidities	1
Admission	Preoperative	1
Patient	Function	1
Patient	Comorbidities	1
Patient	Comorbidities	1
Organisation	Hospital Performance	1
Patient	Comorbidities	0
Patient	Comorbidities	1
Patient	Comorbidities	1
Patient	Comorbidities	1
Patient	Comorbidities	1
Patient	Comorbidities	1
Patient	Comorbidities	1
Patient	Comorbidities	1
Admission	Preoperative	1
Organisation	Hospital Performance	1
Patient	Comorbidities	1
Admission	Preoperative	1
Patient	Comorbidities	1
Patient	Comorbidities	1
Admission	Preoperative	1
Patient	Characteristics	0
Admission	Preoperative	1
Patient	Comorbidities	1
Patient	Comorbidities	1
Admission	Preoperative	1
Patient	Characteristics	1
Patient	Home Circumstances	1
Patient	Home Circumstances	1
Admission	Preoperative	1
Patient	Home Circumstances	1
Patient	Comorbidities	1

For peer review only

Admission	Preoperative	1
Organisation	Staffing	1
Organisation	Staffing	1
Organisation	Staffing	1
Patient	Home Circumstances	1
Patient	Comorbidities	1
Admission	Preoperative	1
Patient	Comorbidities	1
Admission	Preoperative	1
Admission	Preoperative	1
Patient	Comorbidities	1
Patient	Comorbidities	1
Organisation	Hospital Performance	1
Admission	Preoperative	1
Admission	Preoperative Physiology	1
Admission	Preoperative Physiology	1
Admission	Preoperative Physiology	1
Patient	Comorbidities	1
Admission	Preoperative Physiology	1
Patient	Comorbidities	1
Patient	Comorbidities	1
Patient	Comorbidities	1
Patient	Comorbidities	0
Patient	Comorbidities	1
Patient	Comorbidities	1
Organisation	Health Services Organisation	1
Patient	Characteristics	1
Patient	Home Circumstances	1
Admission	Preoperative Physiology	1
Patient	Lab Results	1
Admission	Preoperative Physiology	1
Admission	Preoperative Physiology	1
Admission	Preoperative Physiology	1
Admission	Preoperative Physiology	1
Patient	Frailty	0
Patient	Comorbidities	1
Admission	Preoperative Physiology	1
Admission	Preoperative Physiology	1
Admission	Preoperative Physiology	1
Organisation	Staffing	1
Patient	Lab Results	0
Patient	Lab Results	0
Admission	Preoperative Physiology	1
Patient	Comorbidities	0
Patient	Lab Results	0
Patient	Comorbidities	1
Patient	Home Circumstances	1
Patient	Comorbidities	1
Admission	Preoperative Physiology	1
Admission	Preoperative Physiology	1

For peer review only

Patient	Beliefs & Behaviours	1
Patient	Beliefs & Behaviours	1
Patient	Comorbidities	1
Admission	Preoperative Physiology	1
Admission	Preoperative Physiology	1
Admission	Preoperative Physiology	1
Admission	Preoperative Physiology	1
Admission	Preoperative Physiology	1
Patient	Comorbidities	1
Patient	Comorbidities	1
Patient	Comorbidities	1
Patient	Home Circumstances	1
Admission	Postoperative	1
Patient	Longterm medications	1
Patient	Lab Results	1
Admission	Preoperative Physiology	1
Patient	Comorbidities	1
Patient	Comorbidities	1
Admission	Preoperative Physiology	1
Admission	Preoperative Physiology	1
Admission	Preoperative Physiology	1

For peer review only

Consensus Include (Round 2)	Consensus Include (Round 3)
0	1
0	0
0	1
1	0
1	0
1	0
1	0
1	0
0	1
1	0
1	0
1	0
1	0
1	0
1	0
1	0
1	0
1	0
1	0
1	0
1	0
1	0
1	0
0	0
1	0
0	1
0	1
1	0
1	0
1	0
0	0
1	0
1	0
0	1
1	0
1	0
1	0
1	0
1	0
0	0
0	1
1	0
1	0
0	1
0	0
1	0
1	0
0	1

For peer review only

1	0
1	0
0	0
0	0
0	0
0	1
0	0
0	1
0	1
1	0
1	0
0	1
0	1
0	1
0	1
1	0
1	0
1	0
1	0
1	0
0	1
0	1
0	1
0	0
0	0
1	0
1	0
0	0
1	0
1	0
1	0
0	0
0	1
1	0
1	0
0	1
0	1
0	1
1	0
1	0
0	0
1	0
1	0
1	0
0	0
1	0
1	0
1	0
0	1
1	0

For peer review only

1	0
1	0
1	0
1	0
0	0
1	0
1	0
0	0
1	0
0	0
1	0
0	1
1	0
1	0
1	0
1	0
1	0
1	0
0	1
1	0
1	0
1	0
0	1
0	1
1	0
0	1
1	0
1	0
1	0
1	0
1	0
1	0
1	0
1	0
0	1
0	1
0	1
0	0
0	0
0	1
1	0
0	1
1	0
1	0
1	0
0	1
0	1
1	0
0	1
0	1

For peer review only

1	0
1	0
1	0
1	0
1	0
1	0
1	0
1	0
1	0
1	0
1	0
1	0
1	0
1	0
0	1
0	1
0	0
1	0
1	0
1	0
1	0
1	0
1	0
1	0
1	0
1	0
0	0
0	1
1	0
0	1
1	0
1	0
1	0
1	0
1	0
1	0
1	0
1	0
1	0
0	1
0	0
1	0
0	1
0	1
1	0
0	1
0	1
0	0
1	0
1	0

For peer review only

1	0
0	1
1	0
1	0
0	1
1	0
1	0
1	0
1	0
0	1
1	0
1	0
1	0
1	0
1	0
1	0
0	1
1	0
0	1
1	0
0	1
0	1
1	0
1	0
1	0
1	0
1	0
1	0
1	0
1	0
1	0
0	1
1	0
1	0
0	1
1	0
0	1
1	0
1	0
1	0
0	0
1	0
0	1
1	0
1	0
0	1
1	0
1	0
1	0
1	0

For peer review only

1	0
1	0
1	0
0	1
1	0
1	0
0	0
0	0
1	0
1	0
1	0
1	0
1	0
1	0
1	0
1	0
0	0
1	0
1	0
1	0
1	0
0	0
0	0
0	0
0	1
0	1
0	1
0	0
0	1
1	0
1	0
0	1
1	0
0	1
1	0
1	0
0	1
1	0
1	0
0	0
1	0
1	0
1	0
0	1
1	0
1	0
1	0
1	0
1	0
1	0

For peer review only

1	0
1	0
1	0
1	0
1	0
0	1
0	1
1	0
0	1
1	0
1	0
0	1
1	0
0	1
0	0
0	0
1	0
1	0
0	1
1	0
1	0
1	0
1	0
1	0
1	0
1	0
1	0
1	0
1	0
1	0
1	0
1	0
1	0
1	0
1	0
0	1
1	0
1	0
1	0
1	0
1	0
0	0
0	1
1	0
0	0
0	1
1	0
1	0
1	0
1	0

For peer review only

1	0
1	0
1	0
1	0
1	0
1	0
1	0
1	0
1	0
1	0
1	0
1	0
1	0
1	0
1	0
1	0
1	0
0	0
0	1
1	0
1	0
1	0
1	0
0	1
1	0
1	0
1	0
1	0
1	0
1	0
1	0
1	0
1	0
1	0
1	0
1	0
1	0
1	0
1	0
1	0
1	0
1	0
1	0
0	1
1	0
1	0
1	0
0	1
0	1
0	0

For peer review only

For peer review only - http://bmjopen.bmj.com/site/about/guidelines.xhtml

For peer review only

1	0
1	0
0	0
1	0
0	1
1	0
0	1
1	0
1	0
1	0
1	0
1	0
1	0
1	0
1	0
0	1
1	0
1	0
1	0
1	0
1	0
1	0
0	1
1	0
0	0
1	0
1	0
0	1
1	0
1	0
1	0
0	1
1	0
0	1
1	0
0	1
0	1
1	0
1	0
0	0
1	0
1	0
0	1
1	0
1	0
0	1
1	0
1	0
1	0
0	1

1	0
1	0
1	0
1	0
0	1
0	1
0	1
0	0
1	0
1	0
1	0
0	1
0	1
1	0
1	0
1	0
1	0
1	0
1	0
0	1
0	1
0	1
0	0
0	1
0	1
0	1
1	0
1	0
1	0
0	1
1	0
1	0
1	0
1	0
0	0
0	1
1	0
1	0
1	0
1	0
0	0
0	0
1	0
0	0
0	0
0	1
1	0
0	1
1	0
1	0

For peer review only

1	0
0	1
0	1
1	0
1	0
1	0
1	0
1	0
1	0
0	1
1	0
1	0
1	0
1	0
1	0
1	0
1	0
1	0
1	0
1	0
1	0

For peer review only

Consensus Exclude (Round 2)	Consensus Exclude (Round 3)	Feature in Risk Scores?
0	0	0
0	0	0
0	0	0
0	0	0
0	0	0
0	0	0
0	0	0
0	0	0
0	0	0
0	0	0
0	0	0
0	0	0
0	0	0
0	0	1
0	0	0
0	0	1
0	0	1
0	0	1
0	0	1
0	0	0
0	0	1
0	0	0
0	0	1
0	0	0
0	0	0
0	0	0
0	0	0
0	0	0
0	0	1
0	0	1
0	0	0
0	0	1
0	0	1
0	0	1
0	0	1
0	0	1
0	0	1
0	0	1
0	0	0
0	0	0
0	0	0
0	0	0
0	0	0
0	0	0
0	0	0
0	0	1
0	0	0
0	0	0

For peer review only - http://bmjopen.bmj.com/site/about/guidelines.xhtml

0	0	0
0	0	1
0	0	0
0	0	0
0	0	0
0	0	0
0	0	0
0	0	0
0	0	0
0	0	1
0	0	0
0	0	0
0	0	0
0	0	0
0	0	0
0	0	1
0	0	1
0	0	1
0	0	0
0	0	0
0	0	0
0	0	0
0	0	0
0	0	1
0	0	0
0	0	1
0	0	0
0	0	1
0	0	1
0	0	1
0	0	1
0	0	1
0	0	0
0	0	1
0	0	1
0	0	0
0	0	0
0	0	0
0	0	0
0	0	1
0	0	1
0	0	0
0	0	1
0	0	0
0	0	0
0	0	0
0	0	1
0	0	1
0	0	0
0	0	1

For peer review only

0	0	1
0	0	1
0	0	0
0	0	1
0	0	0
0	0	0
0	0	1
0	0	0
		0
0	0	0
0	0	0
0	0	0
0	0	0
0	0	1
0	0	0
0	0	0
0	0	1
0	0	1
0	0	0
0	0	1
0	0	1
0	0	1
0	0	0
0	0	0
0	0	1
0	0	0
0	0	0
0	0	0
0	0	1
0	0	0
0	0	0
0	0	0
0	0	0
0	0	0
0	0	0
0	0	1
0	0	0
0	0	0
0	0	0
0	0	0
0	0	0
0	0	0
0	0	0
0	0	0
0	0	0
0	0	0
0	0	0
0	0	0
0	0	0
0	0	0

For peer review only

0	0	1
0	0	0
0	0	0
0	0	0
0	0	0
0	0	0
0	0	1
0	0	1
0	0	0
0	0	0
0	0	1
0	0	0
0	0	1
0	0	0
0	0	0
0	0	0
0	0	0
0	0	0
0	0	1
0	0	0
0	0	1
0	0	0
0	0	0
0	0	0
0	0	0
0	0	1
0	0	0
0	0	0
0	0	1
0	0	1
0	0	1
0	0	1
0	0	0
0	0	0
0	0	0
0	0	0
0	0	0
0	0	0
0	0	1
0	0	0
0	0	0
0	0	1
0	0	1
0	0	0
0	0	0
0	0	0
0	0	0
0	0	0
0	0	0
0	0	0

For peer review only

0	0	1
0	0	1
0	0	0
0	0	0
0	0	0
0	0	0
0	0	1
0	0	0
0	0	0
0	0	0
0	0	0
0	0	0
0	0	0
0	0	0
0	0	0
0	0	0
0	0	0
0	0	0
0	0	0
0	0	0
0	0	0
0	0	0
0	0	0
0	0	0
0	0	0
0	0	1
0	0	0
0	0	0
0	0	0
0	0	0
0	0	1
0	0	0
0	0	1
0	0	0
0	0	0
0	0	0
0	0	0
0	0	0
0	0	1
0	0	0
0	0	0
0	0	1
0	0	0
0	0	1
0	0	0
0	0	1
0	0	1
0	0	1
0	0	1
0	0	1

For peer review only

0	0	0
		0
0	0	0
0	0	0
0	0	1
0	0	0
0	0	1
0	0	1
0	0	1
0	0	0
0	0	0
0	0	0
0	0	1
0	0	1
0	0	0
0	0	0
0	0	0
0	0	0
0	0	0
0	0	0
0	0	0
0	0	0
0	0	0
0	0	1
0	0	0
0	0	1
0	0	0
0	0	0
0	0	0
0	0	1
0	0	1
0	0	0
0	0	0
0	0	0
0	0	0
0	0	0
0	0	0
0	0	0
0	0	0
0	0	1
0	0	1
0	0	0
0	0	1
0	0	0
0	0	0
0	0	0
0	0	1
0	0	0
0	0	1
0	0	1

For peer review only

0	0	0
0	0	0
0	0	1
0	0	0
0	0	1
0	0	0
0	0	0
0	0	0
0	0	0
0	0	0
0	0	0
0	0	0
0	0	0
0	0	0
0	0	0
0	0	0
0	0	0
0	0	0
0	0	0
0	0	0
0	0	0
0	0	0
0	0	0
0	0	0
		0
		0
0	0	0
0	0	0
0	0	0
0	0	1
0	0	1
0	0	1
0	0	0
0	0	0
0	0	0
0	0	0
0	0	0
0	0	0
0	0	0
0	0	1
0	0	0
0	0	0
0	0	0
0	0	0
0	0	1
0	0	0
0	0	1
0	0	0
0	0	0
0	0	0

For peer review only

0	0	0
0	0	1
0	0	1
0	0	1
0	0	0
0	0	1
0	0	1
0	0	1
0	0	1
0	0	0
0	0	1
0	0	0
0	0	0
0	0	1
0	0	0
0	0	0
0	0	1
0	0	0
0	0	0
0	0	0
0	0	1
0	0	0
0	0	1
0	0	0
0	0	0
0	0	0
0	0	1
0	0	0
0	0	1
0	0	0
0	0	1
0	0	1
0	0	0
0	0	1
0	0	1
0	0	1
0	0	0
0	0	0
0	0	0
0	0	0
0	0	0
0	0	0
0	0	0
0	0	0
0	0	0
0	0	1
0	0	1
0	0	0
0	0	1
0	0	0

For peer review only

0	0	0
0	0	0
0	0	0
0	0	0
0	0	0
0	0	1
0	0	0
0	0	1
0	0	0
0	0	1
0	0	0
0	0	1
0	0	1
0	0	1
0	0	1
0	0	0
0	0	0
0	0	0
0	0	1
0	0	0
0	0	0
0	0	1
0	0	0
0	0	0
0	0	0
0	0	1
0	0	0
0	0	0
0	0	1
0	0	1
0	0	0
0	0	0
0	0	0
0	0	0
0	0	1
0	0	0
0	0	0
0	0	1
0	0	1
0	0	1
0	0	0
0	0	0
0	0	0
0	0	0
0	0	1
0	0	0
0	0	0
0	0	0
0	0	0
0	0	0

For peer review only

0	0	0
0	0	0
0	0	0
0	0	0
0	0	0
0	0	1
0	0	0
0	1	0
0	0	0
0	0	0
0	0	0
0	0	0
0	0	0
0	0	0
0	0	0
0	0	1
0	0	1
0	0	1
0	0	1
0	0	0
0	0	0
0	0	0
0	0	0
0	0	0
0	0	0
0	0	0
0	0	0
0	0	1
0	0	0
0	0	0
0	0	0
0	0	0
0	0	1
0	0	0
0	0	0
0	0	1
0	0	1
0	0	0
0	0	0
0	0	1
0	0	1
0	0	0
0	0	1
0	0	1
0	0	0
0	0	0
0	0	0
0	0	0
0	0	1

For peer review only

0	0	0
0	0	0
0	0	0
0	0	0
0	0	1
0	0	0
0	0	0
0	0	1
0	0	1
0	0	0
0	0	1
0	0	0
0	0	0
0	0	1
0	0	1
0	0	1
0	0	0
0	0	1
0	0	1
0	0	1
0	0	1

For peer review only

Screened for Risk Scores?	In SR of processes	New sugge	New sugge	N(round2)	Median(ro	IQR(round2
	0	0	1			
	0	0	1			
1	0	0	1			
	0	0	0	28	4	1
	1	1	0	26	4	1
	0	1	0	26	4	0
	0	1	0	28	4	1
	0	1	0	27	5	1
	0	0	1			
	1	1	0	24	5	1
	0	1	0	25	4	1
	0	1	0	25	4	1
	0	1	0	32	4.5	1
1		0	0	31	5	1
1	1	1	0	24	4	1
1		0	0	25	4	0
		0	0	21	4	1
	0	0	0	27	4	1
	0	0	0	25	5	1
	0	1	0	24	5	1
	0	0	0	27	5	0
	0	1	0	27	5	1
1		0	0	29	5	1
	0	0	1			
	0	1	0	26	5	1
	0	1	0	27	3	1
	0	0	1			
	0	1	0	27	4	0
		0	0	26	4	1
		0	0	24	4	1
1	0	0	1			
1		0	0	29	5	1
1	1	0	0	27	5	1
		0	0	20	4	1.25
1		0	0	25	4	1
		0	0	27	5	1
1		0	0	18	4	1
		0	0	28	4	1
	1	1	0	27	5	1
	0	0	1			
	0	0	1			
	0	1	0	27	5	1
1	0	1	0	27	5	0
	0	0	1			
	0	1	0	33	4	2
		0	0	21	4	1
	0	1	0	26	4	1
	0	1	0	31	4	2

For peer review only

1	0	1	0	24	5	0
1		0	0	28	4	1
	0	0	1			
	0	0	1			
	0	0	1			
	0	0	1			
	0	0	1			
	0	0	1			
	0	0	1			
1		0	0	26	4	1
	0	1	0	27	5	1
	1	0	1			
1	0	0	1			
	0	1	0			
	0	0	1			
1		0	0	27	4	1
		0	0	28	5	1
		0	0	30	5	0
	0	0	0	25	5	1
	1	1	0	25	4	1
	0	1	0			
	0	1	0			
	0	1	0			
1	0	1	0	7	3	1
	0	0	0	31	4	1.5
		0	0	27	4	1
	1	1	0	20	4	0.5
		0	0	23	3	0
1		0	0	28	4	1
1		0	0	28	4	1
1		0	0	27	5	1
		0	0	22	3	1
	0	0	1			
		0	0	26	5	0.75
		0	0	28	5	1
	0	0	1			
	0	0	1			
	0	0	1			
		0	0	30	5	0
1		0	0	28	5	0.25
1	0	0	0	3	3	1
1	0	1	0	27	4	1
		0	0	28	5	1
	0	0	0	27	5	0.5
	0	1	0	27	3	1
	0	1	0	25	5	1
1		0	0	29	5	1
1	0	1	0	16	4	1
	0	1	0	25	4	2
		0	0	26	4	0.75

For peer review only

1	1	0	0	24	5	1
1		0	0	28	4	1
	1	1	0	22	4	0.75
1	0	0	0	24	4	1
	0	1	0	21	3	1
	0	1	0	25	4	1
1	0	0	0	21	5	1
	0	0	1			
	0	1	0	32	4	1
	0	0	1			
1	0	1	0	27	4	0
	0	0	1			
	1	1	0	24	5	0
1	1	0	0	29	5	1
	1	1	0	29	5	1
	1	1	0	28	4	1
1		0	0	27	4	1
1		0	0	27	5	0
	0	1	0	26	3	1
		0	0	24	4	1
1	0	0	0	18	4	1
	1	0	0	22	4.5	1
	0	1	0	24	3	1
	0	0	1			
		0	0	24	4	1
1	1	0	1	24	4	2
	0	1	0	25	5	1
	1	1	0	24	4	1
1	0	0	0	24	5	1
	0	1	0	24	4	1
	1	1	0	25	5	1
	1	1	0	25	4	1
	1	1	0	20	4	0.25
	1	1	0	19	4	1
	1	1	0			
1		0	0	29	4	2
	0	0	1			
	0	0	1			
	0	0	1			
	1	0	1			
	0	1	0	27	5	1
1	0	0	1			
	0	1	0	30	5	1
	0	1	0	31	5	1
	1	1	0	26	4.5	1
	0	0	1			
	0	0	1			
	1	1	0	22	4	0
1	0	1	0	31	3	1
	0	0	1			

For peer review only

		0	0	27	4	1
	1	1	0	27	5	1
	0	1	0	27	4	0.5
	1	1	0	23	5	1
	1	1	0	27	4	1
	1	1	0	27	4	1
1		1	0	28	4	0
1		0	0	19	5	1
	0	1	0	27	4	1
	1	1	0	23	4	1
		0	0	26	4	1
	1	1	0	27	4	2
		0	0	28	5	0.25
	0	1	0	28	4.5	1
	1	0	1	19	4	1
	1	0	1	19	5	1
1	1	0	1	19	4	1.5
	0	1	0	26	4	0.75
		0	0	27	4	1
	0	1	0	28	5	1
		0	0	26	5	0
	1	1	0	27	4	1
	0	1	0	30	4	0.75
	0	1	0	27	4	1
	1	1	0	22	4	1
1		0	0	24	4	0.25
	0	0	1	15	3	0
	0	0	1			
		0	0	27	5	1
		0	0	26	4	2
1		0	0	22	4	1
1		0	0	25	5	1
	0	1	0	28	4	1
	0	1	0	27	4	1
	0	1	0	26	4	0.75
	1	1	1	26	4	1
	0	1	0	27	4	1
	0	1	0	31	4	1
1		0	0	28	5	1
	0	0	1			
	0	0	1			
1		0	0	28	4	1
1		0	0	26	3	1
	0	0	1	26	4	1.75
	0	1	0	28	3.5	1
	1	0	1			
	0	0	1			
	0	1	0	27	4	1.5
	0	1	0	27	5	1
	1	1	0	23	5	1

For peer review only

For peer review only - http://bmjopen.bmj.com/site/about/guidelines.xhtml

		0	0	28	4	1
1		0	0			
	1	1	0	23	4	1
	1	1	0	23	4	1
	0	0	1			
1	0	1	0	28	5	1
		0	0	26	4	1
	1	1	0	20	4	1
	1	1	0	20	4	1
	1	0	1			
	0	1	0	28	4	1
	0	1	0	26	4	2
	0	1	0	27	4	1
	0	1	0	26	4	1
	0	1	0	27	4	1
	0	1	0	27	5	1
	0	1	0	28	4	2
	1	1	0	21	4	1
	1	0	1			
1	0	1	0	27	5	1
	0	0	1			
	1	1	0	23	4	1.5
	1	1	0	21	4	1
	1	1	0	23	4	1
	1	1	0	22	4.5	1
	0	0	0	23	4	1
	1	1	0	25	5	1
	1	0	0	24	4.5	1
	0	0	0	24	4.5	1
	1	1	0	27	4	1
1	1	1	0	24	5	1
	0	0	1			
		0	0	25	4	2
	0	1	0	16	4.5	1
	0	0	1			
	0	1	0	25	5	0
	0	0	1			
	1	1	0	21	4	1
1		0	0	30	5	0
		0	0	27	4	0.5
	0	1	0	25	3	2
1	0	1	0	18	4	1
	0	0	1			
		0	0	27	4	1
	0	1	0	28	4	1
		0	0	27	4	1
1		1	0	27	4	1
		0	0	20	4	1
1		0	0	29	5	0
1		0	0	27	4	1

For peer review only

	0	1	0	27	5	0
1	0	1	0	32	5	1
	0	0	0	29	4	1
	0	1	0			
1		0	0	27	5	1
1	0	1	0	20	5	1
1		0	0	13	3	1
1		0	0	12	3	0
		0	0	27	4	0.5
	0	1	0	27	5	0
	0	1	0	27	4	0
	0	1	0	27	4	1
		0	0	25	4	1
	0	0	0	24	5	0
	0	1	0	23	5	1
1	1	1	0	23	5	1
	0	0	1			
	0	1	0	24	4	1
	0	1	0	28	5	1
	0	1	0	27	5	1
1	1	0	0	24	5	1
	0	0	1			
	0	0	1			
		0	1			
	0	0	1			
		0	1			
	0	0	1			
	0	0	1			
	1	0	1			
1	1	0	0	24	4	1
	1	0	0	25	5	1
	1	0	1			
	0	1	0	29	5	1
	0	0	1			
	1	0	0	24	4	1
	0	1	0	25	4	1
	0	1	0	25	4	2
	0	1	0	25	4	1
	0	1	0	25	4	0
1		0	0	18	3	1
1	1	0	0	24	5	1
	0	1	0	30	5	1
1		0	0	25	5	1
	0	0	1			
	0	1	0	24	4	1
	0	1	0	25	4	1
	1	0	0	23	4	1
	0	1	0	27	5	0
		1	0	30	5	1
1		1	0	22	5	0.75

For peer review only

	0	1	0	28	5	1
	0	1	0	27	4	1
1	1	0	0	24	5	1
1	0	1	0	26	5	1
1		1	0	26	4	1
	0	0	1			
	0	0	1			
	0	1	0	27	4	0.5
	1	1	0	23	4	1.5
	1	1	0	23	4	1
	0	1	0	26	4.5	1
	0	0	1			
	1	1	0	25	5	1
	0	0	1	19	5	0.5
	0	0	0			
	0	0	0			
	0	1	0	27	4	1
	1	1	0	26	5	1
	0	0	1			
	0	1	0	28	5	1
	0	1	0	27	4	0.5
	1	1	0	25	5	1
	0	1	0	27	4	1
	0	1	0	27	5	1
	0	1	0	31	5	1
	0	1	0	31	4	1
	0	1	0	30	4	1
	0	1	0	29	4	1
1	0	1	0	24	5	1
		0	0	28	4	1
		0	0	27	4	1
1		0	0	28	4	1
	0	1	0	25	5	1
	0	1	0	27	4	1
	0	1	0	27	5	1
	0	0	1			
	1	1	0	26	5	1
	1	1	0	25	4	1
	0	1	0	25	5	1
		0	0	25	4	1
	1	1	0	27	4	1
	0	0	1			
	0	0	1			
	1	1	0	23	5	1
		0	0	28	3	1
	0	0	0	25	4	2
		0	0	26	4	1
	1	1	0	26	5	0
	0	1	0	23	4	0
	0	1	0	23	4	0

For peer review only

	0	1	0	24	5	1
1	1	0	0	25	4	1
	0	0	0	25	5	0
	0	0	0	25	5	1
	1	1	0	24	4	1
	1	0	0	25	5	1
	1	0	0	26	4	1
	1	0	0	25	4	1
	0	0	0	24	5	1
	1	1	0	25	4	1
	0	0	0	24	5	1
	0	1	0	26	5	1
	1	1	0	22	4	1
1	0	0	0	25	5	1
	1	1	0	24	4	0
	1	1	0	26	5	0
	0	0	0	25	5	0
	1	1	0	25	4	2
	0	1	0	25	4	2
	0	1	0	25	4	1
1	0	0	0	25	5	1
1	0	1	0	27	4	1
	0	0	0	26	5	0.75
	0	1	0	25	4	2
	0	1	0	26	4.5	1
	0	1	0	26	4	1
	0	0	0	25	5	1
	0	1	0	25	5	1
	0	0	0	26	5	0
1	0	1	0	25	5	1
	0	0	0	25	5	0
1	0	0	0	25	5	1
	1	1	0	26	5	0
1	0	0	0	25	5	1
1	0	0	0	25	5	0
	0	0	0	26	4	1
	1	1	0	27	5	1
	1	1	0	27	5	1
	0	1	0	27	4	1
	0	1	0	27	4	0.5
	1	1	0	27	5	1
	1	1	0	27	5	1
	1	1	0	27	5	1
	1	0	1			
	1	1	0	27	4	1
1		0	0	28	4	1
		0	0	27	5	0.5
	0	0	1			
1		0	0	28	4	1.25
	0	1	0	27	3	0

For peer review only

	0	1	0	27	4	1
	1	1	0	26	4.5	1
	0	0	1			
	1	1	0	24	4	1
	0	0	1			
		0	0	27	4	0
	0	0	1			
		0	0	24	4.5	1
	1	1	0	27	5	1
1		0	0	20	4	0.25
	0	1	0	31	4	1
		0	0	26	4	1
		0	0	26	4	1
		0	0	22	4	1
		0	0	27	5	1
	0	0	1			
	1	1	0	27	4	1
	1	1	0	27	5	1
1		0	0	30	5	1
	1	1	0	25	5	1
	0	1	0	28	4	1
1		0	0	30	5	0
	0	0	1			
	0	1	0	26	4	1
	1	0	1			
		0	0	26	5	1
	0	1	0	27	5	0
	1	0	1			
1		0	0	26	4	0
		0	0	27	4	1
	0	1	0	27	4	1
	0	0	1			
	1	1	0	27	5	1
	0	1	0	27	4	2
		0	0	27	4	1
	1	1	0	25	4	2
	0	0	1			
1		0	0	28	5	1
	1	0	0	27	5	1
1		0	0	30	3	0.75
	1	1	0	27	5	1
	0	1	0	27	5	1
	0	0	1			
	1	1	0	27	5	1
		0	0	28	5	1
	0	0	1			
	0	1	0	25	4	1
	1	1	0	27	4	1
	1	1	0	25	4	0
	0	0	1			

For peer review only

	0	1	0	27	5	1
	1	1	0	27	5	0.5
	0	1	0	26	5	1
	0	1	0	27	5	0
	0	0	1			
1		0	1			
	1	0	1			
	0	1	0	23	3	2
	1	1	0	27	5	1
	1	1	0	22	4	0
	1	1	0	27	4	1
	1	0	1			
	1	0	1			
	1	1	0	23	4	1
	0	1	0	26	4	1
1	1	0	0	25	5	1
1	0	1	0	26	5	1
		0	0	27	4	0.5
1	0	0	0	23	4	1
	0	0	1			
	0	0	1			
	0	0	1			
	0	0	1			
	0	0	1			
	0	0	1			
	0	0	1	26	4	2
1	0	0	0	29	5	1
	0	1	0	25	4	1
1	1	0	0	25	5	1
	1	0	1			
	0	1	0	26	5	1
	0	1	0	25	5	1
	1	1	0	26	4	1
1	1	0	0	26	5	1
	0	0	1			
	0	0	1			
	0	0	0	24	4	0
	0	0	0	24	5	1
	1	1	0	26	4	1
	1	1	0	27	5	1
		0	0	17	3	1
1		0	0	13	3	0
1	0	1	0	26	4.5	1
		0	0	11	4	1.5
		0	0	19	3	0
	0	1	0	26	4	2
	0	1	0	27	4	1
	1	1	0			
1	0	1	0	25	5	1
1	0	0	0	25	5	1

For peer review only

	1	1	0			
	1	0	1			
	1	0	1			
	0	1	0	27	5	0
1	0	0	0	26	4	1
	0	1	0	26	5	1
	0	1	0	26	5	1
1	1	0	0	25	4	1
1		0	0	26	5	0
	0	0	1			
1		0	0	18	4.5	1
	0	1	0	27	4	1
	0	1	0	25	5	1
		0	0	25	5	1
1		0	0	25	4	1
1	1	0	0	24	4	1
	0	1	0	16	4	0
1		1	0	28	4	1
1	0	0	0	24	5	1
1	0	0	0	25	4	1
1	1	0	0	26	5	1

For peer review only

For peer review only - http://bmjopen.bmj.com/site/about/guidelines.xhtml

Min(round	Max(rounc	N(round3)	Median(ro	IQR(round	Min(round	Max(round3)
		19	5	0	4	5
		15	3	1	1	4
		19	4	0	2	5
3	5					
4	5					
3	5					
3	5					
4	5					
		19	5	1	3	5
3	5					
4	5					
3	5					
2	5	19	4	1	1	5
2	5					
3	5					
2	5					
2	4					
1	5					
4	5					
3	5					
3	5					
3	5					
3	5					
		19	4	1.5	3	5
3	5					
2	5	18	3.5	1	2	5
		19	4	1	4	5
3	5					
2	5					
2	5					
		19	4	1.5	2	5
2	5					
3	5					
1	5	15	4	0.5	3	5
2	5					
4	5					
2	4					
3	5					
3	5					
		5	4	1	3	5
		18	4	0	4	5
4	5					
4	5					
		17	4	0	4	5
2	5	8	4	0.25	3	4
3	5					
3	5					
1	5	19	4	1	1	5

For peer review only

3	5					
2	5					
		15	3	1.5	1	4
		18	4	1.75	1	5
		17	4	2	1	5
		18	5	1	1	5
		17	4	2	2	5
		19	4	1	3	5
		19	5	1	4	5
2	5					
4	5					
		18	5	1	4	5
		18	5	0	4	5
		17	4	1	4	5
		18	5	1	3	5
3	5					
4	5					
4	5					
3	5					
2	5					
		13	3	2	1	4
		13	4	1	2	5
		13	5	1	4	5
2	5	6	3.5	1	3	5
1	5	7	5	1	4	5
2	5					
1	5					
2	4	17	3	0	1	4
3	5					
3	5					
3	5					
1	5	17	3	1	3	5
		19	5	0.5	3	5
4	5					
3	5					
		18	4.5	1	4	5
		18	5	1	4	5
		17	4	1	4	5
1	5					
4	5					
3	5	4	3	0.25	3	4
2	5					
4	5					
4	5					
1	5	19	3	0	1	4
4	5					
2	5					
3	5					
2	5	18	4	1	3	5
2	5					

For peer review only

3	5					
3	5					
2	5					
3	5					
1	5	18	3	1	2	5
2	5					
3	5					
		17	3	1	3	5
2	5					
		18	3	1	2	5
3	5					
		12	4	1	3	5
4	5					
4	5					
4	5					
2	5					
1	5					
4	5					
2	5	18	4	1	3	5
3	5					
3	5					
3	5					
1	5	18	4	1	3	5
		18	4	1	4	5
3	5					
2	5	17	4	1	3	5
3	5					
4	5					
4	5					
3	5					
3	5					
3	5					
3	5					
3	5					
		15	4	0	3	5
2	5	19	4	1	3	5
		18	4.5	1	4	5
		7	5	1	3	5
		4	3.5	1.25	3	5
		16	4	1	4	5
4	5					
		17	5	0	2	5
2	5					
4	5					
4	5					
		18	5	1	4	5
		19	5	1	4	5
3	5					
1	5	19	4	1	3	5
		19	4	1	3	5

For peer review only

2	5					
3	5					
2	5					
3	5					
3	5					
3	5					
3	5					
3	5					
2	5					
3	5					
2	5					
2	5					
4	5					
3	5					
3	5					
4	5					
2	5					
3	5					
2	5					
3	5					
3	5					
1	5					
1	5					
1	5					
3	5					
2	5					
2	5					
		18	4	1	3	5
1	5					
1	5	17	4	0	1	5
2	5					
4	5					
3	5					
3	5					
3	5					
3	5					
1	5					
1	5					
2	5					
		13	4	1	2	5
		12	3	0.5	3	5
2	5					
2	5	18	4	0.75	3	5
2	5	17	4	1	3	5
1	5					
		19	4	1	3	5
		19	5	0	3	5
1	5	3	4	0.5	4	5
3	5					
3	5					

For peer review only

2	5					
		17	4	1	2	5
2	5					
3	5					
		19	5	1	4	5
4	5					
3	5					
4	5					
4	5					
		16	5	1	3	5
3	5					
1	5					
3	5					
2	5					
2	5					
3	5					
1	5	19	4	1	1	5
3	5					
		15	4	0.5	2	5
3	5					
		18	5	1	3	5
3	5	18	4	0.75	3	5
3	5					
3	5					
3	5					
4	5					
3	5					
2	5					
4	5					
1	5					
3	5					
		19	5	0	4	5
1	5					
2	5					
		16	4	1	2	5
4	5					
		19	5	0	4	5
3	5					
3	5					
2	5					
1	5	17	3	1	3	5
4	5					
		18	4.5	1	2	5
1	5					
2	5	0				
4	5					
3	5					
2	5					
4	5					
2	5					

For peer review only

4	5					
3	5					
2	5					
		18	4	0	3	5
4	5					
4	5					
3	5	14	3	1	3	4
2	5	14	3	1	3	4
2	5					
4	5	19	5	1	3	5
3	5	19	4	0	3	5
2	5	19	3	0.5	2	4
2	5					
4	5					
4	5					
3	5					
		14	4	0.75	2	5
3	5					
3	5					
4	5					
3	5					
		13	3	1	1	5
		15	3	1	1	5
		15	3	2	1	5
		15	4	1	2	5
		14	4	2	3	5
		14	5	1	3	5
		13	3	1	2	5
		18	4	0	3	5
2	5					
3	5					
		14	4	0	2	5
4	5					
		13	4	1	2	4
2	5					
2	5					
2	5	19	4	1	2	5
3	5					
3	5					
2	4	15	3	1	3	5
2	5					
3	5					
3	5					
		18	4	1	4	5
1	5					
3	5					
2	5					
4	5					
4	5					
3	5					

For peer review only

3	5					
2	5					
4	5					
4	5					
4	5					
		19	5	0	4	5
		19	5	1	4	5
3	5					
2	5	18	4	0	3	5
1	5					
3	5					
		18	4.5	1	4	5
4	5					
4	5					
		17	3	1	1	5
		17	3	1	1	5
3	5					
3	5					
		18	5	1	4	5
4	5					
2	5					
3	5					
2	5					
3	5					
4	5					
2	5					
2	5					
2	5					
3	5					
2	5					
2	5					
3	5					
3	5					
3	5					
4	5					
		19	5	1	3	5
3	5					
3	5					
3	5					
3	5					
1	5					
		17	3	1	2	5
		15	4	1	2	5
4	5					
2	5					
2	5	19	4	0	3	5
2	5					
4	5					
3	5					
3	5					

For peer review only

For peer review only - http://bmjopen.bmj.com/site/about/guidelines.xhtml

4	5					
1	5					
4	5					
3	5					
1	5					
1	5					
3	5					
2	5					
4	5					
3	5					
4	5					
4	5					
1	5					
3	5					
3	5					
4	5					
4	5					
2	5					
2	5	18	4	1	3	5
2	5					
3	5					
1	5					
4	5					
2	5	18	4	1	3	5
3	5					
2	5					
4	5					
4	5					
4	5					
2	5					
4	5					
2	5					
4	5					
2	5					
4	5					
1	5					
4	5					
3	5					
3	5					
3	5					
3	5					
4	5					
3	5					
		17	4	0	3	5
4	5					
2	5					
4	5					
		19	5	1	3	5
2	5	19	4	1	3	5
1	5	19	3	1	2	5

For peer review only

2	5					
4	5					
		3	4	1	3	5
2	5					
		16	4	0	3	5
2	5					
		18	4	1	3	5
3	5					
4	5					
3	4					
1	5					
2	5					
3	5					
3	5					
3	5					
		19	4	1	3	5
1	5					
4	5					
3	5					
4	5					
2	5					
4	5					
		18	5	0	4	5
2	5					
		19	4	2	2	5
3	5					
5	5					
		18	4	1	3	5
3	5					
2	5					
4	5					
		17	4	1	2	5
4	5					
2	5	12	4	0	3	5
2	5					
3	5	16	4	0.25	3	5
		17	4	1	3	5
3	5					
4	5					
1	5	18	3	1	1	5
4	5					
3	5					
		16	3.5	1	1	5
3	5					
3	5					
		18	4.5	1	4	5
1	5					
2	5					
1	5					
		17	5	1	2	5

For peer review only

4	5					
4	5					
1	5					
4	5					
		19	4	0.5	4	5
		19	4	0	2	5
		17	5	1	3	5
2	5	14	2	1	2	4
3	5					
3	5					
4	5					
		18	4	0.75	3	5
		18	5	1	3	5
2	5					
3	5					
3	5					
3	5					
2	5					
3	5					
		16	4	1	3	5
		17	4	1	3	5
		17	4	1	3	5
		15	4	1.5	2	5
		18	4	0.75	2	5
		15	4	1	3	5
3	5	17	4	0	3	5
3	5					
1	5					
4	5					
		17	5	1	2	5
4	5					
2	5					
3	5					
4	5					
		8	4	1.25	3	5
		19	4	0.5	2	5
2	5					
4	5					
3	5					
4	5					
1	5	15	3	1	3	4
1	4	11	3	0	2	3
3	5					
3	5	12	3	1	3	4
2	4	16	3	0	3	5
2	5	19	4	1	3	5
2	5					
		19	5	1	3	5
3	5					
3	5					

For peer review only

		31	4	1	3	5
		19	4	1	3	5
		19	4	1	3	5
4	5					
2	5					
4	5					
4	5					
3	5					
2	5					
		18	4	1	3	5
3	5					
2	5					
3	5					
3	5					
3	5					
3	5					
3	5					
2	5					
3	5					
2	5					
4	5					

For peer review only

Can process mapping and a multi-site Delphi of perioperative professionals inform our understanding of system-wide factors that may impact operative risk?

Stubbs DJ, Bashford T, GIlder FJ, Nourallah B, Ercole A, Levy N, Clarkson PJ

Supplementary Material:

This document contains supplementary figures, **further material is available as two separately available files.**

1. A **.xlsx format** spreadsheet showing all suggestion voted on across the three rounds of the Delphi
2. A **html format** document with an interactive collapsible view of the hierarchy of questions used in the second and third delphi rounds. Details on how to browse this can be found in section 6 of this document

Contents of this document

1. **Annotated process maps demonstrating the care of elective (a) and emergency (b) vascular surgical patients**
2. **List of risk scores used to provide a 'minimum' dataset for voting on in Delphi round two**
3. **Component variables from these risk scores**
4. **Hierarchy of question groupings used in Delphi rounds two and three**
5. **'Novel' variables from the Delphi**
6. **Using the interactive html file**

For peer review only

Supplementary Figure 1: Process map of elective vascular care used to identify perioperative stakeholders.

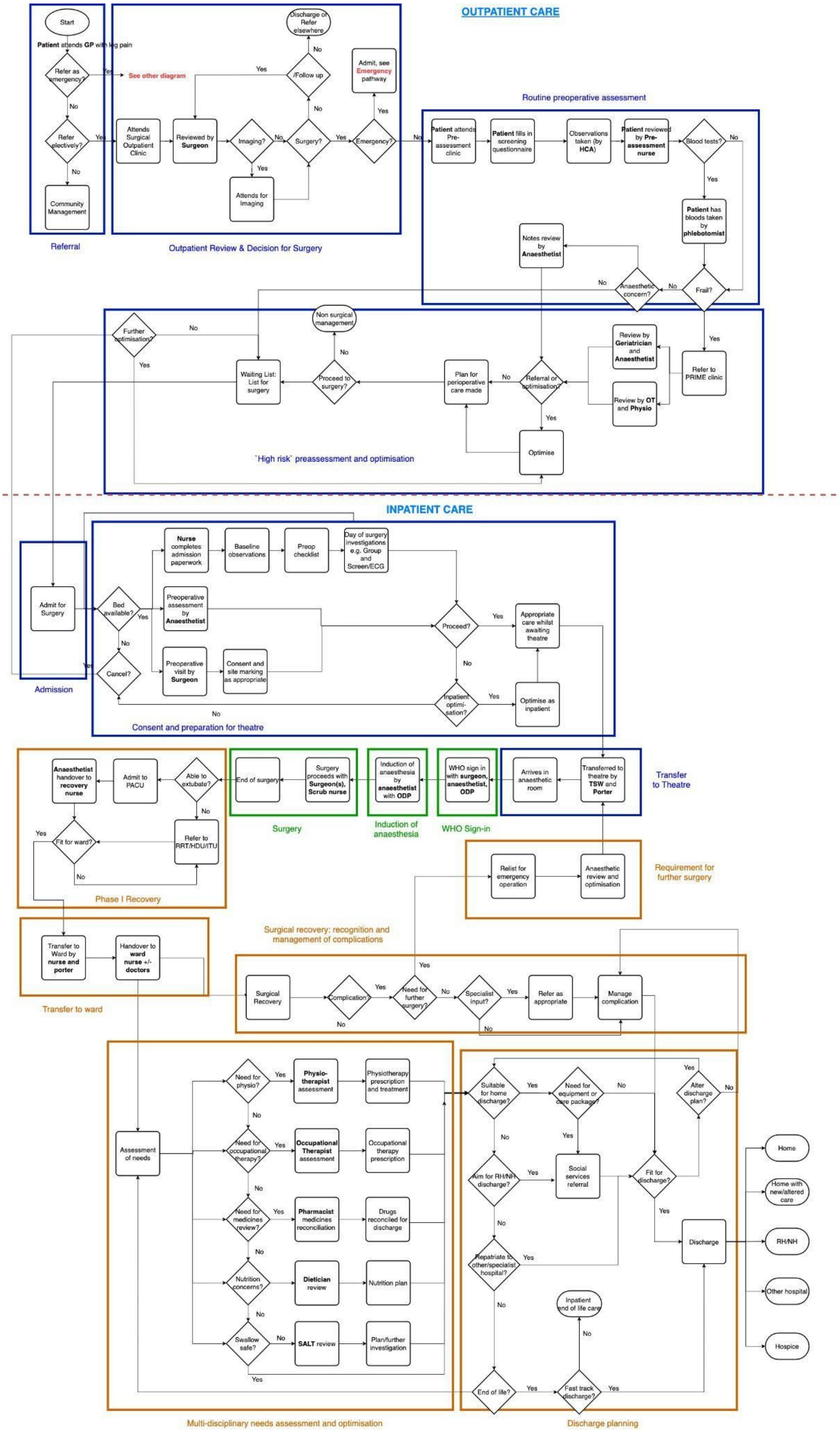

**Supplementary Figure 2: Process map of emergency vascular care used to identify perioperative stakeholders. **

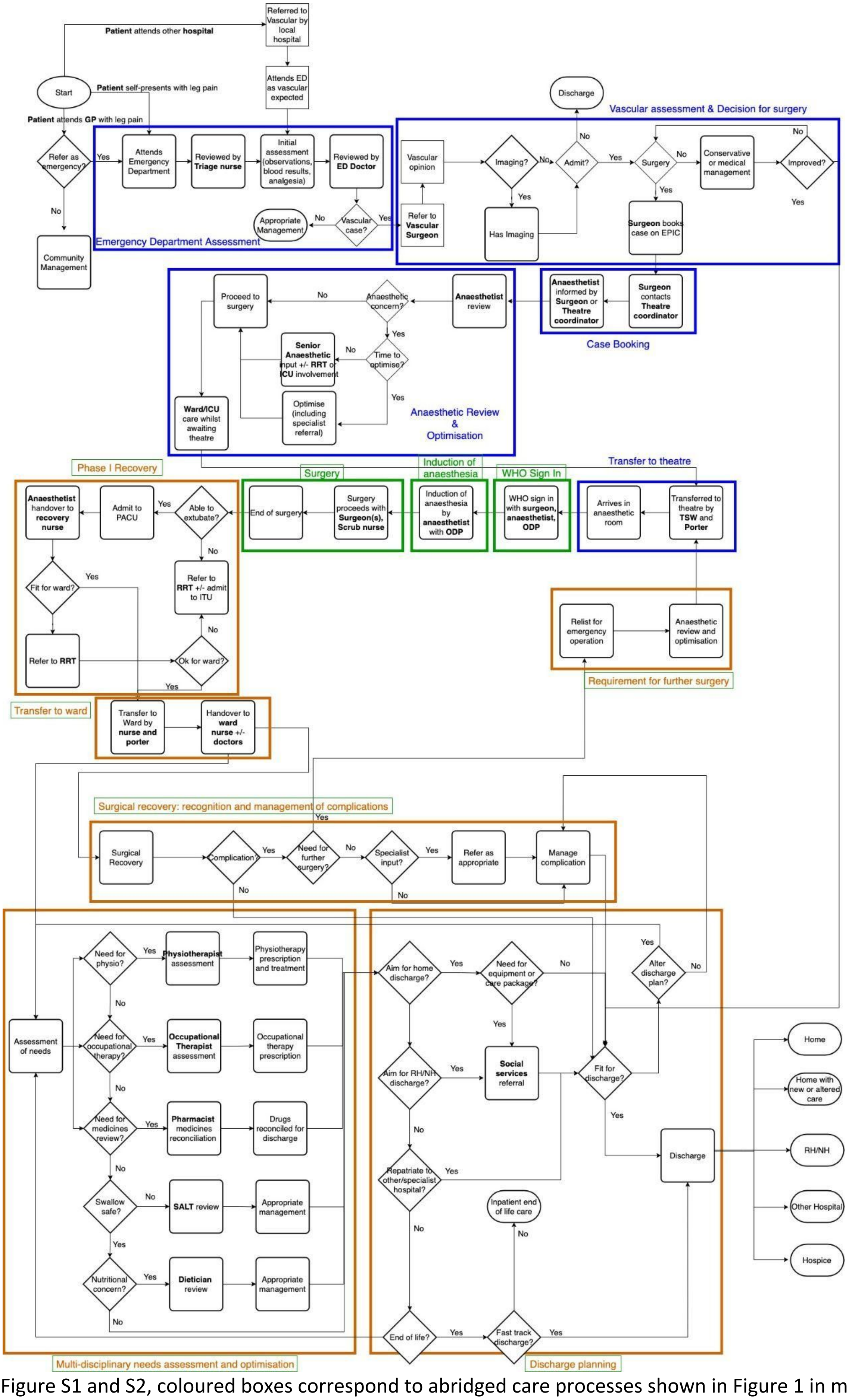

(In both Figure S1 and S2, coloured boxes correspond to abridged care processes shown in Figure 1 in main text)

Risk scores used in formation of a 'minimum' dataset for voting in rounds two and three

ASA score Nutritional risk index
BHOM Otago Surgical outcome score
Charlson Comorbidity Index Perioperative Mortality Risk Score
APACHE POSSUM
Delaware P-POSSUM
Detsky index RCRI
E-PASS SAPS II
Edmonton Frail scale Subjective Global assessment
ICISS Surgical Apgar
IRIS Surgical mortality score
Maastricht Nutritional Assessment Surgical Risk Scale
Mini nutritional assessment Surgical Risk Score (Donati)
MPM III

Supplementary Table 1: List of risk scores identified as having been validated in heterogeneous surgical cohorts in a 2013 systematic review[1]. These scores were deconstructed (see **Supplementary Figure 3**) to provide the minimum components of any dataset resulting from the Delphi. *ASA = American Society of Anesthesiologists, BHOM – Biochemistry & Haematology Outcome Model, APACHE = Acute physiology & chronic health evaluation, DELAWARE = Dense Laboratory Whole blood applied Risk Estimation, E-PASS = Estimation of physiological ability and surgical stress, ICISS = ICD injury severity score, IRIS = Identification of risk in surgical patients score, MPM III = Mortality prediction model (III), POSSUM = Physiological and Operative Severity Score for the enUmeration of Mortality and morbidity, P-POSSUM = Portsmouth modification of POSSUM, RCRI = Revised cardiac risk index, SAPS II = Simplified acute physiology score II*

For peer review only

1. Moonesinghe SR, Mythen MG, Das P, Rowan KM, Grocott MP. Risk stratification tools for predicting morbidity and mortality in adult patients undergoing major surgery: qualitative systematic review. *Anesthesiology* 2013; **119**: 959–81.

Preoperative

Acute Comorbidity
- Acute renal failure
- CVA
- GI Bleed
- Intracranial Mass effect
- SIRS
- Pneumonia
- Ventilation
- Impaired sensorium

Preop Physiology
- Temperature
- Urine Output
- Raised JVP
- Systolic Blood Pressure
- Pulse Rate
- GCS

Laboratory
- Albumin
- Haematocrit
- Creatinine >2mg/dL
- Bicarbonate
- SGOT (Serum Glutamic Oxaloacetic Transaminase)
- Serum Transferrin
- PaO2
- pH
- Bilirubin
- Glucose
- ALT
- CRP
- Cholesterol
- CK MB
- Platelets
- Triglycerides
- Nucleated RBCs
- haemoglobin
- White Cell Count
- Urea
- Sodium
- Potassium
- PT, APTT, INR
- Transthyretin
- Lymphocyte Count

Drugs
- Chronic Steroids
- Diuretics
- Digoxin
- Medication use > 3
- Medication Use > 5

Admission related
- Acute admission
- Acute surgery
- CPR pre-admission
- Pre-op stay > 3 days or ≦ 3 days

Patient
- Weight Loss > 10lB
- Decreased Grip Strength
- Exhaustion (effort/motivation)
- Low Physical Activity
- Slow Walking Speed
- Age
- Gender
- ASA Score
- Smoker
- BMI
- Frailty
- Cognition
- General Health (N of hospital admissions, subjective)
- Mood (sad/depressed)
- Continence
- Functional performance: timed up and go, bedbound/housebound
- EtOH
- DNR status
- Functional status
- Functional independence
- Social Support
- Performance status
- Self view of health

Nutrition
- Able to eat independently
- Decreased Appetite
- High metabolic requirement
- Triceps skinfold thickness
- Meals/day
- Protein intake/day
- Fruit/vegetable intake
- GI symptoms affecting intake (e.g. Dysphagia, diarrhoea)
- Mid-arm circumference
- Mid-calf circumference
- Loss of body fat
- Loss of muscle
- Normal fluid intake (cups/day)
- Oedema

Comorbidities
- 'Significant' Aortic Stenosis
- Acute psychological or physical illness in last 3/12
- Acute Renal Failure
- AIDS/HIV
- Angina
- Ascites
- Bleeding Disorders (includes Anticoagulants)
- Brain tumour
- Cardiomegaly
- Cardiomyopathy
- CCF
- Cerebrovascular Disease
- Chemotherapy in </= 30 D
- Chronic Pulmonary Disease
- Chronic Renal failure
- Cirrhosis
- COPD (on treatment, hospitalised, impaired ADLs)
- Dementia
- Diabetes
- Dialysis
- Dyspnoea (nil, mod exertion, rest)
- ECG (eagle = q wave)
- Hemiplegia/Paraplegia
- Hypertension
- Liver Disease
- Malignancy
- Metastases
- MI (Goldman = last six months)
- Oesophageal varices
- Open wound
- Paraplegia/Quadriplegia/Hemiplegia
- Peripheral Vascular Disease
- Pregnancy
- Previous Cardiac Surgery
- Previous PCI
- Pulmonary oedema
- Radiotherapy in </= 90 D
- Renal Disease
- Rest pain/gangrene
- Rheumatological Disease
- Sepsis
- Severe Heart Disease (NYHA III or IV or severe arrhythmia – not defined)
- Severe Pulmonary Disease (VC < 60 FEV1 <50)

Intraoperative

Surgical/Operative
- Blood Loss
- Blood Loss g/body weight
- Intraperitoneal, Intrathoracic, Aortic
- Length of surgery
- Malignancy
- Number of procedures
- Peritoneal Soiling
- Seniority of operator (Consultant, Registrar)
- Skin Incision (0=Laparoscopic 1=Laparotomy or Thoracotomy 2=Both)
- Timing of surgery
- Wound classification (contaminated/not contaminated)

Anaesthetic
- Principal anaesthetic technique
- Airway Trauma
- Lowest MAP
- Lowest HR

Complication
- Intraoperative MI/CPR

Postoperative

Complication
- Acute renal failure
- Blood transfusion
- Cardiac Arrest
- Coma
- CVA
- DVT
- Flap failure
- Lowest HR
- Lowest MAP
- Need for ventilation >48 hours
- Peripheral Nerve Injury
- Post op MI
- Postop Pneumonia
- Pulmonary Embolism
- Sepsis
- Surgical site infection (superficial, deep)
- Unplanned ICU admission
- Unplanned intubation
- Unplanned Reoperation
- Urinary tract infection

Supplementary Figure 3: Components of validated perioperative scores introduced into Delphi at round two. These variables were identified by deconstructing the risk scores shown in Supplementary Table 1. Groupings are artefactual for clarity and to delineate key temporal aspects of the use of the identified risk scores. Each of these variables were included in Delphi round two, regardless of whether they were present in free-text suggestions from the panel in round one.

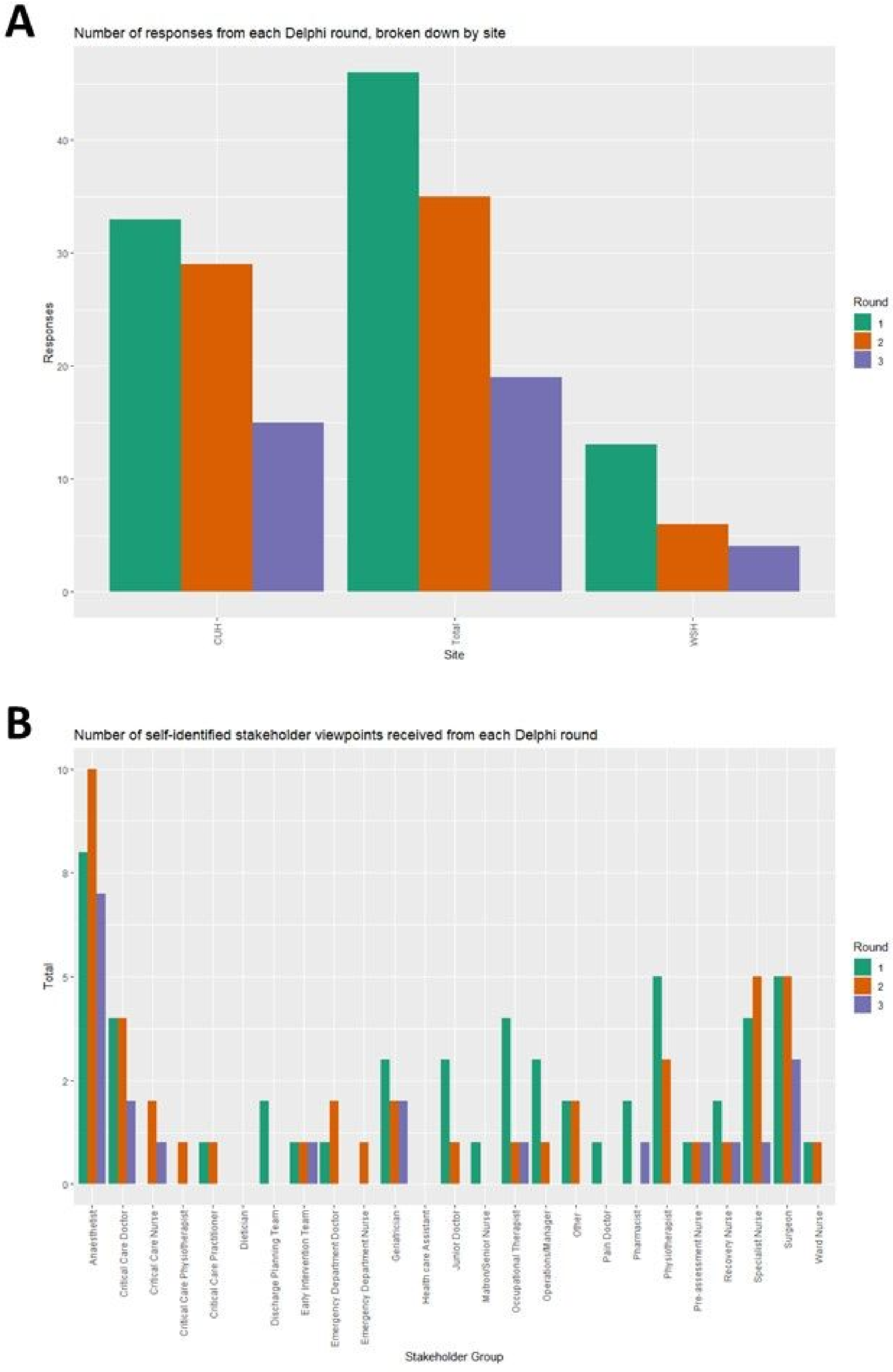

Figure 3: Response rate and respondent profile by round for a Delphi exercise to gain a consensus view on factors impacting on outcome in older surgical patients. *CUH = Cambridge University Hospitals (a tertiary care centre), WSH = West Suffolk Hospital (a secondary care centre)*

Variables suggested by the Delphi panel that did not feature in the development of perioperative risk scores or in a systematic review of structural and process quality indicators.

VARIABLE	DOMAIN	GROUP
**Opioid deprescribing pre-discharge**	Admission	Analgesic Plan
**Opioid dose**	Admission	Analgesic Plan
**Chest injuries**	Admission	Circumstances
**Delayed presentation**	Admission	Circumstances
**Disruption to a patient's routine**	Admission	Circumstances
**Bed Occupancy Rates**	Organisation	Hospital Performance
**Subdural haematoma**	Admission	Circumstances
**Trauma**	Admission	Circumstances
**Indication for surgery (pathology)**	Admission	Details of Surgery
**Orthopaedic v Non-Orthopaedic**	Admission	Details of Surgery
**Delayed Transfer of Care (DToC) Rates**	Organisation	Hospital Performance
**Delayed Transfer of Care**	Admission	Discharge Planning
**ED Admission wait times**	Organisation	Hospital Performance
**Empathy**	Organisation	Staffing
**Enthusiasm and engagement**	Organisation	Staffing
**Health Service Organisation**	Organisation	Health Services Organisation
**Hospital Performance & Strain**	Organisation	Hospital Performance
**Intensive care occupancy**	Organisation	Hospital Performance
**Details of Surgery**	Admission	Intraoperative
**Intraoperative CO2 Control**	Admission	Intraoperative
**Intraoperative Complications**	Admission	Intraoperative
**Medical cover**	Organisation	Staffing
**Intraoperative Physiological Control**	Admission	Intraoperative
**ODP Seniority**	Admission	Intraoperative

**Operator Fatigue**	Admission	Intraoperative
**Scrub nurse Seniority**	Admission	Intraoperative
**Number of outlying patients**	Organisation	Hospital Performance
**Number of patients cared for**	Organisation	Health Services Organisation
**Number of vacant posts**	Organisation	Hospital Performance
**Nursing cover**	Organisation	Staffing
**Occupational therapy cover**	Organisation	Staffing
**Escalation plan made**	Admission	Postoperative
**Organisational culture**	Organisation	Staffing
**Overnight stay in ED**	Organisation	Hospital Performance
**Number of attachments**	Admission	Postoperative
**Pharmacy cover**	Organisation	Staffing
**Physiotherapy cover**	Organisation	Staffing
**Referrals & Reviews**	Admission	Postoperative
**Time to occupational therapist review**	Admission	Postoperative
**Time to pharmacist review (e.g. for medicines reconcilliation)**	Admission	Postoperative
**Anastomotic Breakdown**	Admission	Postoperative Complications
**Practice type**	Organisation	Health Services Organisation
**New diagnosis of chronic conditions (e.g. Dementia)**	Admission	Postoperative Complications
**Poor Wound Healing**	Admission	Postoperative Complications
**Postoperative Dysrhythmia**	Admission	Postoperative Complications
**Postoperative Pain**	Admission	Postoperative Complications
**Postoperative Pneumonia**	Admission	Postoperative Complications
**Postoperative sepsis**	Admission	Postoperative Complications
**DNR status & Escalation plan**	Admission	Preoperative
**How long patients expect to stay in hospital**	Admission	Preoperative
**Primary Care Structure**	Organisation	Health Services Organisation

**Procurement**	Organisation	Policies and Procedures
**Prehabilitation program**	Admission	Preoperative
**Respiratory Virus Rates**	Organisation	Hospital Performance
**Season**	Organisation	Hospital Performance
**Therapy plans**	Admission	Preoperative
**Staffing**	Organisation	Staffing
**Staffing levels**	Organisation	Staffing
**Able to follow commands preoperatively**	Admission	Preoperative Physiology
**Tertiary admission/transfer**	Organisation	Health Services Organisation
**Preoperative CVS impairment**	Admission	Preoperative Physiology
**Preoperative Delirium**	Admission	Preoperative Physiology
**Preoperative Physiology**	Admission	Preoperative Physiology
**Preoperative Renal impairment**	Admission	Preoperative Physiology
**Preoperative Respiratory Failure**	Admission	Preoperative Physiology
**Ward moves/handovers**	Admission	Postoperative

Supplementary Table 2: List of variables that emerged during the Delphi that did not feature in the development of perioperative risk scores or in a systematic review of quality and structural process indicators. For references see text. Blue highlighting demonstrates factors related to the organisation of the healthcare organisation, Green highlights factors pertaining to the individual admission in question.

Using the Interactive Html File

An interactive html file **(DelphiQViewer.html)** is available as a separate supplementary file. By clicking on the interactive diagram you will be able to view the hierarchy used in presenting all options to the panelists for voting. The lowest level (or 'leaf') is the most granular level of suggestion that the panelists voted on.

Click on each node to expand lower levels, white nodes demonstrate the lowest level of each branch. As new branches expand **click and drag** on the surrounding area to re-center the diagram as you go. Zooming in and out can be achieved using the **scroll wheel** of your mouse.

For peer review only - http://bmjopen.bmj.com/site/about/guidelines.xhtml

BMJ Open

Can process mapping and a multi-site Delphi of perioperative professionals inform our understanding of system-wide factors that may impact operative risk?

Journal:	*BMJ Open*
Manuscript ID	bmjopen-2022-064105.R1
Article Type:	Original research
Date Submitted by the Author:	26-Aug-2022
Complete List of Authors:	Stubbs, Daniel; University of Cambridge, Department of Engineering; University of Cambridge School of Clinical Medicine, Division of Anaesthesia<br>Bashford, Tom; University of Cambridge School of Technology, Engineering; Cambridge University Hospitals NHS Foundation Trust, Department of Anaesthesia<br>Gilder, Fay; Princess Alexandra Hospital NHS Trust<br>Nourallah, Basil; West Suffolk Hospital, Department of Anaesthesia<br>Ercole, Ari; Cambridge University Hospitals NHS Foundation Trust<br>Levy, Nicholas; West Suffolk Hospital, Department of Anaesthesia<br>Clarkson, John; University of Cambridge, Engineering Design Centre
**Primary Subject Heading**:	Anaesthesia
Secondary Subject Heading:	Surgery, Health informatics
Keywords:	ANAESTHETICS, SURGERY, Risk management < HEALTH SERVICES ADMINISTRATION & MANAGEMENT, QUALITATIVE RESEARCH, STATISTICS & RESEARCH METHODS

SCHOLARONE™
Manuscripts

I, the Submitting Author has the right to grant and does grant on behalf of all authors of the Work (as defined in the below author licence), an exclusive licence and/or a non-exclusive licence for contributions from authors who are: i) UK Crown employees; ii) where BMJ has agreed a CC-BY licence shall apply, and/or iii) in accordance with the terms applicable for US Federal Government officers or employees acting as part of their official duties; on a worldwide, perpetual, irrevocable, royalty-free basis to BMJ Publishing Group Ltd ("BMJ") its licensees and where the relevant Journal is co-owned by BMJ to the co-owners of the Journal, to publish the Work in this journal and any other BMJ products and to exploit all rights, as set out in our licence.

The Submitting Author accepts and understands that any supply made under these terms is made by BMJ to the Submitting Author unless you are acting as an employee on behalf of your employer or a postgraduate student of an affiliated institution which is paying any applicable article publishing charge ("APC") for Open Access articles. Where the Submitting Author wishes to make the Work available on an Open Access basis (and intends to pay the relevant APC), the terms of reuse of such Open Access shall be governed by a Creative Commons licence – details of these licences and which Creative Commons licence will apply to this Work are set out in our licence referred to above.

Other than as permitted in any relevant BMJ Author's Self Archiving Policies, I confirm this Work has not been accepted for publication elsewhere, is not being considered for publication elsewhere and does not duplicate material already published. I confirm all authors consent to publication of this Work and authorise the granting of this licence.

For peer review only

Can process mapping and a multi-site Delphi of perioperative professionals inform our understanding of system-wide factors that may impact operative risk?

Stubbs DJ[1a,b], Bashford T[2ab], Gilder FJ[3] Nourallah B[4], Ercole A[5], Levy N[6], Clarkson PJ[7]

1a: Wellcome Trust Research Fellow, Healthcare Design Group, Department of Engineering, University of Cambridge, Trumpington Street, Cambridge, CB2 1PZ. b: Honorary Specialist Registrar in Anaesthesia, of Anaesthesia, Department of Medicine, University of Cambridge, Addenbrooke's Hospital, Hills Road, Cambridge, CB2 0QQ,

2a: Assistant Professor of Healthcare Systems, University of Cambridge Department of Engineering; 2b: Consultant in Neuroanaesthesia, Cambridge University Hospitals NHS Foundation Trust, Addenbrooke's Hospital, Hills Road, Cambridge, CB2 0QQ

3: Medical Director, Princess Alexandra Hospitals Harlow, NHS Trust, Hamstel Road, Harlow, Essex, CM20 1QX

4: Core Trainee, Department of Anaesthesia, West Suffolk Hospital, Bury St Edmunds, Hardwick Lane, IP33 2QZ

5: Consultant Anaesthetist & Intensivist, University Division of Anaesthesia, Department of Medicine, University of Cambridge, Addenbrooke's Hospital, Hills Road, Cambridge, CB2 0QQ

6: Consultant Anaesthetist, Department of Anaesthesia, West Suffolk Hospital, Bury St Edmunds, Hardwick Lane, IP33 2QZ

7: Professor of Engineering Design, Healthcare Design Group, University of Cambridge Department of Engineering, Trumpington Street, Cambridge, CB2 1PZ

Correspondence to:

Dr DJ Stubbs
djs225@cam.ac.uk

Short Title: A Delphi study to understand perspectives on perioperative risk

KEYWORDS

- ANAESTHESIA
- SURGERY
- RISK MANAGEMENT
- QUALITATIVE RESEARCH
- STATISTICS & RESEARCH METHODS

Twitter Handles

@djramsaystubbs, @ari_ercole, @nicholaslevy, @fayjojo

For peer review only

Word count (excluding abstract, references, tables and figure legends): 3823

ABSTRACT

Objectives: To examine whether the use of process mapping and a multidisciplinary Delphi can identify potential contributors to perioperative risk. We hypothesised that this approach may identify factors not represented in common perioperative risk tools and give insights of use to future research in this area.

Design: Multidisciplinary, modified Delphi study

Setting: Two centres (one tertiary, one secondary) in the United Kingdom during 2020 amidst coronavirus pressures.

Participants: 91 stakeholders from 23 professional groups involved in the perioperative care of older patients. Key stakeholder groups were identified via process mapping of local perioperative care pathways.

Results: Response rate ranged from 51% in round one to 19% in round three. After round one, free text suggestions from the panel were combined with variables identified from perioperative risk scores. This yielded a total of 410 variables that were voted on in subsequent rounds. Including new suggestions from round two, 468/519 (90%) of the statements presented to the panel reached a consensus decision by the end of round three. Identified risk factors included patient level factors (such as ethnicity and socio-economic status); and organisational or process factors related to the individual hospital (such as policies, staffing, and organisational culture). 66/160 (41%) of the new suggestions did not feature in systematic reviews of perioperative risk scores or key process indicators. No factor categorised as 'organisational' is currently present in any perioperative risk score.

Conclusions: Through process mapping and a modified Delphi we gained insights into additional factors that may contribute to perioperative risk. Many were absent from currently used risk stratification scores. These results enable an appreciation of the contextual limitations of currently used risk tools and could support future research into the generation of more holistic datasets for the development of perioperative risk assessment tools.

Strengths and Weaknesses

- Novel use of process mapping to identify key perioperative stakeholders
- Multidisciplinary Delphi panel to gain breadth of perspective
- Performed across two sites

For peer review only

- Comprehensive results may be of use to other researchers designing perioperative research databases
- Results may be limited by low response rate in final round (although majority of consensus decisions made in round two)

Introduction

Understanding, predicting, and communicating risk is fundamental to perioperative practice [1]. The use of surgical risk stratification tools is a cornerstone of programs such as the National Emergency Laparotomy Audit (NELA) [2]. When using the output of such tools in day-to-day practice it is important to remember that they are developed from external electronic datasets and their application to decisions at the level of an individual centre or patient is complex and fraught with difficulty [3]. Risk scores commonly consist of patient and surgical factors that are associated with relevant outcomes [4]. However, performance of such scores is far from perfect [5]. This raises the question as to whether currently unmeasured factors may improve our understanding and prognostication of risk. There is strong evidence that this is the case. Unwarranted variation is widespread within the National Health Service (NHS), with between-centre differences leading to discrepancies in cost, efficiency, and most importantly, patient outcome [6]. Significantly, organisational or system factors have been shown in a recent analysis of NELA data to be associated with worse outcomes [7]. However, such factors do not feature in commonly used risk assessment tools

Healthcare is increasingly digitised with electronic health records (EHRs) capturing detailed events from across the hospital system. Perioperatively, EHRs may hold data pertaining to an individual's baseline state, operation details, physiological responses under anaesthesia, and perioperative complications. EHRs therefore appear to offer an appealing substrate to identify and test factors associated with perioperative outcome. In reality, due to the complexity of modern healthcare, the data they hold doesn't accurately capture the 'true' patient state but the record is in fact biased by the care processes involved in the recording of such data [8]. To identify nascent factors that may broaden our understanding of perioperative risk, including at the level of the healthcare system, it is thus vital that we understand how that care is delivered, and the affect this might have on the electronic record.

A system may be defined as a collection of elements serving a common purpose, with emergent behaviour arising through their interaction. Such systems may be considered as simple, complicated, or complex, with complex systems exhibiting certain key behaviours such non-linearity, non-scalability, and emergence [9]. Healthcare has been posited as such a complex system, which, perioperatively, may include pre-assessment clinics, operating theatres, staff from multiple disciplines, patients, equipment, consumables, and care processes. All of these may generate data which could provide novel insights into our understanding of risk. Researchers in other healthcare settings, where similar causal pathways may exist, have already recognised that standard statistical techniques may not be comprehensive enough to interrogate and understand such systems [10]. Therefore there is a need to consider additional strategies such as those used to understand other complex systems [11,12].

For peer review only

Our aim in this paper was to employ a systems approach [11] to develop a holistic dataset that a breadth of perioperative professionals felt captured all dimensions of perioperative risk. The output of such an endeavour would enable a rational approach to extracting information from EHRs for future research and enable a clearer understanding of how this data is captured as part of the wider care process. Such strategies, as well as offering a clearer scientific rationale for future hypothesis testing, could also encourage better data governance – by only extracting data fields from the EHR that were felt to be of clear importance.

To develop our consensus dataset, we employed a stepwise approach. Firstly, we sought to visualise how perioperative care is delivered through process mapping. This is a technique which enables complex systems to be visualised as a series of steps that has been used in various healthcare settings [13,14]. These process maps were then used to identify the range of perioperative professionals involved in patient care, and whose views we needed to capture. Using this list of 'stakeholders' we next conducted a modified Delphi [15] across two hospital sites to gain consensus on the breadth of factors at both patient, operation, and system level that were felt to impact on patient outcome.

Methods

Setting and approvals:

Participants were recruited from two UK hospital trusts utilising EHRs. Cambridge University Hospitals Trust (CUH) is a tertiary referral centre offering secondary and tertiary level surgical services to patients across the East of England. The West Suffolk Hospital (WSH) is a district general hospital based in Bury St Edmunds. WSH offers a range of secondary care services whilst referring patients for tertiary care to specialist centres including CUH. This study forms part of the 'Designing Improved Surgical Care for Older people' (DISCO) study, approved by the London and Surrey Borders Research Ethics Committee (reference 19/LO/1648). DISCO is jointly sponsored by the University of Cambridge and CUH.

Methodological approach and techniques:

Systems engineers use a variety of techniques to interrogate and design complex systems. A framework for applying these tools to healthcare has been recently jointly published by the UK's Royal Academy of Engineers, Royal College of Physicians, and Academy of Medical Sciences [11]. In this study we utilised brainstorming interviews and graphical elicitation [16], process mapping, and a Delphi process to ensure we captured the views of professionals (stakeholders) from across the perioperative system in two distinct sites. A project flow diagram demonstrating the sequential use of these techniques is shown in **Figure 1.**

Stakeholder identification and process mapping:

We formed a local steering group of experienced perioperative professionals. The group consisted of a consultant anaesthetist, geriatrician, and surgeon alongside a senior physiotherapist, occupational therapist, matron, and operations manager. Brainstorming interviews were conducted to identify stakeholder groups who should be represented on the Delphi panel. Interviews were structured around the iteration of process maps representing a stereotypical 'high-risk' surgical patient undergoing vascular surgery. Vascular surgery was chosen due to the ability to draw comparisons between elective and emergency cases as well as the need for clinical input from a range of perioperative professionals.

For peer review only

Stakeholders were identified from these maps and then chosen for representation on the Delphi panel if at least one member of the steering group felt this was appropriate.

Delphi panel formation:

Representatives were approached from each stakeholder group across both sites aiming for a complementary spread of sub-specialities between sites. Individuals were approached by lead researchers, provided with written information, and gave informed consent prior to each Delphi round.

Delphi structure – round one:

The Delphi consisted of three rounds and was distributed using an online survey tool (Qualtrics - www.qualtrics.com). In round one individuals were asked to provide free-text suggestions on what they felt *'Could contribute to a poor-outcome in an older patient undergoing surgery'.* We defined a poor-outcome to be where an individual lost their independence after surgery or suffered a complication (such as a myocardial infarction). Suggestions from the panel were combined with known important risk factors identified from a systematic review of perioperative risk scores [4]. The full list of scores is shown in **supplementary file 1** All risk factors (literature and panel) were voted on in the second and third rounds using a five-point Likert scale. Participants could also provide free-text comments and clarifications. A minority of questions in round two asked for specific free-text responses to expand on suggestions or provide relevant cut-offs (e.g. frailty score thresholds). New suggestions from round two were included for voting in round three. Before each major group of suggestions (e.g. 'comorbidities') respondents were asked to indicate on the same five point Likert scale how 'measurable' suggestions within this category were felt to be. This was to gauge the feelings of the panel on the practicality of this information being captured within an electronic record.

Delphi structure – rounds two and three:

All potential risk factors were presented for voting in a hierarchy consisting of groups and subgroups reflecting free-text suggestions. There were three broad domains - patient level (*that may have existed prior to an individual's current admission: e.g. comorbidities)*, admission level (*the circumstances and events occurring in any given admission*), and system level (*suggestions that were related to the structure or running of a service within a healthcare organisation*). Tracking of free text data for generating questionnaires was conducted using ATLAS.ti (www.atlasti.com) with quantitative analysis conducted in R v3.6.3 [17].

Definition of consensus:

Consensus was defined using criteria modified from a Delphi of quality indicators for patients with traumatic brain injury [18]. Given the relative heterogeneity of our panel, consensus for a given question required at least 50% of respondents to address it, a median score of >3.5, and an interquartile range (IQR) of ≤ 1. Consensus exclusion at the end of round two was stricter, requiring a score of <2.5, an IQR of ≤ 1, and no scores of 5 ('very important').

Comparing to common literature sources

At the conclusion of the Delphi all novel, consensus, suggestions were compared to a systematic review of perioperative structure and process indicators [19] as well as whether they had been examined in the statistical development of each risk score[20–33]. This approach was chosen to ensure that our final list of factors contained a solid core of important patient level factors whilst allowing us to critique novel findings against a comprehensive literature source.

Groupings, definitions, and results reporting.

Certain questions in the Delphi allowed participants to vote on cut-points (e.g. "*a 'high-risk' body mass index (BMI) is above 30*") or on an overarching concept that could encompass multiple factors (for instance 'complications' could encompass 'perioperative myocardial infarction' as well as surgical complications). For transparency all questions are presented in the **supplementary file 2** and included in relevant numerators or denominators within the results section. However, these cut-offs were not considered when reporting on factors present in literature risk scores unless they were explicitly mentioned (e.g. a risk score defined a specific cut-off).

Patient and public involvement in project:

The protocol for our project was reviewed by the CUH patient and public involvement panel in November 2017 and received favourable responses. Our project aims are clearly aligned with the James Lind associations priority setting partnership conducted with the National Institute of Academic Anaesthesia [34].

Results

System mapping.

Process maps representing the care of an elective and emergency vascular surgical patient were generated based on an understanding of local care pathways (abridged version in **Figure 2**, full examples as **figures 1 & 2 in supplementary file 1**). These diagrams were revised during brainstorming interviews with our steering group. Maps were used to identify relevant stakeholders across the perioperative pathway. In total, 52 unique staff groups were identified. Of these, 33 were nominated by at least one member of the steering group for representation on the Delphi panel (**Figure 3**).

Stakeholders and Delphi participants

Invitations to participate were sent to 91 professionals, identified by leads in both trusts (63 from CUH, 28 from WSH). These covered 23 broad professional groups as well as subspecialty expertise. Numbers of representatives are shown in **figure 3 in supplementary file 1**. Participants were able to contribute views from more than one perspective (e.g. anaesthetist AND intensive care doctor). Response rates ranged from 51% (*n=46*) in round one to 19% (*n = 17*) in round three. Conduct was significantly impacted by the coronavirus pandemic - distribution of the second and third rounds was delayed by six months due to wave one and the final round was completed as case pressures built prior to the second national UK lockdown in November 2020.

Minimum dataset: variables from the literature

For peer review only

25 risk scores were identified from a 2013 systematic review [19]. A full list of scores and their component variables are demonstrated in **table 1** and **figure 4** in **supplementary file 1.** Any of these variables not suggested in round one were included by default for voting in rounds two and three.

Delphi round one:

From the literature, 168 variables (representing specific measurements or characteristics) were identified (**figure 4** in **supplementary file 1** From free-text responses from participants, 411 suggestions for variables were identified, including 243 unique or refined definitions from those present in the literature. 80 of the 168 (48%) literature variables were not suggested by the panel in round one.

All suggestions were grouped into a framework separating out suggestions pertaining to the *patient*, their *admission,* and the *organisation* caring for them. For clarity of questioning, suggestions were further organised into related groups (such as comorbidities), sub-groups (e.g. cardiovascular comorbidities), and then a granular level that represented specific concepts or definitions (e.g. 'electrocardiogram changes' and 'left bundle branch block'). This structure is demonstrated in **Figure 4.** Distribution of new suggestions across these domains of *patient, admission, and organisation* is shown in **Figure 5A.** None of the suggestions that appeared to pertain to health system organisation or performance featured in currently used risk scores (**Figure 5A).**

Delphi rounds two and three:

In round two participants were able to vote on all levels of the questionnaire hierarchy (**Figure 4**) including groups and subgroups. In total 409 suggestions including 17 groups, 34 subgroups, and 358 variables (of which 117 were operationalised definitions of a variable - such as 'left bundle branch block' representing an 'important ECG change') were voted on. Two suggestions from the risk scores (cut-off values for defining polypharmacy) were held until round three as free-text suggestions for this threshold were sought from panellists in round two. Full details of all questions that were voted on were presented for voting across the final two rounds and are available as a **supplementary file 2**, with a summary shown in **Table 1**.

Having determined in round two that chronological age met the consensus criteria for inclusion, participants in round three were asked to suggest age thresholds that might be used to indicate a higher risk 'older' patient. From 19 participants, 16 gave a numeric threshold with the most common being 70 or older (*n = 6, 38%)*. Two participants highlighted that they felt an appropriate threshold would vary with the type of surgery and one, that they didn't believe chronological age was a valid criterion.

At the conclusion of round two, 357 (87%) of statements reached consensus criteria for inclusion. Analysis of free text suggestions identified a further 108 suggested variables for voting in the third Delphi round.

For peer review only

The median score for measurability in round two was four [IQR:3-4]. Laboratory results were felt to be the most measurable (attracting a median score of five) with patient *beliefs and behaviours*, *health service organisation, hospital performance & strain,* and *policies & procedures* attracting median scores of three.

For peer review only

Domain	Group	Example Variables	N	N (R2)	N (R3)	Consensus: any (N – [%])
Admission	Analgesic Plan	Analgesic plan | Multi modal analgesia | Opioid deprescribing pre-discharge | Opioid dose | Regional/epidural	5	5	0	5 [100]
Admission	Circumstances	Chest injuries | Delayed presentation | Emergency (vs elective) admission | Neck of femur fracture | Sepsis or organ failure on admission	12	8	4	11 [92]
Admission	Details of Surgery	BUPA Classification | Dutch Surgical Society Score | Indication for surgery (pathology) | Intraperitoneal, aortic, or intrathoracic operation | Length of Surgery	15	11	1	12 [80]
Admission	Discharge Planning	Delayed Transfer of Care | Discharge Planning | Needing care on discharge	3	3	0	3 [100]
Admission	Drug Management	Absence of a specific route of administration (e.g. oral) | Antiemetics & Parkinson's medications | Cytochrome P450 inhibitors/inducers | Drug Errors | Timing of drug reconcilliation (e.g. by pharmacy)	21	16	5	21 [100]
Admission	Intraoperative	Details of Surgery | Intraoperative Blood Loss | Intraoperative CO_2 Control | Night time operating | Operator Fatigue	27	22	4	26 [96]
Admission	Postoperative	Location and level of care | Multidisciplinary working | Number of attachments | Time to mobilisation | Ward moves/handovers	14	14	0	14 [100]
Admission	Postoperative Complications	Anastomotic Breakdown | Blood Transfusion | Cardiovascular Complications | Delirium | Fall	34	31	2	33 [97]
Admission	Preoperative	Appropriate preoperative ward | DNR status & Escalation plan | How long patients expect to stay in hospital | Multi-disciplinary working & communication |Use of a Risk Tool	25	22	3	25 [100]
Admission	Preoperative Physiology	Preoperative acid base status (e.g. pH, bicarbonate) | Preoperative Acute Renal impairment | Preoperative CVS impairment | Preoperative Delirium | Preoperative Electrolytes |	24	24	0	24 [100]
Organisation	Health Services Organisation	Availability of services (e.g. community therapy) | Practice type | Presence of a telephone advice service | Primary Care Structure | Tertiary admission/transfer	7	5	2	7 [100]
Organisation	Hospital Performance	Bed Occupancy Rates | Delayed Transfer of Care (DToC) Rates | ED Admission wait times | Intensive care occupancy | Number of vacant posts	11	4	6	10 [91]
Organisation	Policies and Procedures	Care Pathway Documents | Enhanced recovery programs | Procedures for minimising conflicts between teams | Procurement	6	5	1	6 [100]
Organisation	Staffing	Empathy | Enthusiasm and engagement | Expertise in the care of older people | Nursing cover | Training levels	13	11	2	13 [100]
Patient	Beliefs & Behaviours	Adoption of Sick role | Dependency on staff | Engagement with advice/treatment | Medication compliance | Patient motivation	12	9	2	11 [92]
Patient	Characteristics	Age | ASA score | Ethnicity | Genetics | Hearing or visual loss | Quality of Life	10	7	1	8 [80]

For peer review only

Patient	Comorbidities	(Fast) AF | BMI | Chronic Dysrhythmia | Diabetes | Symptoms that limit eating	168	99	48	146 [87]
Patient	Frailty	Deconditioning | Frailty score | Frailty Syndrome: Delirium | Frailty Syndrome: Falls | Frailty Syndrome: Incontinence | Loss of muscle	19	6	7	13 [68]
Patient	Function	Able to drive | Able to use stairs | Continent | Defined by ADLs | Independent with transfers and walking	17	14	2	16 [94]
Patient	Home Circumstances	Access difficulties (e.g. steps) | Accomodation type | Bathroom access | Bedroom access | Can the house support single storey living?	40	22	15	37 [93]
Patient	Lab Results	Albumin | ALT | APTT | AST | Bilirubin | Cholesterol | CK | Glucose | Haematocrit | Haemoglobin | Lab Results | Lymphocytes | Nucleated RBC | Platelets | PT | Thrombocytopenia | Transferrin | Transthyretin | Triglycerides | WCC	21	13	2	15 [71]
Patient	Longterm medications	Antiplatelets | Diuretics | DOACs (Direct Oral Anticoagulants) | Immunosuppressants | Number of medications | Warfarin	15	7	5	12 [80]

Table 1: Summary of findings from a multi-site Delphi of perioperative professionals to identify variables of importance in determining or predicting outcome after surgery. Domain and Group refer to section of Delphi questionnaire, a subset of example variables considered within these sections are shown for clarity (full details available in supplementary material). *N = total number of variables / thresholds considered by praticipants in each section, N(Rx) = number reaching consensus inclusion in round two or three, Consensus (any) = Number reaching consensus [%] across any round. AF = Atrial fibrillation, ALT = Alanine transferase, APTT = activated partial thromboplastin, time, ASA = American Society of Anesthesiologists Score, AST = Aspartate Transferase, BMI = Body Mass Index, British United Provident Association, CK = Creatinine Kinase, CVS = Cardiovascular system, DNR = Do not resuscitate, GP = General Practice, PT = Prothrombin Time, WCC = White Cell Count,*

For peer review only

In the third Delphi round, 110 of 158 (70%) variables met the consensus criteria for inclusion, one variable (*"shortness of breath on strenuous exercise"*) met the criteria for exclusion. In total, across both rounds, 468 of 519 (90%) suggestions that were presented to the panel reached consensus decisions (**Figure 5B**). In round three median measurability was put at four [IQR:4-5] with only *beliefs & behaviours* and *health service organisation* attracting median scores of less than four. Across both latter rounds all of the suggestions encompassing health system organisation reached the definition for consensus.

Comparison to literature variables

To assess whether variables might have been previously examined but excluded from published risk scores due to a lack of statistical significance the original papers describing each were examined [20–33]. 25 (7%) of the 351 unique suggestions from rounds one and two had been previously examined at an earlier phase in the development of at least one risk tool (details in **Supplementary file 2**).

All novel suggestions were also compared against a list of process and structure indicators from a 2018 systematic review [7]. 111 of 352 (32%) novel suggestions could be mapped against one or more metrics identified in this paper. When new suggestions defining patient level characteristics (e.g., comorbidities) were excluded - 88 of 161 (54%) new suggestions relating to admission circumstances or organisational function were represented. Variables that did not appear included markers of system performance (e.g., *number of vacant posts, delayed transfer of care rates)* and staffing (e.g., *occupational therapy cover*) as well as examples of postoperative complications (e.g., *anastomotic breakdown*). Full details can be seen in the **supplementary file 2.**

Discussion

This study demonstrates the views of multidisciplinary clinicians on factors felt to influence perioperative risk. We feel that both our findings and methods will be of interest to researchers, clinicians, data-scientists, and managers. Our results highlighted that factors related to in-hospital events, organisational structure, and hospital performance were felt to be important. Such suggestions were conspicuously absent from commonly used perioperative risk scores **(Figure 5A)** [4] but are compatible with work demonstrating inter-centre variation in outcome [34]. Our final list of variables (available in **supplementary file 2**, summarised in **Table 1**) is likely to be of use to researchers in the field seeking to intelligently curate data from their own EHRs.

We hypothesised that process mapping may enable us to identify a panel of stakeholders whose expertise captured all facets of perioperative care and, that in doing so, we may gain novel insights. This approach is at least partially vindicated in that most factors voted on (351/519 – 68%) were suggested by the panel rather than in our selected subset of risk scores. We acknowledge that it is possible that some of our suggestions may feature in other risk scores. We required a sufficiently broad baseline to assure the validity of our results whilst critiquing the scale of any novel insights. Given the rising popularity of risk scores, we did however require a pragmatic approach. Therefore, we selected scores (including those

For peer review only

commonly used in UK practice such as P-POSSUM) [35] that had been validated in distinct surgical populations.

The importance of 'non-patient' level factors in prognostication has not been thoroughly explored, perhaps reflecting the challenges in recording such concepts electronically. When considering quantitative data from EHRs, improved risk assessment can be seen by incorporating implicit information such as the timing of blood samples, as well as their resulting value [36]. Here, this timing information is presumably recognising a potentially otherwise undocumented recognition of a clinically unwell patient by a diligent clinician taking, and interpreting, blood samples out of hours. The results of this study suggest that variables not captured in a hospital EHR, such as detailed aspects of social circumstances, may be important. A machine learning model containing only 'sociomarkers' (derived from place of residence) has yielded comparable performance in the prediction of childhood asthma exacerbation compared to the use of patient-level data [37]. However, conflicting findings have been shown in other settings. Although qualitative data derived from patient interviews has suggested that social support and psychological state are influences on individual readmissions with heart failure [38,39], small studies looking to incorporate questionnaire derived markers have failed to demonstrate improved prognostication [40] despite the apparent use of 'unstructured' free-text data in another study [41]. Perioperatively it is well known that clinician judgement improves the performance of commonly utilised risk tools such as SORT (Surgical Outcome Risk Tool) [41]. What is unclear is what additional factors this clinician judgement encapsulates and whether it may reflect an appreciation or implicit judgement of some of the factors identified in this study.

We feel that a strength of our study has been to identify avenues for future research, by providing a comprehensiv list of social and health system factors that researchers may want to explore in future datasets. The documented differences in outcome between institution[42], socioeconomic strata[42], and our findings mean that exploring these factors warrants further investigation. One approach to try and address this may be to broaden the definition as to what we view as constituting 'healthcare data'. The Institute of Medicine suggests that relevant ancillary data sources may include human resources records and patient complaints [43]. This could conceivably aid with the incorporation of factors such as staffing level, with complaints data, if adequately coded using tools such as the Healthcare Complaints Analysis Tool (HCAT), identifying poor staff-patient relationships and communication [44]. However, we acknowledge the challenges (both logistical and governance) in formulating linked datasets capable of answering these questions, especially at significant scale.

However, investigation of these factors may yield crucial insights. Firstly, for predictive analyses, an appreciation of system factors in influencing patient-level risk could aid in reducing uncertainty around calculated predictions, whilst providing crucial contextual information for shared decision making. Increasing the breadth of future datasets obviously raises the need to ensure that developed predictive models are suitably parsimonious. Secondly, a comprehensive dataset containing these novel variables could form a substrate for causally focused analyses. When conducting such an analysis, the generation of a causal model should proceed in a data-naïve manner, to ensure adequate recognition of latent variables with potential causal effects [45]. Our results could be starting point for such work, by

For peer review only

highlighting variables of potential causal importance that would be missed with data-driven approaches on currently existing datasets.

This study took the unique step of drawing on techniques from systems engineering to structure a Delphi survey of professionals. Our methods will be of direct relevance to those involved in informatics or quality improvement work. A systems approach is a way of addressing problems holistically, aware of the interaction between elements and subsequent unexpected behaviour[11,46]. Suggestions from the panel related to the circumstances of admission, hospital performance, and external pressures. A conceptual framework is thus that the ultimate outcome of any patient stay depends on the interaction between factors at different levels. Conceptually this hierarchical agreement is appealing, with the importance of external factors, such as coronavirus pressures, a key part of current daily practice. Beyond our unique methodological approach and multidisciplinary nature of our results, a further point of originality within our work is that the Delphi panel were specifically asked to suggest factors that could result in a loss of independence on discharge, a key concern of patients undergoing surgery [47]. Despite this it is not widely considered in perioperative risk stratification[4].

We do however acknowledge the fall off in response rate across rounds. This arose due to the significant pressures of the coronavirus pandemic and that many participants on our panel were undertaking additional clinical duties. We would counter this by highlighting the breadth of specialties surveyed, and that the response rate in round two was 38%, and that a majority of consensus factors (357/490) were identified in this round. It is possible that the low response rate in round three skewed the remaining factors towards reaching consensus, but this should make our resultant list of variables especially sensitive (albeit potentially less specific). The high rates of consensus could also reflect true strength of feeling but may have arisen due to issues with questionnaire length, panel composition, and response rates.

Conclusion

This study demonstrates the feasibility of using systems engineering tools to identify and engage clinicians in identifying factors felt to impact on patient relevant outcomes after surgery. The results themselves highlight that these professionals identify non-patient level factors as modulators of perioperative risk. Further work is needed to prioritise these results to develop electronic surrogates to validate their significance in real datasets.

Contributorship:

DJS: Conception, Delphi design, participant recruitment, analysis, primary manuscript drafting, corresponding author, approval of final version, agreement to be accountable for all aspects of the work

PJC: Conception, Delphi design, critical revisions to manuscript, approval of final version, agreement to be accountable for all aspects of the work

NL: Site specific recruitment and set-up, Manuscript drafting, Critical revisions to manuscript, approval of final version, agreement to be accountable for all aspects of the work

For peer review only

TB: Analytical advice, Delphi revisions, Critical revisions to manuscript, approval of final version, agreement to be accountable for all aspects of the work

FJG: Stakeholder identification, critical revisions to manuscript, approval of final version, agreement to be accountable for all aspects of the work

BN: Site specific recruitment, critical revisions to manuscript, approval of final version, agreement to be accountable for all aspects of the work

AE: Conception, Delphi design, critical revisions to the manuscript, approval of final version, agreement to be accountable for all aspects of the work

PJC and NL both contributed equally to this work and should be considered joint senior author

Ethical approvals

The project was approved by the London and Surrey Borders REC reference: 19/LO/1648

Funding statement:

This research was funded, in whole or in part, by the Wellcome Trust, Grant number: 220542/Z/20/Z (to DJS). A CC BY or equivalent licence is applied to the Author Accepted Manuscript (AAM) arising from this submission, in accordance with the grant's open access conditions.

This research was supported by the NIHR Cambridge Biomedical Centre (BRC 1215 20014). The views and opinions expressed by authors in this publication are those of the authors and do not necessarily reflect those of the NHS, the NIHR, or the department of health.

Data Sharing Statement. Summary results for each round of our study are available in the supplementary material.

Competing Interests Statement: No competing interests declared

References:

1 General Medical Council. Decision making and consent. 2020. https://www.gmc-uk.org/-/media/documents/gmc-guidance-for-doctors---decision-making-and-consent-english_pdf-84191055.pdf (accessed 30 Nov 2021).

2 Nela. NELA Risk Calculator. https://data.nela.org.uk/riskcalculator/

3 Lewis G. Next Steps for Risk Stratification in the NHS. NHS England 2015. https://www.england.nhs.uk/wp-content/uploads/2015/01/nxt-steps-risk-strat-glewis.pdf (accessed 14 Dec 2021).

For peer review only

4 Moonesinghe SR, Mythen MG, Das P, *et al.* Risk stratification tools for predicting morbidity and mortality in adult patients undergoing major surgery: qualitative systematic review. *Anesthesiology* 2013;**119**:959–81.

5 Kumar A, Karmarkar A, Downer B, *et al.* Current Risk Adjustment and Comorbidity Index Underperformance in Predicting Post-Acute Utilization and Hospital Readmissions After Joint Replacements: Implications for Comprehensive Care for Joint Replacement Model. *Arthritis Care Res* 2017;**69**:1668–75.

6 Lord Carter of Coles. Operational productivity and performance in English NHS acute hospitals: Unwarranted variations. Department of Health 2016. https://assets.publishing.service.gov.uk/government/uploads/system/uploads/attachment_data/file/499229/Operational_productivity_A.pdf (accessed 7 Dec 2021).

7 Oliver CM, Bassett MG, Poulton TE, *et al.* Organisational factors and mortality after an emergency laparotomy: multilevel analysis of 39 903 National Emergency Laparotomy Audit patients. *Br J Anaesth* 2018;**121**:1346–56.

8 Hripcsak G, Albers DJ. Next-generation phenotyping of electronic health records. *J Am Med Inform Assoc* 2013;**20**:117–21.

9 Ladyman J, Lambert J, Wiesner K. What is a complex system? *Eur J Philos Sci* 2013;**3**:33–67.

10 Stronks K, Nicolaou M. Embracing complexity in social epidemiology. *The Lancet Public Health* 2018;**3**:e352–3.

11 Clarkson PJ, Bogle D, Dean J, *et al. Engineering better care: a systems approach to health and care design and continuous improvement*. London: : Royal Academy of Engineering 2017.

12 Rutter H, Savona N, Glonti K, *et al.* The need for a complex systems model of evidence for public health. *Lancet* 2017;**390**:2602–4.

13 Forrester JA, Koritsanszky LA, Amenu D, *et al.* Developing Process Maps as a Tool for a Surgical Infection Prevention Quality Improvement Initiative in Resource-Constrained Settings. *J Am Coll Surg* 2018;**226**:1103-1116.e3.

14 Antonacci G, Reed JE, Lennox L, *et al.* The use of process mapping in healthcare quality improvement projects. *Health Serv Manage Res* 2018;**31**:74–84.

15 Abbott TEF, Fowler AJ, Pelosi P, *et al.* A systematic review and consensus definitions for standardised end-points in perioperative medicine: pulmonary complications. *Br J Anaesth* 2018;**120**:1066–79.

16 Crilly N, Blackwell AF, Clarkson PJ. Graphic elicitation: Using research diagrams as interview stimuli. *Qual Res* 2006;**6**:341–66.

17 R Core Team. *R: A Language and Environment for Statistical Computing*. Vienna, Austria: : R Foundation for Statistical Computing 2013.

18 Huijben JA, Wiegers EJA, De Keizer NF, *et al.* Development of a quality indicator set to measure and

For peer review only

improve quality of ICU care for patients with traumatic brain injury. *Crit Care* 2019;**23**:95.

19 Chazapis M, Gilhooly D, Smith AF, *et al.* Perioperative structure and process quality and safety indicators: a systematic review. *Br J Anaesth* 2018;**120**:51–66.

20 Detsky AS, Abrams HB, Forbath N, *et al.* Cardiac Assessment for Patients Undergoing Noncardiac Surgery: A Multifactorial Clinical Risk Index. *Arch Intern Med* 1986;**146**:2131–4.

21 Donati A, Ruzzi M, Adrario E, *et al.* A new and feasible model for predicting operative risk. *Br J Anaesth* 2004;**93**:393–9.

22 Hadjianastassiou VG, Tekkis PP, Poloniecki JD, *et al.* Surgical Mortality Score: Risk Management Tool for Auditing Surgical Performance. *World J Surg* 2004;**28**:193–200.

23 Haga Y, Ikei S, Ogawa M. Estimation of Physiologic Ability and Surgical Stress (E-PASS) as a New Prediction Scoring System for Postoperative Morbidity and. *Surgery Today* 1999;**29**:219–25.

24 Guigoz Y, Vellas B. The Mini Nutritional Assessment (MNA) for grading the nutritional state of elderly patients: presentation of the MNA, history and validation. *Nestle Nutr Workshop Ser Clin Perform Programme* 1999;**1**:116–22.

25 Nathanson BH, Higgins TL, Kramer AA, *et al.* Subgroup mortality probability models: Are they necessary for specialized intensive care units? *Crit Care Med* 2009;**37**:2375–86.

26 Pillai SB, Van Rij AM, Williams S, *et al.* Complexity- and risk-adjusted model for measuring surgical outcome. *British Journal of Surgery* 1999;**86**:1567–72.

27 Story DA, Fink M, Leslie K, *et al.* Original Papers Perioperative mortality risk score using pre- and postoperative risk factors in older patients. *Anesthesia and Intensive Care* 2009;**37**:392–8.

28 Scott S, Lund JN, Gold S, *et al.* An evaluation of POSSUM and P-POSSUM scoring in predicting post-operative mortality in a level 1 critical care setting. *BMC Anesthesiology* 2014;**14**:1–7.

29 Mercaldo ND, Jacques PJS, D M. Expansion of the Surgical Apgar Score across All Surgical Subspecialties as a Means to Predict Postoperative Mortality. *Anesthesiology* 2011;**114**:1305–12.

30 Lee TH, Marcantonio ER, Mangione CM, *et al.* Derivation and prospective validation of a simple index for prediction of cardiac risk of major noncardiac surgery. *Circulation* 1999;**100**:1043–9.

31 Gil Cebrian J, Bello Cámara MP, Diaz-Alersi R. Apache Ii. *Intensive Care Med* 1987;**13**:143.

32 Gawande AA, Kwaan MR, Regenbogen SE, *et al.* An Apgar Score for Surgery. *J Am Coll Surg* 2007;**204**:201–8.

33 Prytherch DR, Sirl JS, Weaver PC, *et al.* Towards a national clinical minimum data set for general surgery. *Br J Surg* 2003;**90**:1300–5.

34 NIAA, The James Lind Alliance. Anaesthesia and Perioperative Care Priority Setting Partnership: most popular questions. 2015.https://www.niaa.org.uk/PSP (accessed 10 Aug 2022).

35 Agniel D, Kohane IS, Weber GM. Biases in electronic health record data due to processes within the

For peer review only

healthcare system: retrospective observational study. *BMJ* 2018;**361**:k1479.

36 Shin EK, Mahajan R, Akbilgic O, *et al.* Sociomarkers and biomarkers: predictive modeling in identifying pediatric asthma patients at risk of hospital revisits. *npj Digital Medicine* 2018;**1**:1–5.

37 Retrum JH, Boggs J, Hersh A, *et al.* Patient-identified factors related to heart failure readmissions. *Circ Cardiovasc Qual Outcomes* 2013;**6**:171–7.

38 Krumholz HM, Chaudhry SI, Spertus JA, *et al.* Do Non-Clinical Factors Improve Prediction of Readmission Risk?: Results From the Tele-HF Study. *JACC Heart Fail* 2016;**4**:12–20.

39 Ahmad FS, French B, Bowles KH, *et al.* Incorporating patient-centered factors into heart failure readmission risk prediction: A mixed-methods study. *Am Heart J* 2018;**200**:75–82.

40 Watson AJ, O'Rourke J, Jethwani K, *et al.* Linking electronic health record-extracted psychosocial data in real-time to risk of readmission for heart failure. *Psychosomatics* 2011;**52**:319–27.

41 Wong DJN, Harris S, Sahni A, *et al.* Developing and validating subjective and objective risk-assessment measures for predicting mortality after major surgery: An international prospective cohort study. *PLoS Med* 2020;**17**:e1003253.

42 Wan YI, McGuckin D, Fowler AJ, *et al.* Socioeconomic deprivation and long-term outcomes after elective surgery: analysis of prospective data from two observational studies. *Br J Anaesth* 2021;**126**:642–51.

43 Aspden P, Corrigan JM, Wolcott J, *et al. Patient Safety:: Achieving a New Standard for Care Editors, Committee on Data Standards for Patient Safety*. 2004.

44 Gillespie A, Reader TW. The Healthcare Complaints Analysis Tool: Development and reliability testing of a method for service monitoring and organisational learning. *BMJ Qual Saf* 2016;**25**:937–46.

45 Tennant PWG, Murray EJ, Arnold KF, *et al.* Use of directed acyclic graphs (DAGs) to identify confounders in applied health research: review and recommendations. *Int J Epidemiol* 2021;**50**:620–32.

46 Komashie A, Ward J, Bashford T, *et al.* Systems approach to health service design, delivery and improvement: a systematic review and meta-analysis. *BMJ Open* 2021;**11**:e037667.

47 Ladha KS, Wijeysundera DN. Role of patient-centred outcomes after hospital discharge: a state-of-the-art review. *Anaesthesia* 2020;**75 Suppl 1**:e151–7.

For peer review only

Figure Legends

Figure 1: Project stages. Process mapping was performed with a steering group of experienced professionals, with maps iterated between brainstorming interviews. At the conclusion of this stage two outputs were generated – the maps themselves and a stakeholder diagram showing the viewpoints we sought to capture in our Delphi. The Delphi was conducted across three rounds with the first seeking free-text suggestions from the panel. These were combined with risk factors featuring in a systematic review of common perioperative risk tools [4]. All suggestions were then voted on by the panel across two further rounds to gain consensus. *N*= number of risk factors gained from each source. Our final consensus list of risk factors is available in **supplementary material.**

Figure 2: Simplified process map of a patient referred for elective vascular surgery at our institution. *Blue* boxes highlight preoperative care, *green* intraoperative care, *orange* postoperative care. Boxes indicate processes, diamonds indicate decision points. Dashed lines indicate boundary between community and hospital. Stakeholders (e.g. Anaesthetist) are shown in bold text. Lower panel highlights more granular view of numbered processes including revisions to initial diagram following discussions with a steering group of perioperative professionals. Fully granular maps for elective and emergency pathways are available in the supplementary material. *OIR = overnight intensive recovery (level 2 post anaesthesia care unit for high risk patients).*

Figure 3: Stakeholder diagram: Stakeholder groups identified for representation on a Delphi panel to understand what defines a high-risk surgical patient and impacts on their outcome. All groups were identified by a collaborative process mapping exercise with a steering group of key perioperative professionals. Diagram template adapted from that available at www.iitoolkit.com, used with permission. *ED = Emergency Department, HCA = Health-care assistant, OT = Occupational therapist, PAU = Pre-assessment unit, PACU = Post-anaesthesia care unit*

Figure 4: Delphi structure showing main risk factor groups identified through participant suggestions and the literature. The questionnaire enabled people to vote on three broad domains (Patient, Admission, Organisation) and relevant groups (black text in lozenges). Many of these groups had relevant subgroups (white text). Participants could also vote on individual date elements (variables or their definitions) within each of these. Full list of these are available in the supplementary material.

Figure 5: Representation of the Delphi questionnaires as a dendrogram, demonstrating hierarchical organisation of questions. Colours represent broad domains of statements being voted on by the panel; pertaining to a patient, circumstances of admission, or those pertaining to the organisation caring for them. Final questions that were voted on are represented by terminal leaves. Branch points

For peer review only

represent questionnaire structure. In panel **A**, expanded nodes represent those variables derived from a systematic review of commonly used perioperative risk scores. In panel **B,** expanded nodes represent those that reached criteria for consensus inclusion in a dataset seeking to understand drivers of perioperative risk in older people.

For peer review only

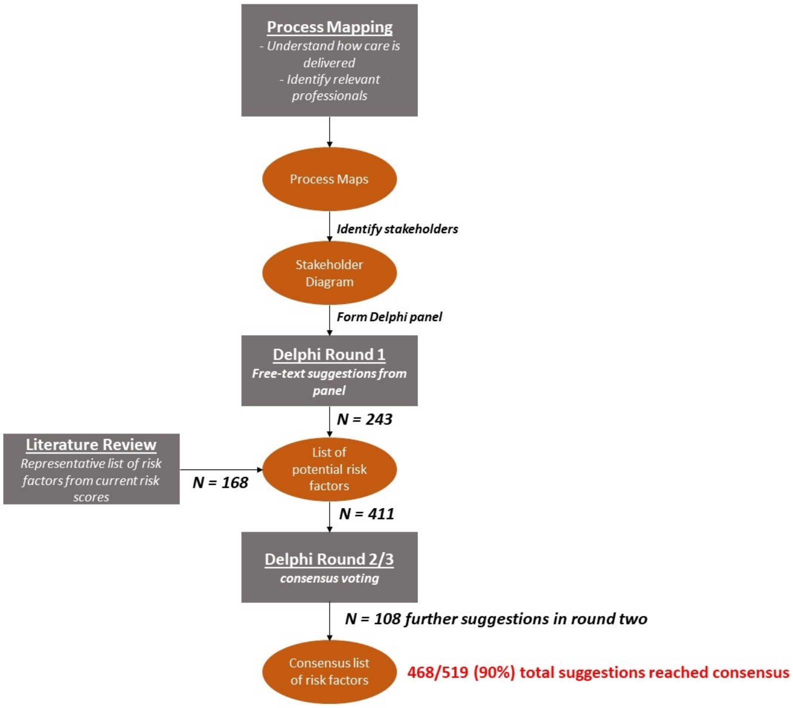

Figure 1: Process mapping was performed with a steering group of experienced professionals, with maps iterated between brainstorming interviews. At the conclusion of this stage two outputs were generated – the maps themselves and a stakeholder diagram showing the viewpoints we sought to capture in our Delphi. The Delphi was conducted across three rounds with the first seeking free-text suggestions from the panel. These were combined with risk factors featuring in a systematic review of common perioperative risk tools [4]. All suggestions were then voted on by the panel across two further rounds to gain consensus. N= number of risk factors gained from each source. Our final consensus list of risk factors is available in supplementary material.

355x266mm (96 x 96 DPI)

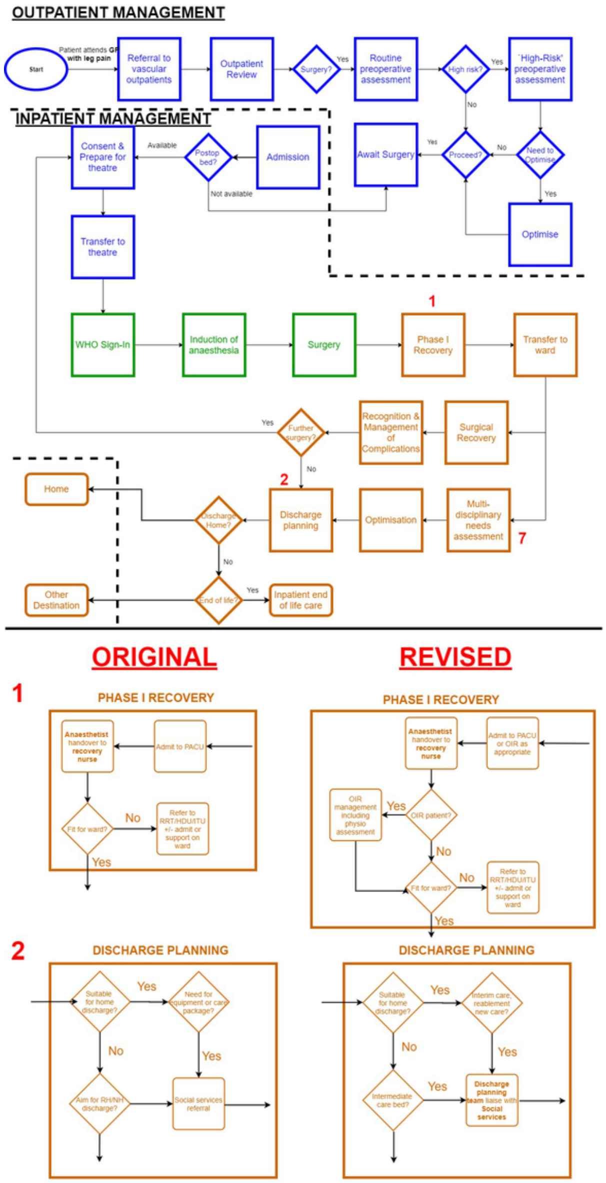

Figure 2: Simplified process map of a patient referred for elective vascular surgery at our institution. Blue boxes highlight preoperative care, green intraoperative care, orange postoperative care. Boxes indicate processes, diamonds indicate decision points. Dashed lines indicate boundary between community and hospital. Stakeholders (e.g. Anaesthetist) are shown in bold text. Lower panel highlights more granular view of numbered processes including revisions to initial diagram following discussions with a steering group of perioperative professionals. Fully granular maps for elective and emergency pathways are available in the supplementary material. OIR = overnight intensive recovery (level 2 post anaesthesia care unit for high risk patients).

49x98mm (300 x 300 DPI)

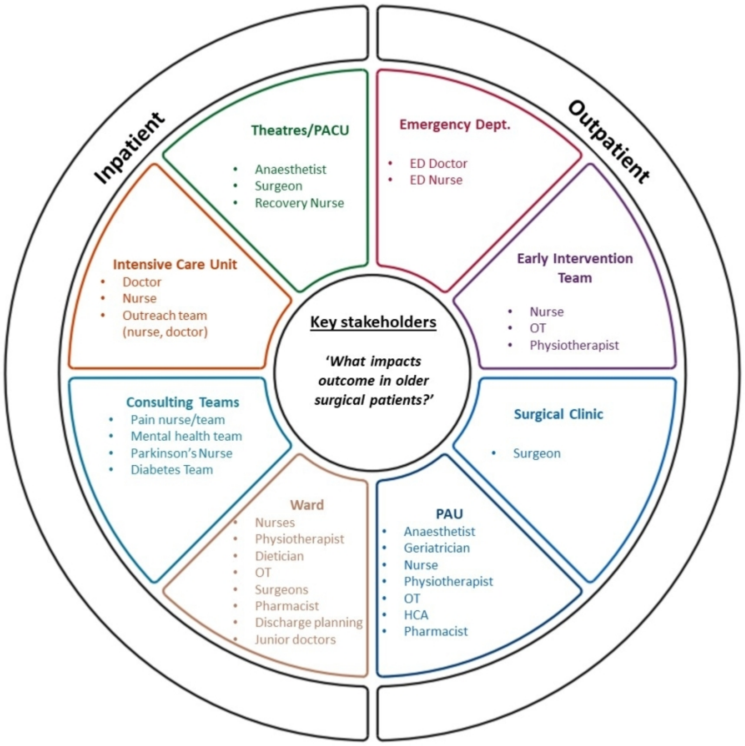

Figure 3: Stakeholder diagram: Stakeholder groups identified for representation on a Delphi panel to understand what defines a high-risk surgical patient and impacts on their outcome. All groups were identified by a collaborative process mapping exercise with a steering group of key perioperative professionals. Diagram template adapted from that available at www.iitoolkit.com, used with permission. ED = Emergency Department, HCA = Health-care assistant, OT = Occupational therapist, PAU = Pre-assessment unit, PACU = Post-anaesthesia care unit

355x266mm (96 x 96 DPI)

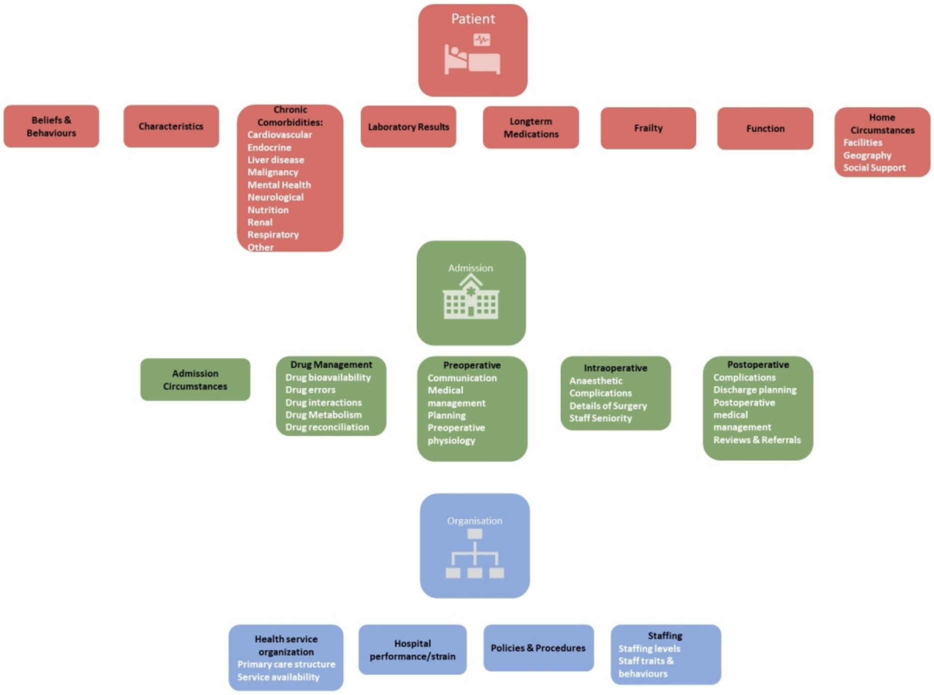

Figure 4: Delphi structure showing main risk factor groups identified through participant suggestions and the literature. The questionnaire enabled people to vote on three broad domains (Patient, Admission, Organisation) and relevant groups (black text in lozenges). Many of these groups had relevant subgroups (white text). Participants could also vote on individual date elements (variables or their definitions) within each of these. Full list of these are available in the supplementary material.

474x339mm (96 x 96 DPI)

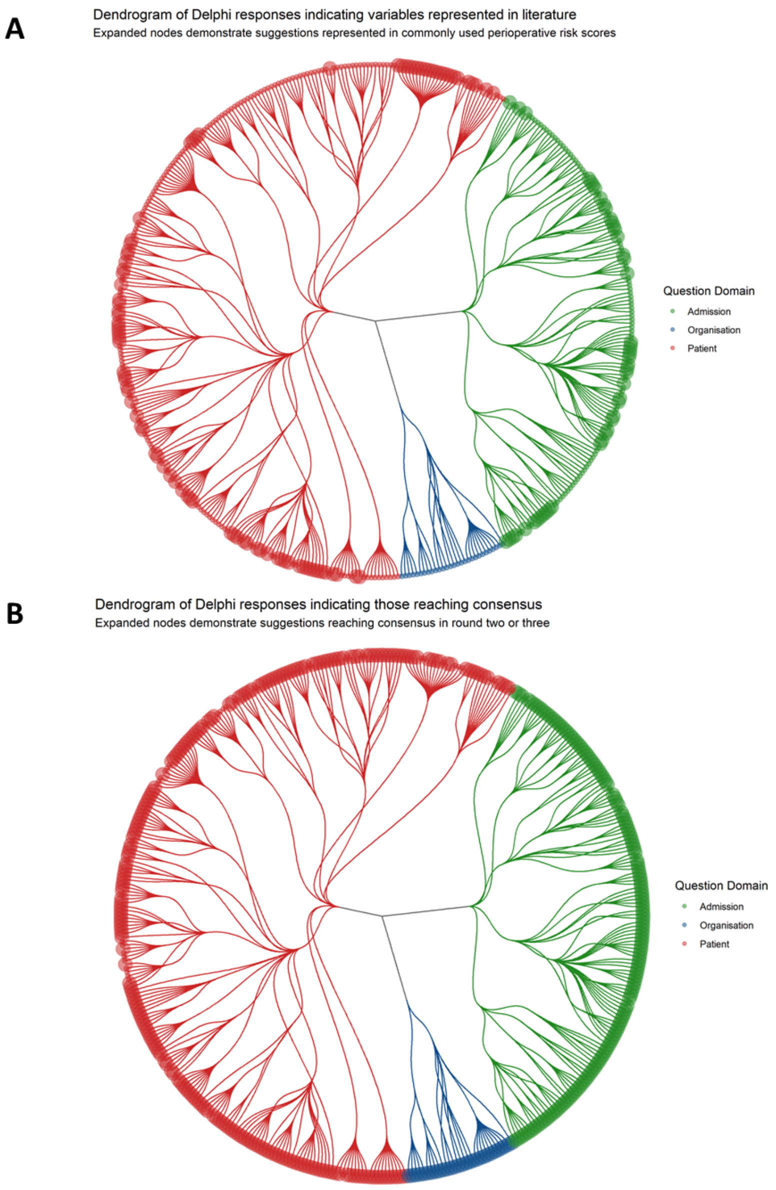

Figure 5: Representation of the Delphi questionnaires as a dendrogram, demonstrating hierarchical organisation of questions. Colours represent broad domains of statements being voted on by the panel; pertaining to a patient, circumstances of admission, or those pertaining to the organisation caring for them. Final questions that were voted on are represented by terminal leaves. In panel A, expanded nodes represent those variables derived from a systematic review of commonly used perioperative risk scores. In panel B, expanded nodes represent those that reached criteria for consensus inclusion in a dataset seeking to understand drivers of perioperative risk in older people.

266x355mm (96 x 96 DPI)

Can process mapping and a multi-site Delphi of perioperative professionals inform our understanding of system-wide factors that may impact operative risk?

Stubbs DJ, Bashford T, GIlder FJ, Nourallah B, Ercole A, Levy N, Clarkson PJ

Supplementary Material:

This document contains supplementary figures, **further material is available as:**

1. A **.xlsx format** spreadsheet showing all suggestion voted on across the three rounds of the Delphi

Contents of this document

1. **Annotated process maps demonstrating the care of elective (a) and emergency (b) vascular surgical patients**
2. **List of risk scores used to provide a 'minimum' dataset for voting on in Delphi round two**
3. **Component variables from these risk scores**
4. **Hierarchy of question groupings used in Delphi rounds two and three**
5. **'Novel' variables from the Delphi**

For peer review only

Supplementary Figure 1: Process map of elective vascular care used to identify perioperative stakeholders.

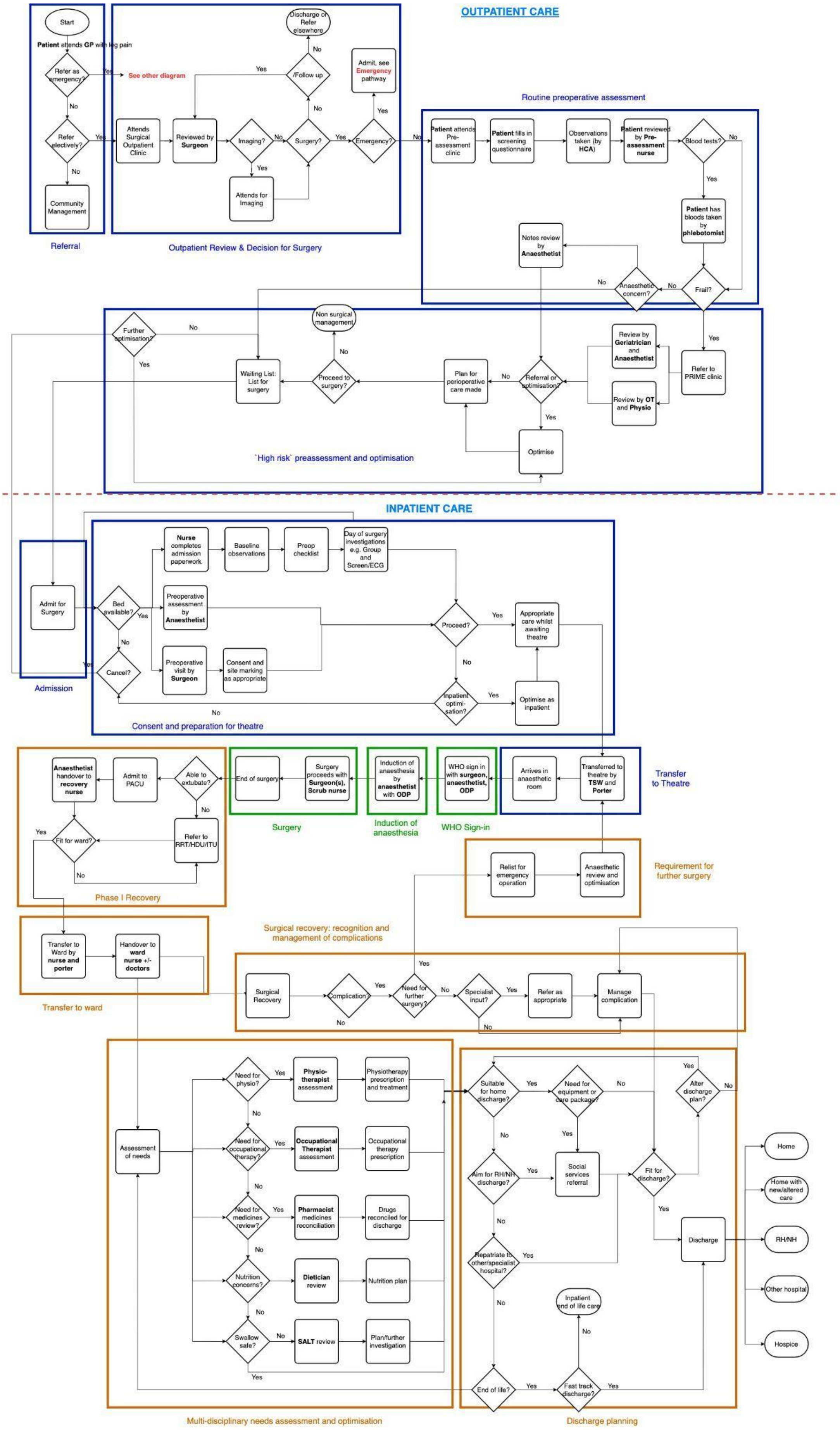

Supplementary Figure 2: Process map of emergency vascular care used to identify perioperative stakeholders. \

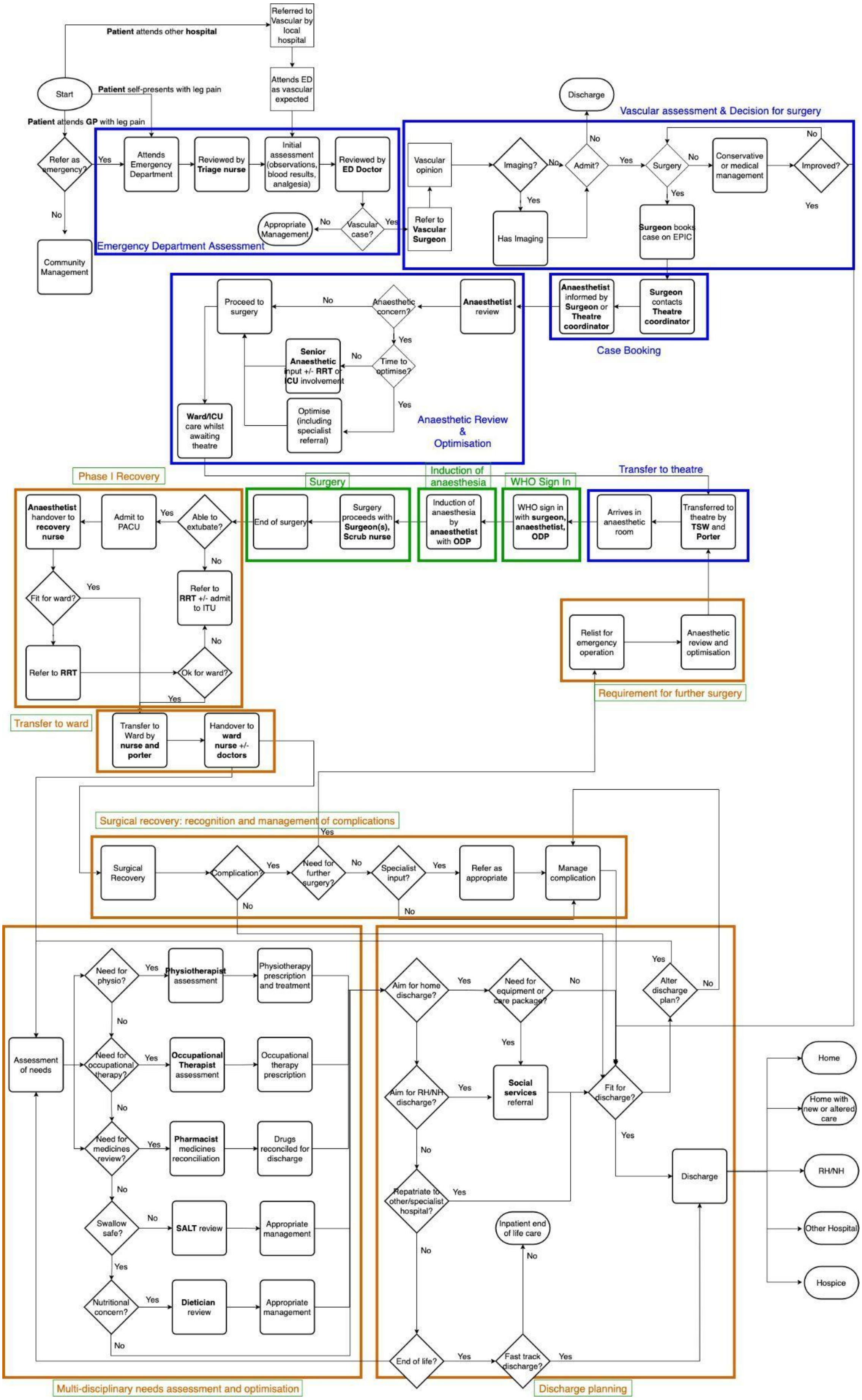

(In both Figure S1 and S2, coloured boxes correspond to abridged care processes shown in Figure 1 in main text)

Risk scores used in formation of a 'minimum' dataset for voting in rounds two and three

**ASA score**
**APACHE**
**BHOM**
**Charlson Comorbidity Index**
**DELAWARE**
**Detsky index**
**E-PASS**
**Edmonton Frail scale**
**ICISS**
**IRIS**
**Maastricht Nutritional Assessment**
**Mini nutritional assessment**
**MPM III**
**Nutritional risk index**
**Otago Surgical outcome score**
**Perioperative Mortality Risk Score**
**POSSUM**
**P-POSSUM**
**RCRI**
**SAPS II**
**Subjective Global assessment**
**Surgical Apgar**
**Surgical mortality score**
**Surgical Risk Scale**
**Surgical Risk Score (Donati)**

Supplementary Table 1: List of risk scores identified as having been validated in heterogeneous surgical cohorts in a 2013 systematic review[1]. These scores were deconstructed (see **Supplementary Figure 3**) to provide the minimum components of any dataset resulting from the Delphi. *ASA = American Society of Anesthesiologists, BHOM – Biochemistry & Haematology Outcome Model, APACHE = Acute physiology & chronic health evaluation, DELAWARE = Dense Laboratory Whole blood applied Risk Estimation, E-PASS = Estimation of physiological ability and surgical stress, ICISS = ICD injury severity score, IRIS = Identification of risk in surgical patients score, MPM III = Mortality prediction model (III), POSSUM = Physiological and Operative Severity Score for the enUmeration of Mortality and morbidity, P-POSSUM = Portsmouth modification of POSSUM, RCRI = Revised cardiac risk index, SAPS II = Simplified acute physiology score II*

1. Moonesinghe SR, Mythen MG, Das P, Rowan KM, Grocott MP. Risk stratification tools for predicting morbidity and mortality in adult patients undergoing major surgery: qualitative systematic review. *Anesthesiology* 2013; **119**: 959–81.

For peer review only

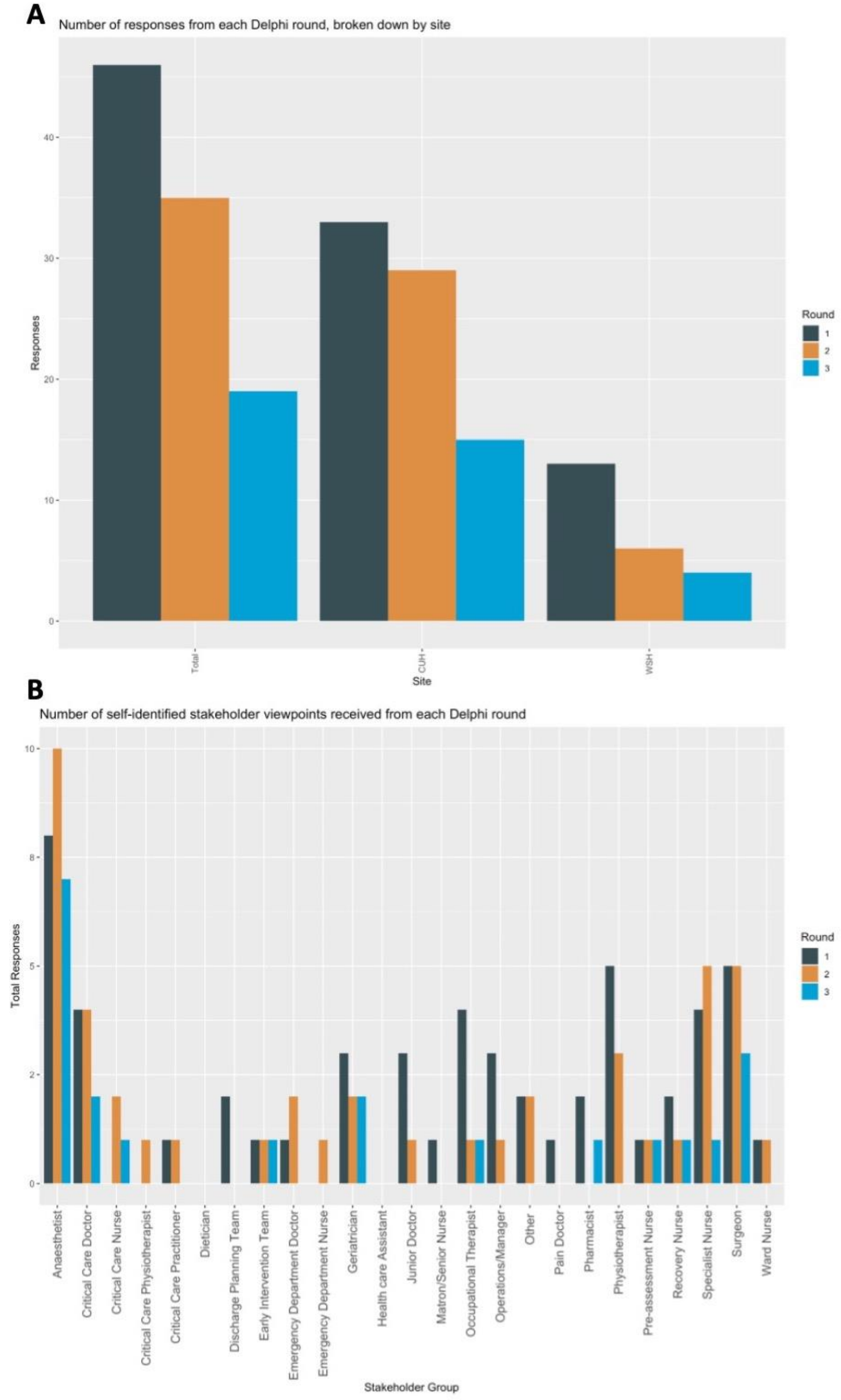

Supplementary Figure 3: Response rate and respondent profile by round for a Delphi exercise to gain a consensus view on factors impacting on outcome in older surgical patients. *CUH = Cambridge University Hospitals (a tertiary care centre), WSH = West Suffolk Hospital (a secondary care centre)*

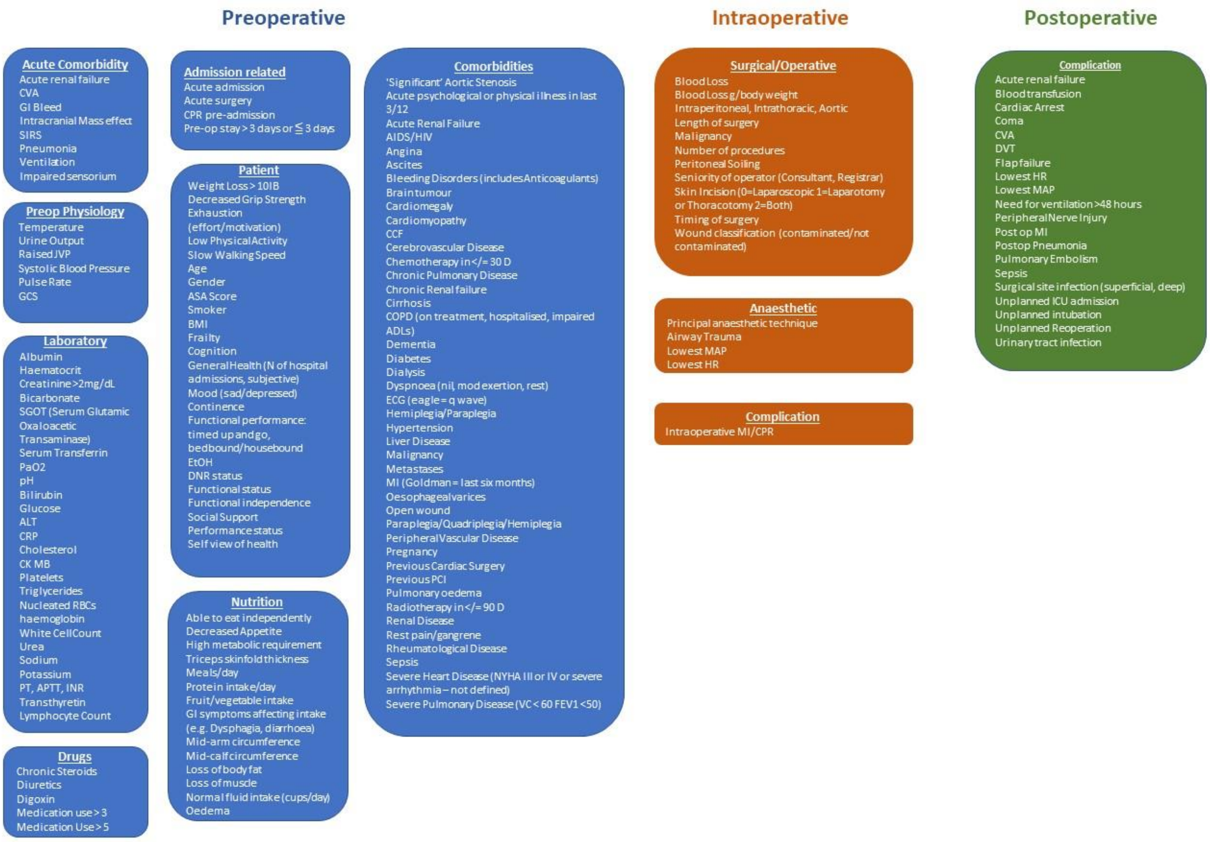

Supplementary Figure 4: Components of validated perioperative scores introduced into Delphi at round two. These variables were identified by deconstructing the risk scores shown in Supplementary Table 1. Groupings are artefactual for clarity and to delineate key temporal aspects of the use of the identified risk scores. Each of these variables were included in Delphi round two, regardless of whether they were present in free-text suggestions from the panel in round one.

review only

Variables suggested by the Delphi panel that did not feature in the development of perioperative risk scores or in a systematic review of structural and process quality indicators.

VARIABLE	DOMAIN	GROUP
**Opioid deprescribing pre-discharge**	Admission	Analgesic Plan
**Opioid dose**	Admission	Analgesic Plan
**Chest injuries**	Admission	Circumstances
**Delayed presentation**	Admission	Circumstances
**Disruption to a patient's routine**	Admission	Circumstances
**Bed Occupancy Rates**	Organisation	Hospital Performance
**Subdural haematoma**	Admission	Circumstances
**Trauma**	Admission	Circumstances
**Indication for surgery (pathology)**	Admission	Details of Surgery
**Orthopaedic v Non-Orthopaedic**	Admission	Details of Surgery
**Delayed Transfer of Care (DToC) Rates**	Organisation	Hospital Performance
**Delayed Transfer of Care**	Admission	Discharge Planning
**ED Admission wait times**	Organisation	Hospital Performance
**Empathy**	Organisation	Staffing
**Enthusiasm and engagement**	Organisation	Staffing
**Health Service Organisation**	Organisation	Health Services Organisation
**Hospital Performance & Strain**	Organisation	Hospital Performance
**Intensive care occupancy**	Organisation	Hospital Performance
**Details of Surgery**	Admission	Intraoperative
**Intraoperative CO2 Control**	Admission	Intraoperative
**Intraoperative Complications**	Admission	Intraoperative
**Medical cover**	Organisation	Staffing
**Intraoperative Physiological Control**	Admission	Intraoperative
**ODP Seniority**	Admission	Intraoperative
**Operator Fatigue**	Admission	Intraoperative

**Scrub nurse Seniority**	Admission	Intraoperative
**Number of outlying patients**	Organisation	Hospital Performance
**Number of patients cared for**	Organisation	Health Services Organisation
**Number of vacant posts**	Organisation	Hospital Performance
**Nursing cover**	Organisation	Staffing
**Occupational therapy cover**	Organisation	Staffing
**Escalation plan made**	Admission	Postoperative
**Organisational culture**	Organisation	Staffing
**Overnight stay in ED**	Organisation	Hospital Performance
**Number of attachments**	Admission	Postoperative
**Pharmacy cover**	Organisation	Staffing
**Physiotherapy cover**	Organisation	Staffing
**Referrals & Reviews**	Admission	Postoperative
**Time to occupational therapist review**	Admission	Postoperative
**Time to pharmacist review (e.g. for medicines reconcilliation)**	Admission	Postoperative
**Anastomotic Breakdown**	Admission	Postoperative Complications
**Practice type**	Organisation	Health Services Organisation
**New diagnosis of chronic conditions (e.g. Dementia)**	Admission	Postoperative Complications
**Poor Wound Healing**	Admission	Postoperative Complications
**Postoperative Dysrhythmia**	Admission	Postoperative Complications
**Postoperative Pain**	Admission	Postoperative Complications
**Postoperative Pneumonia**	Admission	Postoperative Complications
**Postoperative sepsis**	Admission	Postoperative Complications
**DNR status & Escalation plan**	Admission	Preoperative
**How long patients expect to stay in hospital**	Admission	Preoperative
**Primary Care Structure**	Organisation	Health Services Organisation
**Procurement**	Organisation	Policies and Procedures

**Prehabilitation program**	Admission	Preoperative
**Respiratory Virus Rates**	Organisation	Hospital Performance
**Season**	Organisation	Hospital Performance
**Therapy plans**	Admission	Preoperative
**Staffing**	Organisation	Staffing
**Staffing levels**	Organisation	Staffing
**Able to follow commands preoperatively**	Admission	Preoperative Physiology
**Tertiary admission/transfer**	Organisation	Health Services Organisation
**Preoperative CVS impairment**	Admission	Preoperative Physiology
**Preoperative Delirium**	Admission	Preoperative Physiology
**Preoperative Physiology**	Admission	Preoperative Physiology
**Preoperative Renal impairment**	Admission	Preoperative Physiology
**Preoperative Respiratory Failure**	Admission	Preoperative Physiology
**Ward moves/handovers**	Admission	Postoperative

Supplementary Table 2: List of variables that emerged during the Delphi that did not feature in the development of perioperative risk scores or in a systematic review of quality and structural process indicators. For references see text. Blue highlighting demonstrates factors related to the healthcare organisation, Green highlights factors pertaining to the individual admission in question.

For peer review only - http://bmjopen.bmj.com/site/about/guidelines.xhtml

Can process mapping and a multi-site Delphi of perioperative professionals inform our understanding of system-wide factors

Stubbs DJ, Bashford T, Nourallah B, Gilder FJ, Ercole A, Levy N, Clarkson PJ

Supplementary Spreadsheet file

This file contains a complete record of all questions asked of the Delphi participants. Details of what is contair understanding of questions you should also refer to the Supplementary html file which contains an interactive

Column/Variable descriptions
Domain/Question Group
Consensus…
Features in risk scores
Screened for risk scores
In SR of processes
New suggestion
N, median, IQR, min, max, comments

Notes:

For peer review only

ants.

This provides details of where the question featured inside the Delphi questionnaire (for full details of structu
Indicates whether the variable reached the threshold for consensus inclusion or exclusion and, if so, in which
Indicates whether variable featured in commonly used risk scores as identified from ***Moonesinghe et al 2013***
Indicates whether the variable was examined in papers describing the development of the above risk scores.
Indicates whether the variable appears in a systematic review of structure and care process quality indicators
Whether the variable was a new suggestion by the panel and, if so, in which round
Number of responses, median and IQR of responses (on a 5 point likert scale) and free text comments across

Certain variables that the panel voted on were definitions or cut-points of concepts that may or may not have

ure see supplementary html file and main supplementary word document)

round

: https://pubs.asahq.org/anesthesiology/article/119/4/959/11583/Risk-Stratification-Tools-for-Predicting-

All new suggestions were compared against these references

s from **Chazapis et al 2018 https://doi.org/10.1016/j.bja.2017.10.001**. All newly identified variables were ma

both rounds 2 and 3

e featured in earlier rounds or been present in the risk scores. For instance 'BMI' was represented in risk scor

For peer review only

-Morbidity

apped against the suggestions in this paper

es but dichotomised cutpoints of 25/30/35 etc... did not

For peer review only

Name of variable
(Fast) AF
Able to drive
Able to use stairs
Abnormal measurements
Analgesic plan
Access difficulties (e.g. steps)
Accomodation type
Activity
Addison's Disease
Multi modal analgesia
Opioid deprescribing pre-discharge
Opioid dose
Adoption of Sick role
Age
Regional/epidural
Albumin
ALT
Admission circumstances
Admission with Raised ICP
Chest injuries
CPR prior to admission
Delayed presentation
Angina
Ankylosing Spondylitis
Anticoagulants
Disruption to a patient's routine
Antiplatelets
Anxiety
Any diagnosis of CCF
Any diagnosis of PVD
Any form of cancer
Any recent acute illness
Emergency (vs elective) admission
APTT
ASA score
Ascites
AST
Asthma
Availability of services
AVFS (Addenbrookes Vascular Frailty Score)
Bathroom access
Bed Occupancy Rates
Bedbound
Bedroom access
Beliefs & Behaviours
Bilirubin
Bipolar disorder
Blood Product Refusal

For peer review only

Multiple injuries
BMI
BMI25
BMI30
BMI35
BMI40
Body Shape
Bone marrow failure
Bone marrow suppression
Brain tumour
Bronchiectasis
Neck of femur fracture
Sepsis or organ failure on admission
Can the house support single storey living?
Subdural haematoma
Cardiomegaly
Cardiomyopathy
Cardiovascular
Trauma
Care Pathway Documents
CFS > 3
CFS > 4
CFS > 5
BUPA Classification
Characteristics & Demographics
Chemotherapy
By Organ System
Cholesterol
Chronic Dysrhythmia
Chronic open wound
Cirrhosis
CK
Claudication
Coagulopathy
Cognitive Impairment
Community Occupational therapy
Community Physiotherapy
Community/isolation
Comorbidities
Congestive Cardiac Failure
Dutch Surgical Society Score
Continent
COPD
COPD prevents ADLs
Could pay for own care
Indication for surgery (pathology)
CVA
Intraperitoneal, aortic, or intrathoracic operation
Deconditioning
Decreased grip strength

For peer review only

Length of Surgery
Defined by ADLs
Need for stoma formation
Numbers of procedures performed
Orthopaedic v Non-Orthopaedic
Delayed Transfer of Care (DToC) Rates
Peritoneal Soiling
Dental services
Dependency on staff
Depends on cancer site
Depression
Dermatomyositis
Severity of Surgery
Diabetes
Diabetes Control
Diabetes Types
Diagnosis of Dementia
Dialysis
Diet Controlled
Digoxin
Skin incision type (e.g. laparoscopic v laparotomy)
Surgery for Cancer
Distance to hospital
District nursing
Diuretics
Surgical Speciality
DOACs (Direct Oral Anticoagulants)
Drug availability (e.g. anticoagulant reversal agents)
Wound classification (e.g. clean/contaminated)
Delayed Transfer of Care
Discharge Planning
Needing care on discharge
Absence of a specific route of administration (e.g. oral)
Antiemetics & Parkinson's medications
Change in nutritional intake
ECG changes
ED Admission wait times
Edmonton > 10
Electronic Frailty Index
Cytochrome P450 inhibitors/inducers
Empathy
Encephalopathy
Endocrine
Engagement with advice/treatment
Enhanced recovery programs
Enthusiasm and engagement
Episodes of Hypoglycaemia
Drug Bioavailability
Ethnicity
Exercise Tolerance

For peer review only

Exhaustion
Expertise in the care of older people
Facilities
Drug Errors
Family anxieties
Family encouragement
Family/Social Support
FEV1 < 50%
Fitness
Drug Interactions
Fluid intake (cups/day)
Fragile skin
Frailty
Frailty score
Frailty Syndrome: Delirium
Frailty Syndrome: Falls
Frailty Syndrome: Incontinence
Frailty Syndromes
Fruit/veg intake
Function
Gangrene
Drug Management
Genetics
Geography
Drug Metabolism
Glucose
Gout
GP availability
Had [Chemotherapy] within 30 days
Had [Radiotherapy] within 90 days
Haematocrit
Haemoglobin
Has Care Package
Has utilities
Hazards at home
HbA1c
Health Service Organisation
Hearing or visual loss
Hemi/paraplegia
High fat ratio (>25%)
Hip to waist ratio
History of Myocardial Infarction
HIV
Hoarding (of items)
Home Circumstances
Home glucose variability
Home NIPPV
Hospital Performance & Strain
Housebound
Drug Reconcilliation

For peer review only

HTN (Hypertension)
Hypoalbuminemia
Inadvertent administration
Inadvertent omission
Immunosuppressants
Immunosuppressed
Impaired LV function
Lack of an alternative medication
Lack of renal dose adjustment
Methotrexate interactions
Independence
Independent decision maker
Independent with dressing
Independent with eating
Independent with Personal Hygiene
Independent with transfers and walking
Independent, Partially independent, Dependent
Poor absorption
Stress factors
Insulin Dependent
Intensive care occupancy
Timing Errors
Timing of drug reconcilliation (e.g. by pharmacy)
When someone restarts their normal medications
Whether it is performed
Airway complication
Anaesthetic Care
Anaesthetic Seniority
Details of Surgery
Intraoperative
Intraoperative Blood Loss
Ischaemic Changes
Lab Results
Intraoperative CO2 Control
Lack of entertainment
Intraoperative Complications
LBBB
Intraoperative drug errors
Liver disease
Lives alone
Local Authority
Intraoperative Heart rate
Long term effects
Longterm Medications
Loss of body fat
Loss of muscle
Low mood
Lymphocytes
Malignancy
Meals/day

For peer review only

Medical cover
Medication compliance
Mental Health
Mental health services
Metastatic cancer
Intraoperative Hypotension
Midarm circumference
Midcalf circumference
Mild Cognitive Impairment
Mild SOB
Mild/Mod SOB
Moderate SOB
More than 10lb
Intraoperative Myocardial Infarction or CPR
Intraoperative Physiological Control
Night time operating
ODP Seniority
Operator Fatigue
Multiple Sclerosis
Myasthenia Gravis
Out of Hours Operating
n=1 (*N of prescription medications)*
n=2 (N of prescription medications)
n=3
n=4 (N of prescription medications)
n=5
n=7 (N of prescription medications)
Neck diameter
Position on operating list
Preoperative LOS
Principal anaesthetic technique (e.g. GA v RA)
Recovery nurse Seniority
Neurological
Scrub nurse Seniority
Staff Seniority
Non-independent (has decision maker)
Non-independent (no decision maker)
Non-insulin Dependent
NSAIDs
Nucleated RBC
Surgical Seniority
Number of comorbidities
Number of medications
Number of outlying patients
Number of patients cared for
Number of vacant posts
Time-to-surgery
Nursing cover
Nutrition
NYHA III or IV

For peer review only

Obese
Occupational therapy cover
Urgency of surgery
Oedema
On chemotherapy
On Home O2
On Methotrexate
On treatment (for diabetes)
Weekend operating
Delay in necessary review
Escalation plan made
Organisational culture
Inappropriate management
Orthopnea
Osteoarthritis
Osteoporosis
Other
Location and level of care
Overnight stay in ED
Parkinson's Disease
Patient Advocacy
Multidisciplinary working
Patient is a carer
Patient is a vulnerable adult
Patient motivation
Patient's confidence
Patient's view of their own health
Patient's wishes & worries
Performance Status
Peripheral nerve injury
Peripheral oedema
Peripheral Vascular Disease
Number of attachments
Pharmacy cover
Physiotherapy cover
Pituitary Failure
Postoperative care (including analgesia & mobilisation)
Postoperative fluid management
Referrals & Reviews
Platelets
Policies and Procedures
Polymyalgia Rheumatica
Polymyositis
Time to medical review (e.g. for complication)
Poor appetite
Poor Cough
Poor diet
Time to mobilisation
Time to occupational therapist review
Time to pharmacist review (e.g. for medicines reconcilliation)

For peer review only

Anastomotic Breakdown
Blood Transfusion
Cardiac Arrest
Cardiovascular Complications
Constipation
Deep Vein Thrombosis
Delirium
Fall
Flap Failure
Gastrointestinal Complications
GI Bleed
Practice type
Pre-existing delirium
Hypotension
Ileus
Infective Complications
Myocardial Infarction
Need for Postoperative Sedation
New diagnosis of chronic conditions (e.g. Dementia)
Poor Wound Healing
Postoperative Acute Renal Failure
Postoperative Complications
Postoperative Deconditioning
Postoperative Dysrhythmia
Postoperative Pain
Postoperative Pneumonia
Postoperative Pulmonary Complications
Postoperative sepsis
Postoperative ventilation
Pressure Sore
Pulmonary Embolism
Reoperation
Surgical Site Infection
Systemic Inflammatory Response Syndrome (SIRS)
Unplanned ITU Admission
Urinary Tract Infection
An appropriate consent process
Appropriate preoperative ward
DNR status & Escalation plan
How long patients expect to stay in hospital
Multidisciplinary review
Multi-disciplinary working & communication
Patient Expectations
Presence of a telephone advice service
Plan for Drug management
Previous Cardiac Surgery
Previous hospitalisation
Previous ITU admission
Previous PCI
Previous surgery

For peer review only

Primary Care Structure
Planning for care on discharge
PRISMA 7 >/= 3 (*Program of Integration of Services for the maintenanc*
Procedures for minimising conflicts between teams
Procurement
Protein intake
Proteinuria
PT
Planning for the postoperative period
Q waves
Quality of Life
Radiotherapy
Raised creatinine
Raised JVP
Rate of decline
Prehabilitation program
Preoperative Care
Preoperative Communication (patients, teams, families)
Renal
Preoperative Fluid Balance
Requires mobility aids
Respiratory
Respiratory muscle weakness (e.g. Motor Neurone Disease)
Respiratory Virus Rates
Response to chemotherapy
Rest pain
Rest SOB
Rheumatoid Arthritis
Rheumatological
Salt consumption
Schizophrenia
Scoliosis
Preoperative Fluid Management
Season
Self-reported malnourishment
Preoperative IV Fluid Use
Seronegative spondyloarthropathy
Severe Aortic Stenosis
Preoperative Medical Care
Sex
Preoperative Planning
Shortness of breath
Sjogrens syndrome
Shared decision making process
Smoking history
Social care
Socioeconomics
Starvation (NBM) time
Specific service availability
Splenomegaly

For peer review only

Therapy plans
Staff traits, skills, behaviours
Staffing
Staffing levels
Stairs/more than one storey with no lift
Starting weight
Understand their role in recovery
Strenuous SOB
Understanding of family expectations
Use of a Risk Tool
Substance misuse (incl. alcohol)
Supplement requirement
Surgery cancellation rates
Use of the WHO Checklist
Able to follow commands preoperatively
Potassium
Preoperative acid base status (e.g. pH, bicarbonate)
Symptoms that limit eating
Preoperative Acute Renal impairment
Systemic Lupus Erythematosus
Systemic Sclerosis
T wave inversion
Taking GLP1 analogue
Taking metformin
Taking SGLT2
Tertiary admission/transfer
Their General Health
Their GP Practice type (e.g. practice type)
Preoperative Creatinine
Thrombocytopenia
Preoperative CVS impairment
Preoperative Delirium
Preoperative Electrolytes
Preoperative GCS
Timed get up and go
Timeframe (of weight loss)
Preoperative Hypotension
Preoperative inotrope requirement
Preoperative Neurological impairment
Training levels
Transferrin
Transthyretin
Preoperative O2 Saturations
Triceps skinfold thickness
Triglycerides
Type I v Type II
Type of accomodation (e.g. flat)
Ulceration
Preoperative PaCO2
Preoperative PaO2

For peer review only

Understanding of their condition
Understanding of their situation
Unintentional *(weight loss)*
Preoperative Physiology
Preoperative pneumonia
Preoperative Renal impairment
Preoperative Respiratory Failure
Preoperative Temperature
Varices
Vasculitis
VC < 60%
Visited in hospital
Ward moves/handovers
Warfarin
WCC
Preoperative Urea
Weight
Weight loss
Preoperative Urine output
Preoperative ventilation
Sodium

For peer review only

Domain	Question Group	Consensus (any round)?
Patient	Comorbidities	1
Patient	Function	0
Patient	Function	1
Patient	Comorbidities	1
Admission	Analgesic Plan	1
Patient	Home Circumstances	1
Patient	Home Circumstances	1
Patient	Function	1
Patient	Comorbidities	1
Admission	Analgesic Plan	1
Admission	Analgesic Plan	1
Admission	Analgesic Plan	1
Patient	Beliefs & Behaviours	1
Patient	Characteristics	1
Admission	Analgesic Plan	1
Patient	Lab Results	1
Patient	Lab Results	1
Admission	Circumstances	1
Admission	Circumstances	1
Admission	Circumstances	1
Admission	Circumstances	1
Admission	Circumstances	1
Patient	Comorbidities	1
Patient	Comorbidities	0
Patient	Longterm medications	1
Admission	Circumstances	0
Patient	Longterm medications	1
Patient	Comorbidities	1
Patient	Comorbidities	1
Patient	Comorbidities	1
Patient	Comorbidities	0
Patient	Comorbidities	1
Admission	Circumstances	1
Patient	Lab Results	1
Patient	Characteristics	1
Patient	Comorbidities	1
Patient	Lab Results	1
Patient	Comorbidities	1
Organisation	Health Services Organisation	1
Patient	Frailty	0
Patient	Home Circumstances	1
Organisation	Hospital Performance	1
Patient	Home Circumstances	1
Patient	Home Circumstances	1
Patient	Beliefs & Behaviours	0
Patient	Lab Results	1
Patient	Comorbidities	1
Patient	Beliefs & Behaviours	1

For peer review only

Admission	Circumstances	1
Patient	Comorbidities	1
Patient	Comorbidities	0
Patient	Comorbidities	0
Patient	Comorbidities	0
Patient	Comorbidities	1
Patient	Comorbidities	0
Patient	Comorbidities	1
Patient	Comorbidities	1
Patient	Comorbidities	1
Patient	Comorbidities	1
Admission	Circumstances	1
Admission	Circumstances	1
Patient	Home Circumstances	1
Admission	Circumstances	1
Patient	Comorbidities	1
Patient	Comorbidities	1
Patient	Comorbidities	1
Admission	Circumstances	1
Organisation	Policies and Procedures	1
Patient	Frailty	1
Patient	Frailty	1
Patient	Frailty	1
Admission	Details of Surgery	0
Patient	Characteristics	0
Patient	Comorbidities	1
Admission	Details of Surgery	1
Patient	Lab Results	0
Patient	Comorbidities	1
Patient	Comorbidities	1
Patient	Lab Results	1
Patient	Lab Results	0
Patient	Comorbidities	1
Patient	Comorbidities	1
Patient	Comorbidities	1
Patient	Home Circumstances	1
Patient	Home Circumstances	1
Patient	Home Circumstances	1
Patient	Comorbidities	1
Patient	Comorbidities	1
Admission	Details of Surgery	0
Patient	Function	1
Patient	Comorbidities	1
Patient	Comorbidities	1
Patient	Home Circumstances	0
Admission	Details of Surgery	1
Patient	Comorbidities	1
Admission	Details of Surgery	1
Patient	Frailty	1
Patient	Frailty	1

For peer review only

Admission	Details of Surgery	1
Patient	Function	1
Admission	Details of Surgery	1
Admission	Details of Surgery	1
Admission	Details of Surgery	0
Organisation	Hospital Performance	1
Admission	Details of Surgery	1
Patient	Home Circumstances	0
Patient	Beliefs & Behaviours	1
Patient	Comorbidities	0
Patient	Comorbidities	1
Patient	Comorbidities	0
Admission	Details of Surgery	1
Patient	Comorbidities	1
Patient	Comorbidities	1
Patient	Comorbidities	1
Patient	Comorbidities	1
Patient	Comorbidities	1
Patient	Comorbidities	1
Patient	Comorbidities	1
Admission	Details of Surgery	1
Admission	Details of Surgery	1
Patient	Home Circumstances	1
Patient	Home Circumstances	1
Patient	Longterm medications	1
Admission	Details of Surgery	1
Patient	Longterm medications	1
Organisation	Policies and Procedures	1
Admission	Details of Surgery	1
Admission	Discharge Planning	1
Admission	Discharge Planning	1
Admission	Discharge Planning	1
Admission	Drug Management	1
Admission	Drug Management	1
Admission	Drug Management	1
Patient	Comorbidities	1
Organisation	Hospital Performance	1
Patient	Frailty	0
Patient	Frailty	0
Admission	Drug Management	1
Organisation	Staffing	1
Patient	Comorbidities	1
Patient	Comorbidities	1
Patient	Beliefs & Behaviours	1
Organisation	Policies and Procedures	1
Organisation	Staffing	1
Patient	Comorbidities	1
Admission	Drug Management	1
Patient	Characteristics	1
Patient	Comorbidities	1

For peer review only

Patient	Frailty	1
Organisation	Staffing	1
Patient	Home Circumstances	1
Admission	Drug Management	1
Patient	Home Circumstances	1
Patient	Home Circumstances	1
Patient	Home Circumstances	1
Patient	Comorbidities	1
Patient	Function	1
Admission	Drug Management	1
Patient	Comorbidities	1
Patient	Comorbidities	1
Patient	Frailty	1
Patient	Frailty	1
Patient	Frailty	1
Patient	Frailty	1
Patient	Frailty	0
Patient	Frailty	1
Patient	Comorbidities	1
Patient	Function	1
Patient	Comorbidities	1
Admission	Drug Management	1
Patient	Characteristics	1
Patient	Home Circumstances	1
Admission	Drug Management	1
Patient	Lab Results	1
Patient	Comorbidities	0
Patient	Home Circumstances	1
Patient	Comorbidities	1
Patient	Comorbidities	1
Patient	Lab Results	1
Patient	Lab Results	1
Patient	Function	1
Patient	Home Circumstances	1
Patient	Home Circumstances	1
Patient	Comorbidities	1
Organisation	Health Services Organisation	1
Patient	Characteristics	1
Patient	Comorbidities	1
Patient	Comorbidities	0
Patient	Comorbidities	0
Patient	Comorbidities	1
Patient	Comorbidities	1
Patient	Home Circumstances	1
Patient	Home Circumstances	1
Patient	Comorbidities	1
Patient	Comorbidities	1
Organisation	Hospital Performance	0
Patient	Function	1
Admission	Drug Management	1

For peer review only

Patient	Comorbidities	1
Patient	Comorbidities	1
Admission	Drug Management	1
Admission	Drug Management	1
Patient	Longterm medications	1
Patient	Comorbidities	1
Patient	Comorbidities	1
Admission	Drug Management	1
Admission	Drug Management	1
Admission	Drug Management	1
Patient	Function	1
Patient	Home Circumstances	1
Patient	Function	1
Patient	Function	1
Patient	Function	1
Patient	Function	1
Patient	Function	1
Admission	Drug Management	1
Admission	Drug Management	1
Patient	Comorbidities	1
Organisation	Hospital Performance	1
Admission	Drug Management	1
Admission	Drug Management	1
Admission	Drug Management	1
Admission	Drug Management	1
Admission	Intraoperative	1
Admission	Intraoperative	1
Admission	Intraoperative	1
Admission	Intraoperative	1
Admission	Intraoperative	1
Admission	Intraoperative	1
Patient	Comorbidities	1
Patient	Lab Results	1
Admission	Intraoperative	1
Patient	Home Circumstances	1
Admission	Intraoperative	1
Patient	Comorbidities	1
Admission	Intraoperative	1
Patient	Comorbidities	1
Patient	Home Circumstances	1
Patient	Home Circumstances	0
Admission	Intraoperative	1
Patient	Comorbidities	1
Patient	Longterm medications	1
Patient	Comorbidities	1
Patient	Frailty	1
Patient	Comorbidities	1
Patient	Lab Results	1
Patient	Comorbidities	1
Patient	Comorbidities	1

For peer review only

Organisation	Staffing	1
Patient	Beliefs & Behaviours	1
Patient	Comorbidities	1
Patient	Home Circumstances	1
Patient	Comorbidities	1
Admission	Intraoperative	1
Patient	Comorbidities	0
Patient	Comorbidities	0
Patient	Comorbidities	1
Patient	Comorbidities	1
Patient	Comorbidities	1
Patient	Comorbidities	1
Patient	Comorbidities	1
Admission	Intraoperative	1
Admission	Intraoperative	1
Admission	Intraoperative	1
Admission	Intraoperative	0
Admission	Intraoperative	1
Patient	Comorbidities	1
Patient	Comorbidities	1
Admission	Intraoperative	1
Patient	Longterm medications	0
Patient	Longterm medications	0
Patient	Longterm medications	0
Patient	Longterm medications	1
Patient	Longterm medications	1
Patient	Longterm medications	1
Patient	Comorbidities	0
Admission	Intraoperative	1
Admission	Intraoperative	1
Admission	Intraoperative	1
Admission	Intraoperative	1
Patient	Comorbidities	1
Admission	Intraoperative	1
Admission	Intraoperative	1
Patient	Home Circumstances	1
Patient	Home Circumstances	1
Patient	Comorbidities	1
Patient	Longterm medications	1
Patient	Lab Results	0
Admission	Intraoperative	1
Patient	Comorbidities	1
Patient	Longterm medications	1
Organisation	Hospital Performance	1
Organisation	Health Services Organisation	1
Organisation	Hospital Performance	1
Admission	Intraoperative	1
Organisation	Staffing	1
Patient	Comorbidities	1
Patient	Comorbidities	1

For peer review only

Patient	Comorbidities	1
Organisation	Staffing	1
Admission	Intraoperative	1
Patient	Comorbidities	1
Patient	Comorbidities	1
Patient	Comorbidities	1
Patient	Comorbidities	1
Patient	Comorbidities	1
Admission	Intraoperative	1
Admission	Postoperative	1
Admission	Postoperative	1
Organisation	Staffing	1
Admission	Postoperative	1
Patient	Comorbidities	1
Patient	Comorbidities	0
Patient	Comorbidities	0
Patient	Comorbidities	1
Admission	Postoperative	1
Organisation	Hospital Performance	1
Patient	Comorbidities	1
Patient	Home Circumstances	1
Admission	Postoperative	1
Patient	Home Circumstances	1
Patient	Home Circumstances	1
Patient	Beliefs & Behaviours	1
Patient	Beliefs & Behaviours	1
Patient	Beliefs & Behaviours	1
Patient	Beliefs & Behaviours	1
Patient	Function	1
Patient	Comorbidities	1
Patient	Comorbidities	1
Patient	Comorbidities	1
Admission	Postoperative	1
Organisation	Staffing	1
Organisation	Staffing	1
Patient	Comorbidities	1
Admission	Postoperative	1
Admission	Postoperative	1
Admission	Postoperative	1
Patient	Lab Results	1
Organisation	Policies and Procedures	1
Patient	Comorbidities	0
Patient	Comorbidities	1
Admission	Postoperative	1
Patient	Comorbidities	0
Patient	Comorbidities	1
Patient	Comorbidities	1
Admission	Postoperative	1
Admission	Postoperative	1
Admission	Postoperative	1

For peer review only

Admission	Postoperative Complications	1
Admission	Postoperative Complications	1
Admission	Postoperative Complications	1
Admission	Postoperative Complications	1
Admission	Postoperative Complications	1
Admission	Postoperative Complications	1
Admission	Postoperative Complications	1
Admission	Postoperative Complications	1
Admission	Postoperative Complications	1
Admission	Postoperative Complications	1
Admission	Postoperative Complications	1
Organisation	Health Services Organisation	1
Patient	Comorbidities	1
Admission	Postoperative Complications	1
Admission	Postoperative Complications	1
Admission	Postoperative Complications	1
Admission	Postoperative Complications	1
Admission	Postoperative Complications	0
Admission	Postoperative Complications	1
Admission	Postoperative Complications	1
Admission	Postoperative Complications	1
Admission	Postoperative Complications	1
Admission	Postoperative Complications	1
Admission	Postoperative Complications	1
Admission	Postoperative Complications	1
Admission	Postoperative Complications	1
Admission	Postoperative Complications	1
Admission	Postoperative Complications	1
Admission	Postoperative Complications	1
Admission	Postoperative Complications	1
Admission	Postoperative Complications	1
Admission	Postoperative Complications	1
Admission	Postoperative Complications	1
Admission	Postoperative Complications	1
Admission	Postoperative Complications	1
Admission	Postoperative Complications	1
Admission	Preoperative	1
Admission	Preoperative	1
Admission	Preoperative	1
Admission	Preoperative	1
Admission	Preoperative	1
Admission	Preoperative	1
Admission	Preoperative	1
Organisation	Health Services Organisation	1
Admission	Preoperative	1
Patient	Comorbidities	1
Patient	Comorbidities	1
Patient	Comorbidities	1
Patient	Comorbidities	1
Patient	Comorbidities	0

Organisation	Health Services Organisation	1
Admission	Preoperative	1
Patient	Frailty	0
Organisation	Policies and Procedures	1
Organisation	Policies and Procedures	1
Patient	Comorbidities	1
Patient	Comorbidities	1
Patient	Lab Results	1
Admission	Preoperative	1
Patient	Comorbidities	1
Patient	Characteristics	1
Patient	Comorbidities	1
Patient	Comorbidities	1
Patient	Comorbidities	1
Patient	Frailty	1
Admission	Preoperative	1
Admission	Preoperative	1
Admission	Preoperative	1
Patient	Comorbidities	1
Admission	Preoperative	1
Patient	Function	1
Patient	Comorbidities	1
Patient	Comorbidities	1
Organisation	Hospital Performance	1
Patient	Comorbidities	0
Patient	Comorbidities	1
Patient	Comorbidities	1
Patient	Comorbidities	1
Patient	Comorbidities	1
Patient	Comorbidities	1
Patient	Comorbidities	1
Patient	Comorbidities	1
Admission	Preoperative	1
Organisation	Hospital Performance	1
Patient	Comorbidities	1
Admission	Preoperative	1
Patient	Comorbidities	1
Patient	Comorbidities	1
Admission	Preoperative	1
Patient	Characteristics	0
Admission	Preoperative	1
Patient	Comorbidities	1
Patient	Comorbidities	1
Admission	Preoperative	1
Patient	Characteristics	1
Patient	Home Circumstances	1
Patient	Home Circumstances	1
Admission	Preoperative	1
Patient	Home Circumstances	1
Patient	Comorbidities	1

For peer review only

Admission	Preoperative	1
Organisation	Staffing	1
Organisation	Staffing	1
Organisation	Staffing	1
Patient	Home Circumstances	1
Patient	Comorbidities	1
Admission	Preoperative	1
Patient	Comorbidities	1
Admission	Preoperative	1
Admission	Preoperative	1
Patient	Comorbidities	1
Patient	Comorbidities	1
Organisation	Hospital Performance	1
Admission	Preoperative	1
Admission	Preoperative Physiology	1
Admission	Preoperative Physiology	1
Admission	Preoperative Physiology	1
Patient	Comorbidities	1
Admission	Preoperative Physiology	1
Patient	Comorbidities	1
Patient	Comorbidities	1
Patient	Comorbidities	1
Patient	Comorbidities	0
Patient	Comorbidities	1
Patient	Comorbidities	1
Organisation	Health Services Organisation	1
Patient	Characteristics	1
Patient	Home Circumstances	1
Admission	Preoperative Physiology	1
Patient	Lab Results	1
Admission	Preoperative Physiology	1
Admission	Preoperative Physiology	1
Admission	Preoperative Physiology	1
Admission	Preoperative Physiology	1
Patient	Frailty	0
Patient	Comorbidities	1
Admission	Preoperative Physiology	1
Admission	Preoperative Physiology	1
Admission	Preoperative Physiology	1
Organisation	Staffing	1
Patient	Lab Results	0
Patient	Lab Results	0
Admission	Preoperative Physiology	1
Patient	Comorbidities	0
Patient	Lab Results	0
Patient	Comorbidities	1
Patient	Home Circumstances	1
Patient	Comorbidities	1
Admission	Preoperative Physiology	1
Admission	Preoperative Physiology	1

For peer review only

Patient	Beliefs & Behaviours	1
Patient	Beliefs & Behaviours	1
Patient	Comorbidities	1
Admission	Preoperative Physiology	1
Admission	Preoperative Physiology	1
Admission	Preoperative Physiology	1
Admission	Preoperative Physiology	1
Admission	Preoperative Physiology	1
Patient	Comorbidities	1
Patient	Comorbidities	1
Patient	Comorbidities	1
Patient	Home Circumstances	1
Admission	Postoperative	1
Patient	Longterm medications	1
Patient	Lab Results	1
Admission	Preoperative Physiology	1
Patient	Comorbidities	1
Patient	Comorbidities	1
Admission	Preoperative Physiology	1
Admission	Preoperative Physiology	1
Admission	Preoperative Physiology	1

For peer review only

Consensus Include (Round 2)	Consensus Include (Round 3)
0	1
0	0
0	1
1	0
1	0
1	0
1	0
1	0
0	1
1	0
1	0
1	0
1	0
1	0
1	0
1	0
1	0
1	0
1	0
1	0
1	0
1	0
1	0
0	0
1	0
0	1
0	1
1	0
1	0
1	0
0	0
1	0
1	0
0	1
1	0
1	0
1	0
1	0
1	0
0	0
0	1
1	0
1	0
0	1
0	0
1	0
1	0
0	1

1	0
1	0
0	0
0	0
0	0
0	1
0	0
0	1
0	1
1	0
1	0
0	1
0	1
0	1
0	1
1	0
1	0
1	0
1	0
1	0
0	1
0	1
0	1
0	0
0	0
1	0
1	0
0	0
1	0
1	0
1	0
0	0
0	1
1	0
1	0
0	1
0	1
0	1
1	0
1	0
0	0
1	0
1	0
1	0
0	0
1	0
1	0
1	0
0	1
1	0

For peer review only

1	0
1	0
1	0
1	0
0	0
1	0
1	0
0	0
1	0
0	0
1	0
0	1
1	0
1	0
1	0
1	0
1	0
1	0
0	1
1	0
1	0
1	0
0	1
0	1
1	0
0	1
1	0
1	0
1	0
1	0
1	0
1	0
1	0
1	0
0	1
0	1
0	1
0	0
0	0
0	1
1	0
0	1
1	0
1	0
1	0
0	1
0	1
1	0
0	1
0	1

For peer review only

1	0
1	0
1	0
1	0
1	0
1	0
1	0
1	0
1	0
1	0
1	0
1	0
1	0
1	0
0	1
0	1
0	0
1	0
1	0
1	0
1	0
1	0
1	0
1	0
1	0
1	0
0	0
0	1
1	0
0	1
1	0
1	0
1	0
1	0
1	0
1	0
1	0
1	0
1	0
0	1
0	0
1	0
0	1
0	1
1	0
0	1
0	1
0	0
1	0
1	0

For peer review only

1	0
0	1
1	0
1	0
0	1
1	0
1	0
1	0
1	0
0	1
1	0
1	0
1	0
1	0
1	0
1	0
0	1
1	0
0	1
1	0
0	1
0	1
1	0
1	0
1	0
1	0
1	0
1	0
1	0
1	0
1	0
0	1
1	0
1	0
0	1
1	0
0	1
1	0
1	0
1	0
0	0
1	0
0	1
1	0
1	0
0	1
1	0
1	0
1	0
1	0

For peer review only

1	0
1	0
1	0
0	1
1	0
1	0
0	0
0	0
1	0
1	0
1	0
1	0
1	0
1	0
1	0
1	0
0	0
1	0
1	0
1	0
1	0
0	0
0	0
0	0
0	1
0	1
0	1
0	0
0	1
1	0
1	0
0	1
1	0
0	1
1	0
1	0
0	1
1	0
1	0
0	0
1	0
1	0
1	0
0	1
1	0
1	0
1	0
1	0
1	0
1	0

For peer review only

1	0
1	0
1	0
1	0
1	0
0	1
0	1
1	0
0	1
1	0
1	0
0	1
1	0
0	1
0	0
0	0
1	0
1	0
0	1
1	0
1	0
1	0
1	0
1	0
1	0
1	0
1	0
1	0
1	0
1	0
1	0
1	0
1	0
1	0
1	0
0	1
1	0
1	0
1	0
1	0
1	0
0	0
0	1
1	0
0	0
0	1
1	0
1	0
1	0
1	0

For peer review only

For peer review only

1	0
1	0
1	0
1	0
1	0
1	0
1	0
1	0
1	0
1	0
1	0
1	0
1	0
1	0
1	0
1	0
1	0
0	0
0	1
1	0
1	0
1	0
1	0
0	1
1	0
1	0
1	0
1	0
1	0
1	0
1	0
1	0
1	0
1	0
1	0
1	0
1	0
1	0
1	0
1	0
1	0
1	0
1	0
0	1
1	0
1	0
1	0
0	1
0	1
0	0

1	0
1	0
0	0
1	0
0	1
1	0
0	1
1	0
1	0
1	0
1	0
1	0
1	0
1	0
1	0
0	1
1	0
1	0
1	0
1	0
1	0
1	0
0	1
1	0
0	0
1	0
1	0
0	1
1	0
1	0
1	0
0	1
1	0
0	1
1	0
0	1
0	1
1	0
1	0
0	0
1	0
1	0
0	1
1	0
1	0
0	1
1	0
1	0
1	0
0	1

For peer review only

1	0
1	0
1	0
1	0
0	1
0	1
0	1
0	0
1	0
1	0
1	0
0	1
0	1
1	0
1	0
1	0
1	0
1	0
1	0
0	1
0	1
0	1
0	0
0	1
0	1
0	1
1	0
1	0
1	0
0	1
1	0
1	0
1	0
1	0
0	0
0	1
1	0
1	0
1	0
1	0
0	0
0	0
1	0
0	0
0	0
0	1
1	0
0	1
1	0
1	0

For peer review only

1	0
0	1
0	1
1	0
1	0
1	0
1	0
1	0
1	0
0	1
1	0
1	0
1	0
1	0
1	0
1	0
1	0
1	0
1	0
1	0
1	0

For peer review only

Consensus Exclude (Round 2)	Consensus Exclude (Round 3)	Feature in Risk Scores?
0	0	0
0	0	0
0	0	0
0	0	0
0	0	0
0	0	0
0	0	0
0	0	0
0	0	0
0	0	0
0	0	0
0	0	0
0	0	0
0	0	1
0	0	0
0	0	1
0	0	1
0	0	1
0	0	1
0	0	0
0	0	1
0	0	0
0	0	1
0	0	0
0	0	0
0	0	0
0	0	0
0	0	0
0	0	1
0	0	1
0	0	0
0	0	1
0	0	1
0	0	1
0	0	1
0	0	1
0	0	1
0	0	1
0	0	0
0	0	0
0	0	0
0	0	0
0	0	0
0	0	0
0	0	0
0	0	1
0	0	0
0	0	0

For peer review only

0	0	0
0	0	1
0	0	0
0	0	0
0	0	0
0	0	0
0	0	0
0	0	0
0	0	0
0	0	1
0	0	0
0	0	0
0	0	0
0	0	0
0	0	0
0	0	1
0	0	1
0	0	1
0	0	0
0	0	0
0	0	0
0	0	0
0	0	0
0	0	1
0	0	0
0	0	1
0	0	0
0	0	1
0	0	1
0	0	1
0	0	1
0	0	1
0	0	0
0	0	1
0	0	1
0	0	0
0	0	0
0	0	0
0	0	0
0	0	1
0	0	1
0	0	0
0	0	1
0	0	0
0	0	0
0	0	0
0	0	1
0	0	1
0	0	0
0	0	1

For peer review only

0	0	1
0	0	1
0	0	0
0	0	1
0	0	0
0	0	0
0	0	1
0	0	0
		0
0	0	0
0	0	0
0	0	0
0	0	0
0	0	1
0	0	0
0	0	0
0	0	1
0	0	1
0	0	0
0	0	1
0	0	1
0	0	1
0	0	0
0	0	0
0	0	1
0	0	0
0	0	0
0	0	0
0	0	1
0	0	0
0	0	0
0	0	0
0	0	0
0	0	0
0	0	0
0	0	1
0	0	0
0	0	0
0	0	0
0	0	0
0	0	0
0	0	0
0	0	0
0	0	0
0	0	0
0	0	0
0	0	0
0	0	0
0	0	0
0	0	0

For peer review only

0	0	1
0	0	0
0	0	0
0	0	0
0	0	0
0	0	0
0	0	1
0	0	1
0	0	0
0	0	0
0	0	1
0	0	0
0	0	1
0	0	0
0	0	0
0	0	0
0	0	0
0	0	0
0	0	1
0	0	0
0	0	1
0	0	0
0	0	0
0	0	0
0	0	0
0	0	1
0	0	0
0	0	0
0	0	1
0	0	1
0	0	1
0	0	1
0	0	0
0	0	0
0	0	0
0	0	0
0	0	0
0	0	0
0	0	1
0	0	0
0	0	0
0	0	1
0	0	1
0	0	0
0	0	0
0	0	0
0	0	0
0	0	0
0	0	0
0	0	0

For peer review only

0	0	1
0	0	1
0	0	0
0	0	0
0	0	0
0	0	0
0	0	1
0	0	0
0	0	0
0	0	0
0	0	0
0	0	0
0	0	0
0	0	0
0	0	0
0	0	0
0	0	0
0	0	0
0	0	0
0	0	0
0	0	0
0	0	0
0	0	0
0	0	0
0	0	0
0	0	1
0	0	0
0	0	0
0	0	0
0	0	0
0	0	1
0	0	0
0	0	1
0	0	0
0	0	0
0	0	0
0	0	0
0	0	0
0	0	1
0	0	0
0	0	0
0	0	1
0	0	0
0	0	1
0	0	0
0	0	1
0	0	1
0	0	1
0	0	1
0	0	1

For peer review only

0	0	0
		0
0	0	0
0	0	0
0	0	1
0	0	0
0	0	1
0	0	1
0	0	1
0	0	0
0	0	0
0	0	0
0	0	1
0	0	1
0	0	0
0	0	0
0	0	0
0	0	0
0	0	0
0	0	0
0	0	0
0	0	0
0	0	0
0	0	1
0	0	0
0	0	1
0	0	0
0	0	0
0	0	0
0	0	1
0	0	1
0	0	0
0	0	0
0	0	0
0	0	0
0	0	0
0	0	0
0	0	0
0	0	0
0	0	1
0	0	1
0	0	0
0	0	1
0	0	0
0	0	0
0	0	0
0	0	1
0	0	0
0	0	1
0	0	1

For peer review only

0	0	0
0	0	0
0	0	1
0	0	0
0	0	1
0	0	0
0	0	0
0	0	0
0	0	0
0	0	0
0	0	0
0	0	0
0	0	0
0	0	0
0	0	0
0	0	0
0	0	0
0	0	0
0	0	0
0	0	0
0	0	0
0	0	0
0	0	0
0	0	0
		0
		0
0	0	0
0	0	0
0	0	0
0	0	1
0	0	1
0	0	1
0	0	0
0	0	0
0	0	0
0	0	0
0	0	0
0	0	0
0	0	0
0	0	1
0	0	0
0	0	0
0	0	0
0	0	0
0	0	1
0	0	0
0	0	1
0	0	0
0	0	0
0	0	0

For peer review only

0	0	0
0	0	1
0	0	1
0	0	1
0	0	0
0	0	1
0	0	1
0	0	1
0	0	1
0	0	0
0	0	1
0	0	0
0	0	0
0	0	1
0	0	0
0	0	0
0	0	1
0	0	0
0	0	0
0	0	0
0	0	1
0	0	0
0	0	1
0	0	0
0	0	0
0	0	0
0	0	1
0	0	0
0	0	1
0	0	0
0	0	1
0	0	1
0	0	0
0	0	1
0	0	1
0	0	1
0	0	0
0	0	0
0	0	0
0	0	0
0	0	0
0	0	0
0	0	0
0	0	0
0	0	0
0	0	1
0	0	1
0	0	0
0	0	1
0	0	0

For peer review only

0	0	0
0	0	0
0	0	0
0	0	0
0	0	0
0	0	1
0	0	0
0	0	1
0	0	0
0	0	1
0	0	0
0	0	1
0	0	1
0	0	1
0	0	1
0	0	0
0	0	0
0	0	0
0	0	1
0	0	0
0	0	0
0	0	1
0	0	0
0	0	0
0	0	0
0	0	1
0	0	0
0	0	0
0	0	1
0	0	1
0	0	0
0	0	0
0	0	0
0	0	0
0	0	1
0	0	0
0	0	0
0	0	1
0	0	1
0	0	1
0	0	0
0	0	0
0	0	0
0	0	0
0	0	1
0	0	0
0	0	0
0	0	0
0	0	0
0	0	0

For peer review only

0	0	0
0	0	0
0	0	0
0	0	0
0	0	0
0	0	1
0	0	0
0	1	0
0	0	0
0	0	0
0	0	0
0	0	0
0	0	0
0	0	0
0	0	0
0	0	1
0	0	1
0	0	1
0	0	1
0	0	0
0	0	0
0	0	0
0	0	0
0	0	0
0	0	0
0	0	0
0	0	0
0	0	0
0	0	1
0	0	0
0	0	0
0	0	0
0	0	0
0	0	1
0	0	0
0	0	0
0	0	1
0	0	1
0	0	0
0	0	0
0	0	1
0	0	1
0	0	0
0	0	1
0	0	1
0	0	0
0	0	0
0	0	0
0	0	0
0	0	1

For peer review only

0	0	0
0	0	0
0	0	0
0	0	0
0	0	1
0	0	0
0	0	0
0	0	1
0	0	1
0	0	0
0	0	1
0	0	0
0	0	0
0	0	1
0	0	1
0	0	1
0	0	0
0	0	1
0	0	1
0	0	1
0	0	1

For peer review only

Screened for Risk Scores?	In SR of processes	New sugge	New sugge	N(round2)	Median(ro	IQR(round2
	0	0	1			
	0	0	1			
1	0	0	1			
	0	0	0	28	4	1
	1	1	0	26	4	1
	0	1	0	26	4	0
	0	1	0	28	4	1
	0	1	0	27	5	1
	0	0	1			
	1	1	0	24	5	1
	0	1	0	25	4	1
	0	1	0	25	4	1
	0	1	0	32	4.5	1
1		0	0	31	5	1
1	1	1	0	24	4	1
1		0	0	25	4	0
		0	0	21	4	1
	0	0	0	27	4	1
	0	0	0	25	5	1
	0	1	0	24	5	1
	0	0	0	27	5	0
	0	1	0	27	5	1
1		0	0	29	5	1
	0	0	1			
	0	1	0	26	5	1
	0	1	0	27	3	1
	0	0	1			
	0	1	0	27	4	0
		0	0	26	4	1
		0	0	24	4	1
1	0	0	1			
1		0	0	29	5	1
1	1	0	0	27	5	1
		0	0	20	4	1.25
1		0	0	25	4	1
		0	0	27	5	1
1		0	0	18	4	1
		0	0	28	4	1
	1	1	0	27	5	1
	0	0	1			
	0	0	1			
	0	1	0	27	5	1
1	0	1	0	27	5	0
	0	0	1			
	0	1	0	33	4	2
		0	0	21	4	1
	0	1	0	26	4	1
	0	1	0	31	4	2

For peer review only

1	0	1	0	24	5	0
1		0	0	28	4	1
	0	0	1			
	0	0	1			
	0	0	1			
	0	0	1			
	0	0	1			
	0	0	1			
	0	0	1			
1		0	0	26	4	1
	0	1	0	27	5	1
	1	0	1			
1	0	0	1			
	0	1	0			
	0	0	1			
1		0	0	27	4	1
		0	0	28	5	1
		0	0	30	5	0
	0	0	0	25	5	1
	1	1	0	25	4	1
	0	1	0			
	0	1	0			
	0	1	0			
1	0	1	0	7	3	1
	0	0	0	31	4	1.5
		0	0	27	4	1
	1	1	0	20	4	0.5
		0	0	23	3	0
1		0	0	28	4	1
1		0	0	28	4	1
1		0	0	27	5	1
		0	0	22	3	1
	0	0	1			
		0	0	26	5	0.75
		0	0	28	5	1
	0	0	1			
	0	0	1			
	0	0	1			
		0	0	30	5	0
1		0	0	28	5	0.25
1	0	0	0	3	3	1
1	0	1	0	27	4	1
		0	0	28	5	1
	0	0	0	27	5	0.5
	0	1	0	27	3	1
	0	1	0	25	5	1
1		0	0	29	5	1
1	0	1	0	16	4	1
	0	1	0	25	4	2
		0	0	26	4	0.75

For peer review only

1	1	0	0	24	5	1
1		0	0	28	4	1
	1	1	0	22	4	0.75
1	0	0	0	24	4	1
	0	1	0	21	3	1
	0	1	0	25	4	1
1	0	0	0	21	5	1
	0	0	1			
	0	1	0	32	4	1
	0	0	1			
1	0	1	0	27	4	0
	0	0	1			
	1	1	0	24	5	0
1	1	0	0	29	5	1
	1	1	0	29	5	1
	1	1	0	28	4	1
1		0	0	27	4	1
1		0	0	27	5	0
	0	1	0	26	3	1
		0	0	24	4	1
1	0	0	0	18	4	1
	1	0	0	22	4.5	1
	0	1	0	24	3	1
	0	0	1			
		0	0	24	4	1
1	1	0	1	24	4	2
	0	1	0	25	5	1
	1	1	0	24	4	1
1	0	0	0	24	5	1
	0	1	0	24	4	1
	1	1	0	25	5	1
	1	1	0	25	4	1
	1	1	0	20	4	0.25
	1	1	0	19	4	1
	1	1	0			
1		0	0	29	4	2
	0	0	1			
	0	0	1			
	0	0	1			
	1	0	1			
	0	1	0	27	5	1
1	0	0	1			
	0	1	0	30	5	1
	0	1	0	31	5	1
	1	1	0	26	4.5	1
	0	0	1			
	0	0	1			
	1	1	0	22	4	0
1	0	1	0	31	3	1
	0	0	1			

For peer review only

		0	0	27	4	1
	1	1	0	27	5	1
	0	1	0	27	4	0.5
	1	1	0	23	5	1
	1	1	0	27	4	1
	1	1	0	27	4	1
1		1	0	28	4	0
1		0	0	19	5	1
	0	1	0	27	4	1
	1	1	0	23	4	1
		0	0	26	4	1
	1	1	0	27	4	2
		0	0	28	5	0.25
	0	1	0	28	4.5	1
	1	0	1	19	4	1
	1	0	1	19	5	1
1	1	0	1	19	4	1.5
	0	1	0	26	4	0.75
		0	0	27	4	1
	0	1	0	28	5	1
		0	0	26	5	0
	1	1	0	27	4	1
	0	1	0	30	4	0.75
	0	1	0	27	4	1
	1	1	0	22	4	1
1		0	0	24	4	0.25
	0	0	1	15	3	0
	0	0	1			
		0	0	27	5	1
		0	0	26	4	2
1		0	0	22	4	1
1		0	0	25	5	1
	0	1	0	28	4	1
	0	1	0	27	4	1
	0	1	0	26	4	0.75
	1	1	1	26	4	1
	0	1	0	27	4	1
	0	1	0	31	4	1
1		0	0	28	5	1
	0	0	1			
	0	0	1			
1		0	0	28	4	1
1		0	0	26	3	1
	0	0	1	26	4	1.75
	0	1	0	28	3.5	1
	1	0	1			
	0	0	1			
	0	1	0	27	4	1.5
	0	1	0	27	5	1
	1	1	0	23	5	1

For peer review only

		0	0	28	4	1
1		0	0			
	1	1	0	23	4	1
	1	1	0	23	4	1
	0	0	1			
1	0	1	0	28	5	1
		0	0	26	4	1
	1	1	0	20	4	1
	1	1	0	20	4	1
	1	0	1			
	0	1	0	28	4	1
	0	1	0	26	4	2
	0	1	0	27	4	1
	0	1	0	26	4	1
	0	1	0	27	4	1
	0	1	0	27	5	1
	0	1	0	28	4	2
	1	1	0	21	4	1
	1	0	1			
1	0	1	0	27	5	1
	0	0	1			
	1	1	0	23	4	1.5
	1	1	0	21	4	1
	1	1	0	23	4	1
	1	1	0	22	4.5	1
	0	0	0	23	4	1
	1	1	0	25	5	1
	1	0	0	24	4.5	1
	0	0	0	24	4.5	1
	1	1	0	27	4	1
1	1	1	0	24	5	1
	0	0	1			
		0	0	25	4	2
	0	1	0	16	4.5	1
	0	0	1			
	0	1	0	25	5	0
	0	0	1			
	1	1	0	21	4	1
1		0	0	30	5	0
		0	0	27	4	0.5
	0	1	0	25	3	2
1	0	1	0	18	4	1
	0	0	1			
		0	0	27	4	1
	0	1	0	28	4	1
		0	0	27	4	1
1		1	0	27	4	1
		0	0	20	4	1
1		0	0	29	5	0
1		0	0	27	4	1

For peer review only

	0	1	0	27	5	0
1	0	1	0	32	5	1
	0	0	0	29	4	1
	0	1	0			
1		0	0	27	5	1
1	0	1	0	20	5	1
1		0	0	13	3	1
1		0	0	12	3	0
		0	0	27	4	0.5
	0	1	0	27	5	0
	0	1	0	27	4	0
	0	1	0	27	4	1
		0	0	25	4	1
	0	0	0	24	5	0
	0	1	0	23	5	1
1	1	1	0	23	5	1
	0	0	1			
	0	1	0	24	4	1
	0	1	0	28	5	1
	0	1	0	27	5	1
1	1	0	0	24	5	1
	0	0	1			
	0	0	1			
		0	1			
	0	0	1			
		0	1			
	0	0	1			
	0	0	1			
	1	0	1			
1	1	0	0	24	4	1
	1	0	0	25	5	1
	1	0	1			
	0	1	0	29	5	1
	0	0	1			
	1	0	0	24	4	1
	0	1	0	25	4	1
	0	1	0	25	4	2
	0	1	0	25	4	1
	0	1	0	25	4	0
1		0	0	18	3	1
1	1	0	0	24	5	1
	0	1	0	30	5	1
1		0	0	25	5	1
	0	0	1			
	0	1	0	24	4	1
	0	1	0	25	4	1
	1	0	0	23	4	1
	0	1	0	27	5	0
		1	0	30	5	1
1		1	0	22	5	0.75

For peer review only

	0	1	0	28	5	1
	0	1	0	27	4	1
1	1	0	0	24	5	1
1	0	1	0	26	5	1
1		1	0	26	4	1
	0	0	1			
	0	0	1			
	0	1	0	27	4	0.5
	1	1	0	23	4	1.5
	1	1	0	23	4	1
	0	1	0	26	4.5	1
	0	0	1			
	1	1	0	25	5	1
	0	0	1	19	5	0.5
	0	0	0			
	0	0	0			
	0	1	0	27	4	1
	1	1	0	26	5	1
	0	0	1			
	0	1	0	28	5	1
	0	1	0	27	4	0.5
	1	1	0	25	5	1
	0	1	0	27	4	1
	0	1	0	27	5	1
	0	1	0	31	5	1
	0	1	0	31	4	1
	0	1	0	30	4	1
	0	1	0	29	4	1
1	0	1	0	24	5	1
		0	0	28	4	1
		0	0	27	4	1
1		0	0	28	4	1
	0	1	0	25	5	1
	0	1	0	27	4	1
	0	1	0	27	5	1
	0	0	1			
	1	1	0	26	5	1
	1	1	0	25	4	1
	0	1	0	25	5	1
		0	0	25	4	1
	1	1	0	27	4	1
	0	0	1			
	0	0	1			
	1	1	0	23	5	1
		0	0	28	3	1
	0	0	0	25	4	2
		0	0	26	4	1
	1	1	0	26	5	0
	0	1	0	23	4	0
	0	1	0	23	4	0

For peer review only

	0	1	0	24	5	1
1	1	0	0	25	4	1
	0	0	0	25	5	0
	0	0	0	25	5	1
	1	1	0	24	4	1
	1	0	0	25	5	1
	1	0	0	26	4	1
	1	0	0	25	4	1
	0	0	0	24	5	1
	1	1	0	25	4	1
	0	0	0	24	5	1
	0	1	0	26	5	1
	1	1	0	22	4	1
1	0	0	0	25	5	1
	1	1	0	24	4	0
	1	1	0	26	5	0
	0	0	0	25	5	0
	1	1	0	25	4	2
	0	1	0	25	4	2
	0	1	0	25	4	1
1	0	0	0	25	5	1
1	0	1	0	27	4	1
	0	0	0	26	5	0.75
	0	1	0	25	4	2
	0	1	0	26	4.5	1
	0	1	0	26	4	1
	0	0	0	25	5	1
	0	1	0	25	5	1
	0	0	0	26	5	0
1	0	1	0	25	5	1
	0	0	0	25	5	0
1	0	0	0	25	5	1
	1	1	0	26	5	0
1	0	0	0	25	5	1
1	0	0	0	25	5	0
	0	0	0	26	4	1
	1	1	0	27	5	1
	1	1	0	27	5	1
	0	1	0	27	4	1
	0	1	0	27	4	0.5
	1	1	0	27	5	1
	1	1	0	27	5	1
	1	1	0	27	5	1
	1	0	1			
	1	1	0	27	4	1
1		0	0	28	4	1
		0	0	27	5	0.5
	0	0	1			
1		0	0	28	4	1.25
	0	1	0	27	3	0

For peer review only

	0	1	0	27	4	1
	1	1	0	26	4.5	1
	0	0	1			
	1	1	0	24	4	1
	0	0	1			
		0	0	27	4	0
	0	0	1			
		0	0	24	4.5	1
	1	1	0	27	5	1
1		0	0	20	4	0.25
	0	1	0	31	4	1
		0	0	26	4	1
		0	0	26	4	1
		0	0	22	4	1
		0	0	27	5	1
	0	0	1			
	1	1	0	27	4	1
	1	1	0	27	5	1
1		0	0	30	5	1
	1	1	0	25	5	1
	0	1	0	28	4	1
1		0	0	30	5	0
	0	0	1			
	0	1	0	26	4	1
	1	0	1			
		0	0	26	5	1
	0	1	0	27	5	0
	1	0	1			
1		0	0	26	4	0
		0	0	27	4	1
	0	1	0	27	4	1
	0	0	1			
	1	1	0	27	5	1
	0	1	0	27	4	2
		0	0	27	4	1
	1	1	0	25	4	2
	0	0	1			
1		0	0	28	5	1
	1	0	0	27	5	1
1		0	0	30	3	0.75
	1	1	0	27	5	1
	0	1	0	27	5	1
	0	0	1			
	1	1	0	27	5	1
		0	0	28	5	1
	0	0	1			
	0	1	0	25	4	1
	1	1	0	27	4	1
	1	1	0	25	4	0
	0	0	1			

For peer review only

	0	1	0	27	5	1
	1	1	0	27	5	0.5
	0	1	0	26	5	1
	0	1	0	27	5	0
	0	0	1			
1		0	1			
	1	0	1			
	0	1	0	23	3	2
	1	1	0	27	5	1
	1	1	0	22	4	0
	1	1	0	27	4	1
	1	0	1			
	1	0	1			
	1	1	0	23	4	1
	0	1	0	26	4	1
1	1	0	0	25	5	1
1	0	1	0	26	5	1
		0	0	27	4	0.5
1	0	0	0	23	4	1
	0	0	1			
	0	0	1			
	0	0	1			
	0	0	1			
	0	0	1			
	0	0	1			
	0	0	1	26	4	2
1	0	0	0	29	5	1
	0	1	0	25	4	1
1	1	0	0	25	5	1
	1	0	1			
	0	1	0	26	5	1
	0	1	0	25	5	1
	1	1	0	26	4	1
1	1	0	0	26	5	1
	0	0	1			
	0	0	1			
	0	0	0	24	4	0
	0	0	0	24	5	1
	1	1	0	26	4	1
	1	1	0	27	5	1
		0	0	17	3	1
1		0	0	13	3	0
1	0	1	0	26	4.5	1
		0	0	11	4	1.5
		0	0	19	3	0
	0	1	0	26	4	2
	0	1	0	27	4	1
	1	1	0			
1	0	1	0	25	5	1
1	0	0	0	25	5	1

For peer review only

	1	1	0			
	1	0	1			
	1	0	1			
	0	1	0	27	5	0
1	0	0	0	26	4	1
	0	1	0	26	5	1
	0	1	0	26	5	1
1	1	0	0	25	4	1
1		0	0	26	5	0
	0	0	1			
1		0	0	18	4.5	1
	0	1	0	27	4	1
	0	1	0	25	5	1
		0	0	25	5	1
1		0	0	25	4	1
1	1	0	0	24	4	1
	0	1	0	16	4	0
1		1	0	28	4	1
1	0	0	0	24	5	1
1	0	0	0	25	4	1
1	1	0	0	26	5	1

For peer review only

Min(round	Max(rounc	N(round3)	Median(ro	IQR(round	Min(round	Max(round3)
		19	5	0	4	5
		15	3	1	1	4
		19	4	0	2	5
3	5					
4	5					
3	5					
3	5					
4	5					
		19	5	1	3	5
3	5					
4	5					
3	5					
2	5	19	4	1	1	5
2	5					
3	5					
2	5					
2	4					
1	5					
4	5					
3	5					
3	5					
3	5					
3	5					
		19	4	1.5	3	5
3	5					
2	5	18	3.5	1	2	5
		19	4	1	4	5
3	5					
2	5					
2	5					
		19	4	1.5	2	5
2	5					
3	5					
1	5	15	4	0.5	3	5
2	5					
4	5					
2	4					
3	5					
3	5					
		5	4	1	3	5
		18	4	0	4	5
4	5					
4	5					
		17	4	0	4	5
2	5	8	4	0.25	3	4
3	5					
3	5					
1	5	19	4	1	1	5

For peer review only

3	5					
2	5					
		15	3	1.5	1	4
		18	4	1.75	1	5
		17	4	2	1	5
		18	5	1	1	5
		17	4	2	2	5
		19	4	1	3	5
		19	5	1	4	5
2	5					
4	5					
		18	5	1	4	5
		18	5	0	4	5
		17	4	1	4	5
		18	5	1	3	5
3	5					
4	5					
4	5					
3	5					
2	5					
		13	3	2	1	4
		13	4	1	2	5
		13	5	1	4	5
2	5	6	3.5	1	3	5
1	5	7	5	1	4	5
2	5					
1	5					
2	4	17	3	0	1	4
3	5					
3	5					
3	5					
1	5	17	3	1	3	5
		19	5	0.5	3	5
4	5					
3	5					
		18	4.5	1	4	5
		18	5	1	4	5
		17	4	1	4	5
1	5					
4	5					
3	5	4	3	0.25	3	4
2	5					
4	5					
4	5					
1	5	19	3	0	1	4
4	5					
2	5					
3	5					
2	5	18	4	1	3	5
2	5					

For peer review only

3	5					
3	5					
2	5					
3	5					
1	5	18	3	1	2	5
2	5					
3	5					
		17	3	1	3	5
2	5					
		18	3	1	2	5
3	5					
		12	4	1	3	5
4	5					
4	5					
4	5					
2	5					
1	5					
4	5					
2	5	18	4	1	3	5
3	5					
3	5					
3	5					
1	5	18	4	1	3	5
		18	4	1	4	5
3	5					
2	5	17	4	1	3	5
3	5					
4	5					
4	5					
3	5					
3	5					
3	5					
3	5					
3	5					
		15	4	0	3	5
2	5	19	4	1	3	5
		18	4.5	1	4	5
		7	5	1	3	5
		4	3.5	1.25	3	5
		16	4	1	4	5
4	5					
		17	5	0	2	5
2	5					
4	5					
4	5					
		18	5	1	4	5
		19	5	1	4	5
3	5					
1	5	19	4	1	3	5
		19	4	1	3	5

For peer review only

2	5					
3	5					
2	5					
3	5					
3	5					
3	5					
3	5					
3	5					
2	5					
3	5					
2	5					
2	5					
4	5					
3	5					
3	5					
4	5					
2	5					
3	5					
2	5					
3	5					
3	5					
1	5					
1	5					
1	5					
3	5					
2	5					
2	5					
		18	4	1	3	5
1	5					
1	5	17	4	0	1	5
2	5					
4	5					
3	5					
3	5					
3	5					
3	5					
1	5					
1	5					
2	5					
		13	4	1	2	5
		12	3	0.5	3	5
2	5					
2	5	18	4	0.75	3	5
2	5	17	4	1	3	5
1	5					
		19	4	1	3	5
		19	5	0	3	5
1	5	3	4	0.5	4	5
3	5					
3	5					

For peer review only

2	5					
		17	4	1	2	5
2	5					
3	5					
		19	5	1	4	5
4	5					
3	5					
4	5					
4	5					
		16	5	1	3	5
3	5					
1	5					
3	5					
2	5					
2	5					
3	5					
1	5	19	4	1	1	5
3	5					
		15	4	0.5	2	5
3	5					
		18	5	1	3	5
3	5	18	4	0.75	3	5
3	5					
3	5					
3	5					
4	5					
3	5					
2	5					
4	5					
1	5					
3	5					
		19	5	0	4	5
1	5					
2	5					
		16	4	1	2	5
4	5					
		19	5	0	4	5
3	5					
3	5					
2	5					
1	5	17	3	1	3	5
4	5					
		18	4.5	1	2	5
1	5					
2	5	0				
4	5					
3	5					
2	5					
4	5					
2	5					

For peer review only

4	5					
3	5					
2	5					
		18	4	0	3	5
4	5					
4	5					
3	5	14	3	1	3	4
2	5	14	3	1	3	4
2	5					
4	5	19	5	1	3	5
3	5	19	4	0	3	5
2	5	19	3	0.5	2	4
2	5					
4	5					
4	5					
3	5					
		14	4	0.75	2	5
3	5					
3	5					
4	5					
3	5					
		13	3	1	1	5
		15	3	1	1	5
		15	3	2	1	5
		15	4	1	2	5
		14	4	2	3	5
		14	5	1	3	5
		13	3	1	2	5
		18	4	0	3	5
2	5					
3	5					
		14	4	0	2	5
4	5					
		13	4	1	2	4
2	5					
2	5					
2	5	19	4	1	2	5
3	5					
3	5					
2	4	15	3	1	3	5
2	5					
3	5					
3	5					
		18	4	1	4	5
1	5					
3	5					
2	5					
4	5					
4	5					
3	5					

For peer review only

3	5					
2	5					
4	5					
4	5					
4	5					
		19	5	0	4	5
		19	5	1	4	5
3	5					
2	5	18	4	0	3	5
1	5					
3	5					
		18	4.5	1	4	5
4	5					
4	5					
		17	3	1	1	5
		17	3	1	1	5
3	5					
3	5					
		18	5	1	4	5
4	5					
2	5					
3	5					
2	5					
3	5					
4	5					
2	5					
2	5					
2	5					
3	5					
2	5					
2	5					
3	5					
3	5					
3	5					
4	5					
		19	5	1	3	5
3	5					
3	5					
3	5					
3	5					
1	5					
		17	3	1	2	5
		15	4	1	2	5
4	5					
2	5					
2	5	19	4	0	3	5
2	5					
4	5					
3	5					
3	5					

For peer review only

For peer review only - http://bmjopen.bmj.com/site/about/guidelines.xhtml

4	5					
1	5					
4	5					
3	5					
1	5					
1	5					
3	5					
2	5					
4	5					
3	5					
4	5					
4	5					
1	5					
3	5					
3	5					
4	5					
4	5					
2	5					
2	5	18	4	1	3	5
2	5					
3	5					
1	5					
4	5					
2	5	18	4	1	3	5
3	5					
2	5					
4	5					
4	5					
4	5					
2	5					
4	5					
2	5					
4	5					
2	5					
4	5					
1	5					
4	5					
3	5					
3	5					
3	5					
3	5					
4	5					
3	5					
		17	4	0	3	5
4	5					
2	5					
4	5					
		19	5	1	3	5
2	5	19	4	1	3	5
1	5	19	3	1	2	5

For peer review only

2	5					
4	5					
		3	4	1	3	5
2	5					
		16	4	0	3	5
2	5					
		18	4	1	3	5
3	5					
4	5					
3	4					
1	5					
2	5					
3	5					
3	5					
3	5					
		19	4	1	3	5
1	5					
4	5					
3	5					
4	5					
2	5					
4	5					
		18	5	0	4	5
2	5					
		19	4	2	2	5
3	5					
5	5					
		18	4	1	3	5
3	5					
2	5					
4	5					
		17	4	1	2	5
4	5					
2	5	12	4	0	3	5
2	5					
3	5	16	4	0.25	3	5
		17	4	1	3	5
3	5					
4	5					
1	5	18	3	1	1	5
4	5					
3	5					
		16	3.5	1	1	5
3	5					
3	5					
		18	4.5	1	4	5
1	5					
2	5					
1	5					
		17	5	1	2	5

For peer review only

4	5					
4	5					
1	5					
4	5					
		19	4	0.5	4	5
		19	4	0	2	5
		17	5	1	3	5
2	5	14	2	1	2	4
3	5					
3	5					
4	5					
		18	4	0.75	3	5
		18	5	1	3	5
2	5					
3	5					
3	5					
3	5					
2	5					
3	5					
		16	4	1	3	5
		17	4	1	3	5
		17	4	1	3	5
		15	4	1.5	2	5
		18	4	0.75	2	5
		15	4	1	3	5
3	5	17	4	0	3	5
3	5					
1	5					
4	5					
		17	5	1	2	5
4	5					
2	5					
3	5					
4	5					
		8	4	1.25	3	5
		19	4	0.5	2	5
2	5					
4	5					
3	5					
4	5					
1	5	15	3	1	3	4
1	4	11	3	0	2	3
3	5					
3	5	12	3	1	3	4
2	4	16	3	0	3	5
2	5	19	4	1	3	5
2	5					
		19	5	1	3	5
3	5					
3	5					

For peer review only

		31	4	1	3	5
		19	4	1	3	5
		19	4	1	3	5
4	5					
2	5					
4	5					
4	5					
3	5					
2	5					
		18	4	1	3	5
3	5					
2	5					
3	5					
3	5					
3	5					
3	5					
3	5					
2	5					
3	5					
2	5					
4	5					

For peer review only
